# Novel dual inhibitors of PARP and HDAC induce intratumoral STING-mediated antitumor immunity in triple-negative breast cancer

**DOI:** 10.1038/s41419-023-06303-z

**Published:** 2024-01-05

**Authors:** Qingyun Zhu, Qiuzi Dai, Lei Zhao, Chang Zheng, Qinyuan Li, Zigao Yuan, Lulu Li, Zhuoye Xie, Zixuan Qiu, Wenjun Huang, Guowen Liu, Xuyu Zu, Bizhu Chu, Yuyang Jiang

**Affiliations:** 1 The First Affiliated Hospital, Cancer Research Institute, Hengyang Medical School, University of South China, Hengyang, 421001 China; 2 School of Pharmacy, Shenzhen University Medical School, Shenzhen University, Shenzhen, 518055 China; 3 State Key Laboratory of Chemical Oncogenomics, Tsinghua Shenzhen International Graduate School, Shenzhen, 518055 China; 4 Academics Working Station, Hunan Key Laboratory of the Research and Development of Novel Pharmaceutical Preparations, Changsha Medical University, Changsha, 410219 China; 5 Department of Breast and Thyroid Surgery, Second People’s Hospital of Shenzhen, First Affiliated Hospital of Shenzhen University, Shenzhen, 518035 China; 6 Institute of Biomedical Health Technology and Engineering, Shenzhen Bay Laboratory, Shenzhen, 518132 China; 7 School of Pharmaceutical Sciences, Tsinghua University, Beijing, 100084 China

**Keywords:** Pharmacodynamics, Breast cancer

## Abstract

PARP inhibitors and HDAC inhibitors have been approved for the clinical treatment of malignancies, but acquired resistance of or limited effects on solid tumors with a single agent remain as challenges. Bioinformatics analyses and a combination of experiments had demonstrated the synergistic effects of PARP and HDAC inhibitors in triple-negative breast cancer. A series of novel dual PARP and HDAC inhibitors were rationally designed and synthesized, and these molecules exhibited high enzyme inhibition activity with excellent antitumor effects in vitro and in vivo. Mechanistically, dual PARP and HDAC inhibitors induced BRCAness to restore synthetic lethality and promoted cytosolic DNA accumulation, which further activates the cGAS–STING pathway and produces proinflammatory chemokines through type I IFN-mediated JAK–STAT pathway. Moreover, these inhibitors promoted neoantigen generation, upregulated antigen presentation genes and PD-L1, and enhanced antitumor immunity when combined with immune checkpoint blockade therapy. These results indicated that novel dual PARP and HDAC inhibitors have antitumor immunomodulatory functions in triple-negative breast cancer.

**Figure:**
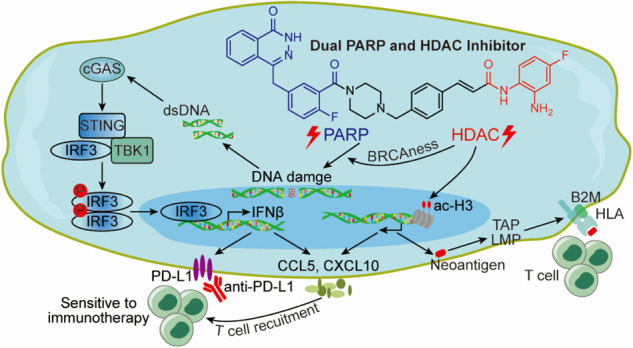
Novel dual PARP and HDAC inhibitors induce BRCAness to restore synthetic lethality, activating tumoral IFN signaling via the cGAS–STING pathway and inducing cytokine production, promoting neoantigen generation and presentation to enhance the immune response.

Novel dual PARP and HDAC inhibitors induce BRCAness to restore synthetic lethality, activating tumoral IFN signaling via the cGAS–STING pathway and inducing cytokine production, promoting neoantigen generation and presentation to enhance the immune response.

## Introduction

The poly(adenosine diphosphate (ADP)-ribose) polymerase (PARP) family includes 17 members that can be activated upon binding to damaged DNA and are primary proteins involved in single-strand DNA break (SSB) repair. Damaged DNA at SSBs provides a binding site for PARP, which allosterically induces PARP catalytic activity [1], leading to poly(ADP-ribosyl)ation (PARylation) of substrate proteins, recruitment of DNA repair protein complexes, chromatin remodeling, and eventually DNA repair [2]. PARP inhibitors (PARPis) bind the catalytic domain of PARP, “trapping” PARP at the DNA damage binding site and inhibiting SSB repair. These unrepaired SSBs convert to DNA double-strand breaks (DSBs) during the S phase of the cell cycle. DSBs can be repaired via homologous recombination repair (HRR), and both breast cancer susceptibility gene 1 (BRCA1) and breast cancer susceptibility gene 2 (BRCA2) proteins are critical components of HRR. Consequently, PARPis, such as olaparib, has been approved by the Food and Drug Administration (FDA) for treating patients, with breast and pancreatic cancers, who have germline BRCA1 or BRCA2 mutations based on the concept of synthetic lethality [3–6].

Despite this promising therapeutic efficacy, innate or acquired resistance to single-agent PARPis occurs, which has led to the optimal use of PARPis within drug combination strategies to sensitize or resensitize cancer cells [7]. Histone deacetylase (HDAC) inhibitors have been reported to improve PARPis efficacy by downregulating HRR proteins to restore synthetic lethality. They can also suppress PARP-mediated PARylation of DNA repair proteins, providing the rationale for combining PARPis with histone modification inhibitors [8–10]. Aberrant HDAC expression correlates with a significantly poorer outcome in various cancer types [11], which implies the therapeutic effect of HDAC inhibitors (HDACis) in tumors. Clinically, single-agent HDACis are approved for hematological malignancies [12]. HDACis has not been effective in treating solid tumors, but the mechanisms are still unclear [13]. A recent study suggested that combinations of agents involving different mechanisms of action can overcome acquired resistance and provide new treatments [14]. Therefore, the combined use of PARPis and HDACis has significant clinical and biological implications, and a small number of studies have been performed to explore the combination of these inhibitors [8–10, 15, 16].

Recent studies have reported that PARPis activate the cyclic GMP-AMP synthetase (cGAS)–stimulator of interferon genes (STING) signaling pathway to induce antitumor immunity [17–19]. PARPis, promoting the accumulation of cytosolic DNA fragments caused by the toxic DNA DSBs, activate the cGAS–STING pathway, leading to type I interferon (IFN) production. These IFNs bind to the type I IFN receptor and activate the downstream Janus kinase (JAK)–signal transducer and activator of transcription (STAT) signaling pathway, resulting in the expression of hundreds of IFN-stimulated genes and subsequent production of proinflammatory chemokines to increase T-cell infiltration [20, 21]. In addition, PARPis increase the tumor mutational burden (TMB), which could lead to neoantigen generation and enhanced anticancer T-cell activity. HDACis also bring about a number of immunomodulatory activities, including the upregulation of major histocompatibility complex (MHC) class I and class II antigen-processing and presentation genes [22–24] and the induction of the expression of multiple T-cell chemokines, such as CCL5, CXCL9, and CXCL10 [25]. Furthermore, PARPis and HDACis increase the expression of PD-L1, marker of immune feedback regulation and immune exhaustion [26, 27]. Thus, these inhibitors have been shown to potently mediate effective immune responses by a synergistic effect with PD-L1 or PD-1 blockade in triple-negative breast cancer (TNBC) or other cancers [26, 27]. Overall, these findings suggest that a single-agent PARPi or HDACi might face resistance or have minimal effects on solid tumors, whereas the synergistic effect of PARPi and HDACi cotreatment was observed. The combined use of PARPis and HDACis is able to mediate immunomodulatory functions and could be a potential therapeutic approach to TNBC.

In this study, we identified a positive correlation between the expression levels of PARP and HDAC in human breast cancer. We designed and synthesized a series of novel benzamide derivatives of olaparib as dual PARP and HDAC inhibitors (termed PARP/HDACis) and demonstrated that PARP/HDACis could downregulate the HRR proteins BRCA1 and RAD51, implying the restoration of synthetic lethality. PARP/HDACis exhibited antitumor properties by inducing antiproliferation and apoptosis of TNBC cells and reducing their abilities of migration and invasion. PARP/HDACis also activated tumoral type I IFN signaling through the cGAS–STING pathway and induced proinflammatory chemokine production through the IFN receptor-mediated JAK–STAT signaling pathway. Moreover, PARP/HDACis enhanced neoantigen generation, including upregulation of MHC class I antigen-processing and presentation genes and PD-L1 expression, enhancing antitumor immunity when combined with anti-PD-L1 in the immunocompetent mouse model.

## Results

### Positive correlation between PARP and HDAC in human breast cancer

**Fig. 1:**
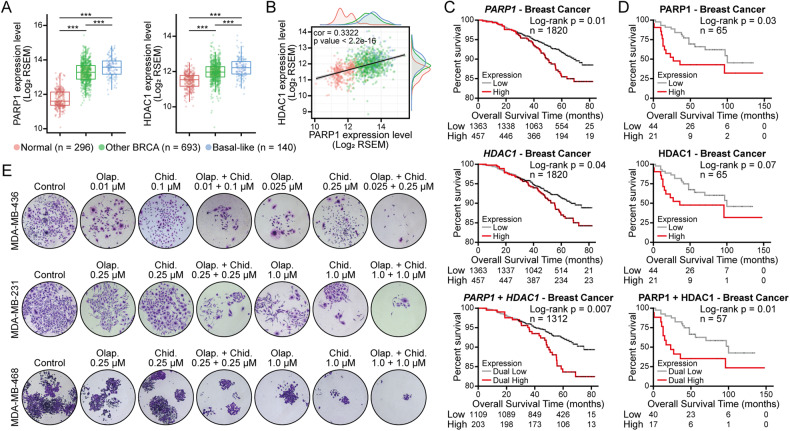
Human breast tissue expression of PARP and HDAC and its correlation and synergistic anticancer efficacy in vitro. **A** Transcript expression levels of PARP1 and HDAC1 in different breast samples from TCGA TARGET GTEx study (Normal: normal tissues; Other BRCA: other breast cancer tissues, non-basal-like subtype tissues; Basal-like: basal-like subtype tissues). ***p < 0.001. **B** Correlation of PARP1 and HDAC1 transcriptomic expression levels in human breast cancer tissues and normal tissues (normal tissues, *n* = 296; other BRCA tissues, *n* = 693; basal-like subtype tissues, *n* = 140). **C**, **D** Kaplan–Meier overall survival curves of human breast tumors according to PARP1 and HDAC1 gene **C** or protein **D** expression levels, with auto select best cut-off selected. Differences were assessed using the log-rank (Mantel–Cox) test. **E** MDA-MB-436, MDA-MB-231, and MDA-MB-468 cells were treated with different concentrations of PARP inhibitors (olaparib) and HDAC inhibitors (chidamide) alone or in combination for approximately 14 days, and cell growth was measured by colony formation assay. Olap., olaparib; Chid., chidamide.

To validate the synergistic effects of PARPi and HDACi treatment, we first performed PARP and HDAC expression and correlation analysis with The Cancer Genome Atlas (TCGA) database. The results showed that PARP1 and HDAC1 were markedly overexpressed in human breast cancers relative to healthy tissues, especially in TNBC (Fig. 1A). A positive correlation was also observed between the expression levels of PARP1 and HDAC1 (Fig. 1B). High PARP1 and HDAC1 gene (Fig. 1C) and protein (Fig. 1D) expression was associated with poor prognosis. Additionally, the co-expression of PARP1 with HDAC1 was significantly associated with a worse prognosis. To validate the synergy of PARPi and HDACi, breast cancer cell lines were treated with different concentrations of PARPi olaparib and HDACi chidamide alone or in combination. The colony formation assay results showed that the addition of chidamide could enhance the efficacy of olaparib in breast cancer cell lines (Fig. 1E). Moreover, a low dose of the PARPi olaparib reduced the clonogenicity of BRCA1- and HRR-deficient MDA-MB-436 cells, while this effect was not observed in MDA-MB-231 (BRCA1 wild-type and HRR-proficient cell lines) and MDA-MB-468 (BRCA1 wild-type but showing a BRCA2 missense mutation) [21, 28]. These results suggest that PARPi can exhibit antitumor effects based on synthetic lethality. Furthermore, chidamide, as an HDACi approved by the China National Medical Products Administration (NMPA) for treating hematological malignancies, has a limiting inhibitory effect on solid tumors. Collectively, these findings demonstrated that the expression levels of PARP and HDAC are increased in breast cancer, especially in TNBC, and positively correlated. The PARPi and HDACi also showed a synergistic effect in suppressing breast cancer cell lines.

### Design and synthesis of olaparib-based benzamide derivatives as PARP/HDACis

**Fig. 2:**
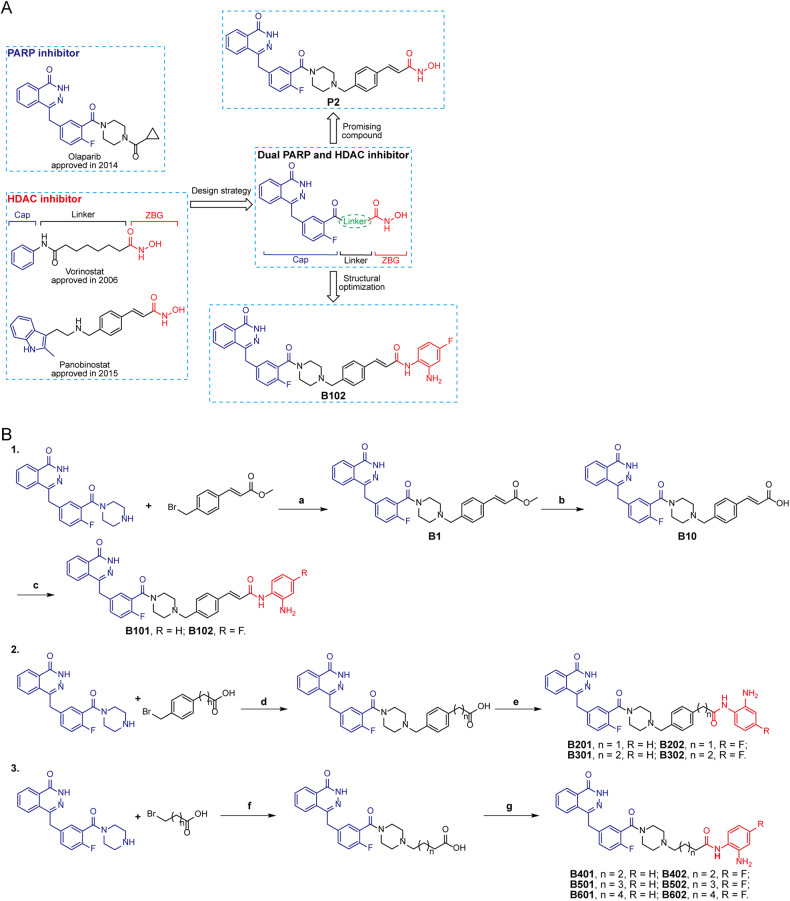
Design and structural optimization and synthesis strategies of dual PARP and HDAC inhibitors. **A** Design and structural optimization strategies of dual PARP and HDAC inhibitors. **B** Synthesis route of designed novel dual PARP and HDAC inhibitors. Reagents and conditions: **a** DIEA, MeCN, rt, 2 h; **b** LiOH, THF/H_2_O, rt, 3 h; **c**, **e**, **g** anilines, DIEA, HATU, DMF, rt, 2 h; **d**, **f** DIEA, MeCN, reflux, 2 h.

Findings above prompted us to design and synthesize a series of novel benzamide derivatives of olaparib as dual PARP and HDAC inhibitors. Structure-activity relationships have confirmed that the phthalazinone structure of olaparib would increase binding affinity with PARP and that the piperazine moiety has been mainly used to improve the PARP inhibitory activity or optimize the physical and chemical properties. Based on a rational drug design strategy, we have developed olaparib hydroxamic acid derivatives as first-in-class PARP/HDACis and showed robust enzymatic activity against PARP and HDAC in vitro [15]. However, hydroxamic acid derivatives usually have limitations in terms of oral bioavailability and metabolic stability. Therefore, medicinal chemists have tried to develop other weaker Zn^2+^ binding groups to replace hydroxamic acid to reduce the toxicity and side effects of HDAC inhibitors. The benzamide derivative chidamide is an appropriate and effective zinc-binding group (ZBG). Herein, we developed a series of novel benzamide derivatives of olaparib as PARP/HDACis (Fig. 2A). We proposed that different modifications of the piperazine moiety of olaparib have little influence on its inhibitory effect against PARP1. Our designed compounds possessed three pharmacophore characteristics of HDAC inhibitors: phthalazinone of olaparib acts as the cap group, benzamide acts as the ZBG, and a linker connects the cap group and ZBG (Fig. 2A). The synthesis strategies of dual PARP and HDAC inhibitors are shown in Fig. 2B.

### PARP/HDACi enzymatic activity and cytotoxicity

First, the inhibitory effect of the twelve synthesized dual PARP and HDAC inhibitors on PARP1, PARP2, HDAC1, and HDAC6 were investigated using an enzymatic assay. PARP inhibitor olaparib, pan-HDAC inhibitor vorinostat, and HDAC subtype-selective inhibitor chidamide were used as positive controls. As expected, our compounds **B101**–**B202** were able to inhibited PARP1, PARP2 and HDAC1 (Table 1). Compounds **B101**–**B302**, which maintained the aromatic linker group in the phthalazinone, had weaker inhibitory effect on PARP1 than olaparib. Compound **B102** (IC_50_ = 19.01 nM) contains the terminal ZBG 4-fluoro-*o*-phenylenediamine, and its PARP1 enzymatic activity was lower than that of compound **B101** (IC_50_ = 13.15 nM) without fluorine atoms of *o*-phenylenediamine. Compounds **B201**–**B302** have a similar PARP1 inhibitory enzymatic activity compared to that of compound **B101**. Compounds **B101** and **B102** inhibited PARP2 activity and were weaker than olaparib. Besides, compounds **B201**–**B302** (IC_50_ > 13.78 nM) showed much lower PARP2 inhibition activity than olaparib (IC_50_ = 0.08 nM). Therefore, the aromatic conjugated linker in the chemical structures of these compounds might help to maintain PARP1 and PARP2 inhibitory ability.

**Table 1:** Enzymatic activity of PARP/HDAC dual inhibitor **B101**–**B602** against PARP1/2 and HDAC1/6.

Cpds.	IC_50_ (nM)		IC_50_ (μM)	
	PARP1	PARP2	HDAC1	HDAC6
**B101**	13.15	4.08	0.12	> 10
**B102**	19.01	2.13	1.69	> 10
**B201**	7.55	13.78	0.06	> 10
**B202**	10.25	34.18	0.16	> 10
**B301**	11.43	70.39	> 10	> 10
**B302**	12.67	56.98	> 10	> 10
**B401**	ND	ND	> 10	> 10
**B402**	ND	ND	> 10	> 10
**B501**	ND	ND	> 10	> 10
**B502**	ND	ND	> 10	> 10
**B601**	ND	ND	> 10	> 10
**B602**	ND	ND	> 10	> 10
Olaparib	0.35	0.08	ND	ND
Vorinostat	ND	ND	0.01	0.04
Chidamide	ND	ND	0.04	> 10

*ND* compound not detected against the enzyme.

The synthesized compounds **B101**–**B602** were also tested against two HDAC isoforms, HDAC1 from class I (HDAC1, 2, 3, 8) and HDAC6 from class IIb (HDAC6, 10). Previous studies have reported that HDAC1 and HDAC6, as representative subtypes, are sensitive to interactions with molecules and Zn binding pocket [29, 30]. Compounds **B102**–**B202** exhibited similar inhibitory activity against HDAC1 compared with chidamide (Table 1). Note that, IC_50_ value of compound **B201** on HDAC1 was approximate to that of chidamide, while compounds **B101,** **B102**, and **B202** showed less potent HDAC1 inhibitory activities than chidamide. As we designed, all compounds, such as the subtype-selective inhibitor chidamide, showed a low effect on HDAC1. Considering the hydrophobicity and size of these unoccupied pockets of the HDAC enzyme, different linker lengths were closely related to the HDAC-inhibitory potency via occupation of the hydrophobic channel. It has been reported that HDAC6 provides a conserved binding site via a wider and shallower catalytic channel than HDAC1. Therefore, shorter linkers of compounds can achieve HDAC6 isoform-selective inhibition. We speculated that the linker lengths of **B101**–**B202** were more suitable for binding HDAC1.

Given the above enzymatic activity results, the anticancer effects of **B101**–**B302** on human breast cancer cell lines were evaluated in vitro. As shown in Table 2, PARP/HDACi markedly reduced the viability of TNBC cells with IC_50_ values ranging from 0.16 μM to 4.12 μM. We also found that the IC_50_ values of most PARP/HDACis in TNBC cells were much lower than those of the FDA-approved PARPi (olaparib) and HDACi (vorinostat, chidamide). The IC_50_ values of PARP/HDACis for estrogen receptor-positive MCF-7 cells were higher than those for TNBC cells, and PARP/HDACis showed a much weaker effect on normal breast cell MCF-10A viability. These results suggested that PARP/HDACis might have strong antitumorigenic activity against TNBC and low cytotoxicity towards normal breast cell lines.

**Table 2:** Anti-proliferative activity of PARP/HDAC dual inhibitor **B101**–**B302** in human breast cancer cell lines and human breast cell line.

Cpds	IC_50_ (μM)				
	MDA-MB-436	MDA-MB-231	MDA-MB-468	MCF-7	MCF-10A
**B101**	0.53 ± 0.08	0.41 ± 0.01	0.30 ± 0.05	3.62 ± 1.21	1.45 ± 0.09
**B102**	0.16 ± 0.05	0.16 ± 0.02	0.27 ± 0.11	13.36 ± 3.20	13.66 ± 0.23
**B201**	0.97 ± 0.73	0.38 ± 0.08	1.35 ± 0.55	5.30 ± 1.23	14.00 ± 2.45
**B202**	0.65 ± 0.14	0.46 ± 0.12	0.56 ± 0.10	15.6 ± 3.52	9.83 ± 0.75
**B301**	1.73 ± 0.82	1.96 ± 0.63	1.75 ± 0.37	3.53 ± 1.11	30.05 ± 9.80
**B302**	4.12 ± 1.02	2.83 ± 0.40	1.27 ± 0.58	4.93 ± 1.09	36.82 ± 6.59
Olaparib	8.08 ± 2.02	3.24 ± 2.20	14.48 ± 2.44	> 25	27.98 ± 3.48
Vorinostat	4.29 ± 1.25	2.59 ± 0.27	3.36 ± 1.21	0.98 ± 0.32	6.40 ± 2.72
Chidamide	4.15 ± 0.92	2.34 ± 0.92	0.75 ± 0.22	4.60 ± 0.78	> 50

Data are expressed as the mean ± SD from the dose-response curves of at least three independent experiments.

### PARP/HDACis induce DNA damage and inhibit HRR gene expression to restore synthetic lethality

**Fig. 3:**
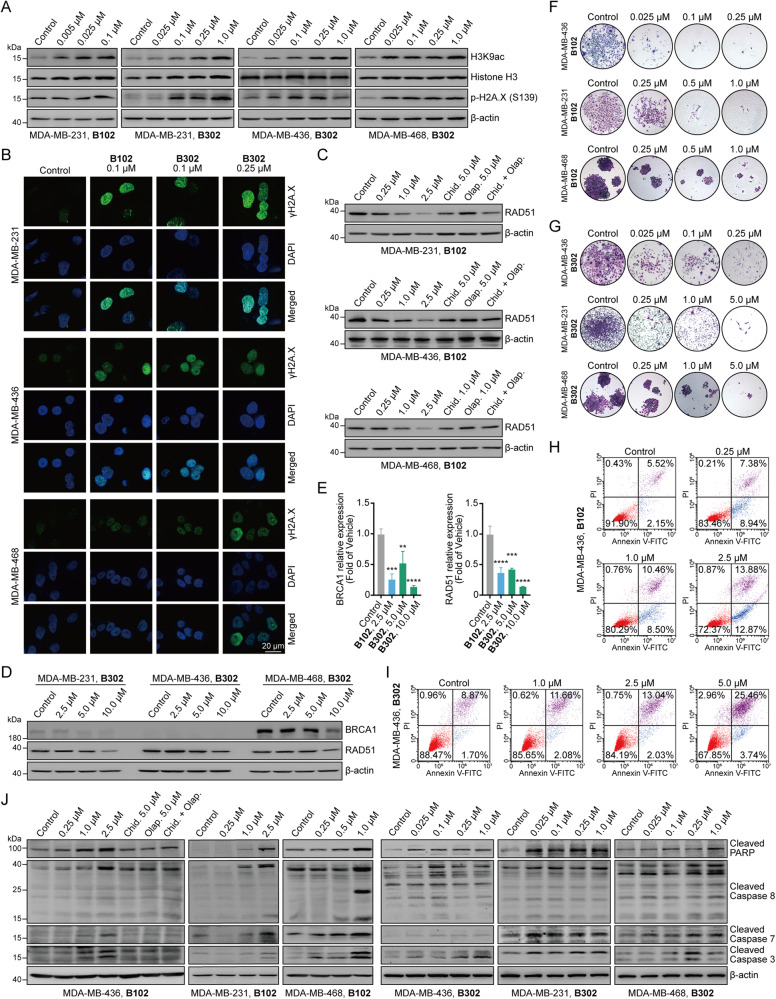
Dual PARP and HDAC inhibitors induce DNA damage, inhibit the expression of key proteins encoding HRR genes, inhibit cell proliferation, and induce apoptosis in vitro. **A** The TNBC cell lines MDA-MB-231, MDA-MB-436, and MDA-MB-468 were treated with **B102** or **B302** at different concentrations for 48 h, and the cell lysates were analyzed by western blotting with the indicated antibodies. **B** MDA-MB-231, MDA-MB-436, and MDA-MB-468 cells were treated with **B102** or **B302** at different concentrations for 48 h, fixed, and then subjected to immunofluorescence analysis for γH2A.X; scale bars, 20 μm. **C** MDA-MB-231, MDA-MB-436, and MDA-MB-468 cells were treated with **B102** at different concentrations, olaparib, chidamide, or olaparib in combination with chidamide for 24 h, and the cell lysates were analyzed by western blotting with the antibodies indicated. **D** MDA-MB-231, MDA-MB-436, and MDA-MB-468 cells were treated with **B302** at different concentrations for 24 h, and the cell lysates were analyzed by western blotting with the indicated antibodies. **E** PARP/HDACi suppressed the mRNA expression of BRAC1 and RAD51. MDA-MB-231 cells were treated with **B102** or **B302** at different concentrations for 24 h. The relative mRNA expression levels of BRAC1 and RAD51 were determined by RT‒PCR assay. Data are presented as the mean ± standard deviation (SD), unpaired Student’s *t* test, *n* = 3; **p < 0.01; ***p < 0.001; ****p < 0.0001. **F**, **G** **B102** and **B302** inhibited the clonogenicity of TNBC cells in a dose-dependent manner. Representative colony plates of three independent experiments are shown. Magnification: ×50. **H**, **I** MDA-MB-436 cells were treated with **B102** **H** or **B302** **I** at the indicated concentrations for 48 h, and cells were examined with an Annexin V-FITC/PI apoptosis detection kit to detect cell apoptosis with flow cytometry. Representative results of three independent experiments are shown. **J** MDA-MB-231, MDA-MB-436, and MDA-MB-468 cells were treated with **B102** or **B302** at different concentrations, olaparib, chidamide, or olaparib in combination with chidamide for 24 h, and the cell lysates were analyzed by western blotting with apoptosis-related antibodies.

PARPis can induce DNA damage, which is detected by increased H2A.X phosphorylation (γH2A.X) [18]. PARP/HDACi **B102** and **B302** had increased p-H2A.X (S136) levels, indicating the generation of DNA DSBs when PARP was inhibited (Fig. 3A). Formation of γH2A.X foci was also detected by immunofluorescence analyses (Fig. 3B). Treatment with **B102** and **B302** also elevated the acetylation of lysine 9 of histone H3 (H3K9), suggesting that PARP/HDACis have HDAC-inhibitory activity (Fig. 3A).

Based on the concept of synthetic lethality, PARPis exert their therapeutic effects on patients with BRCA1/2 mutant cancers. However, acquired resistance to PARPi arises partially owing to the secondary mutations of HRR genes or proteins, including BRCA1/2 and RAD51, which restored DSB repair capacity and negates synthetic lethality [31, 32]. HDACis have been reported to downregulate HRR gene or protein expression [8, 9, 33], and our results showed that the HDACi chidamide alone reduced the expression of RAD51 (Fig. 3C). The combination of chidamide and the PARPi olaparib also decreased RAD51 expression but did not show synergistic inhibitory effects in all TNBC cell lines (Fig. 3C), suggesting that a single-agent of PARPi did not regulate RAD51 expression. Furthermore, the PARP/HDACi led to a reduction in RAD51 and BRCA1 gene and protein expression (Fig. 3D, E), and **B102** had more potent inhibitory effect on RAD51 at 2.5 μM compared with the positive controls at 5 μM (Fig. 3C). These data demonstrated that the PARP/HDACi may activate an antitumor mechanism by inducing DNA damage and decreasing the DNA DSB repair capacity to restore synthetic lethality.

### PARP/HDACi treatment leads to cell growth inhibition and apoptosis in human TNBC cells

To assess the potential of PARP/HDACis as antitumor therapeutic agents in vitro, colony formation assay was first evaluated in TNBC cells. The results showed a reduction in colony formation from cells cultured with **B102** and **B302** (Fig. 3F, G). **B102**, which has a lower IC_50_ value, inhibited colony formation more effectively than **B302** at the same dose, consistent with their respective levels of cytotoxicity (Table 2).

Next, we confirmed the ability of **B102** and **B302** to induce apoptosis in TNBC cells by flow cytometry (Fig. 3H, I). Mechanistically, PARP/HDACi treatment induced specific cleavage of caspase-8 and caspase-7 levels in a concentration-dependent manner and subsequent activation of caspase-3 and PARP (Fig. 3J). These results indicate that PARP/HDACi treatment elicits the mitochondrial-dependent intrinsic apoptosis pathway and caspase-8-mediated extrinsic pathways. Overall, these results proved that PARP/HDACis exhibit antitumor properties by reducing proliferation and inducing apoptosis of TNBC cells.

### PARP/HDACi treatment inhibits the migration and invasion of human breast cancer cells

**Fig. 4:**
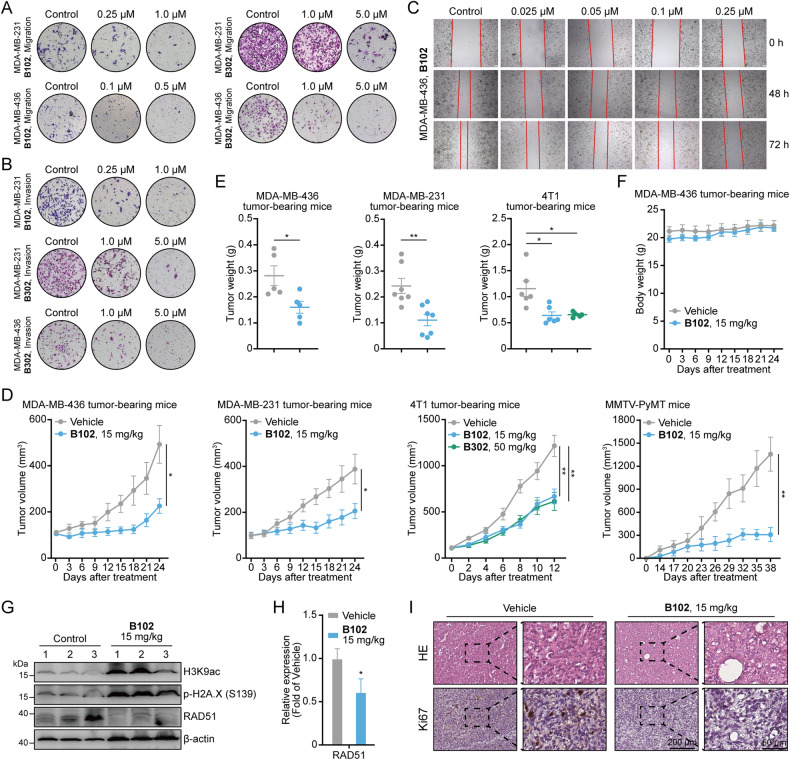
Dual PARP and HDAC inhibitors inhibit migration and invasion activity in vitro and exert an antitumor effect in vivo. **A** Transwell assay to detect the migration ability of TNBC cells treated with different concentrations of **B102** or **B302** for 48 h. **B** Transwell assay to detect the invasion ability of TNBC cells treated with different concentrations of **B102** or **B302** for 48 h. Representative images of migrated or invaded cells on polycarbonate transwell membranes of three independent experiments are shown. **C** Wound healing assay of MDA-MB-436 cells treated with **B102** at different concentrations. **D** MDA-MB-436, MDA-MB-231, 4T1 tumor-bearing mice, and MMTV-PyMT mice were treated with vehicle, **B102** (15 mg/kg) or **B302** (50 mg/kg). The tumor growth curve was plotted as tumor volume versus time since treatment. Error bars represent the means ± standard error of the means (SEMs), unpaired Student’s *t* test, *n* ≥ 5; *p < 0.05; **p < 0.01. **E** The tumor weight at the end of the experiments is shown. Error bars represent the means ± SEMs, unpaired Student’s *t* test, *n* ≥ 5; *p < 0.05; **p < 0.01. **F** The body weights of tumor-bearing mice were not obviously altered after treatment with **B102** (15 mg/kg/d). Error bars represent the means ± SEMs, unpaired Student’s *t* test, *n* ≥ 5. **G** MDA-MB-436 tumor-bearing mouse tissue lysates were analyzed by western blotting with the indicated antibodies. *n* = 3. **H** The relative mRNA expression levels of RAD51 were determined by RT‒PCR assay in MDA-MB-436 tumor-bearing mice. Data are presented as the mean ± SD, unpaired Student’s *t* test, *n* = 3; *p < 0.05. **I** Representative images of HE and Ki67 stained in MDA-MB-436 xenograft mouse tumors; scale bars, 200 μm or 50 μm.

Among breast cancer types, TNBC exhibits extremely high metastatic and invasive activity [34]. We further explored the effect of PARP/HDACi treatment on the migration and invasion activity of TNBC cells in vitro by Transwell assay. Compared with the vehicle, **B102** and **B302** considerably inhibited the migration of TNBC cells in vitro (Fig. 4A). Furthermore, after PARP/HDACi treatment, the cells in the upper chambers showed reduced invasive ability (Fig. 4B). Results of wound healing assay further confirmed that PARP/HDACi treatment can reduce the migration of the cells (Fig. 4C and Fig. S1A). Together, these results revealed that PARP/HDACis are capable of suppressing the migration and invasion of TNBC cells in vitro.

### PARP/HDACi exerts an antitumor effect in vivo

To assess the potential of PARP/HDACi as a tumor therapeutic agent in vivo, we administered **B102** and **B302** to MDA-MB-436, MDA-MB-231, and 4T1 cell-derived xenografts. After treatment, tumors derived from PARP/HDACi-treated mice displayed significantly slower growth rates than those derived from the vehicle group (Fig. 4D and Fig. S1B–E). Besides, compared to the **B302**-treated groups, the **B102**-treated group showed robust inhibition of tumor growth at low doses, which is consistent with their respective cytotoxicity (Table 2). In addition, tumor weight was much lower in PARP/HDACi-treated mice than in vehicle-treated mice (Fig. 4E). Moreover, we did not observe any body weight loss or other signs of toxicity in mice treated with PARP/HDACi (Fig. 4F and Fig. S1F). The antitumor effect was also observed in the mouse mammary tumor virus (MMTV) promoter drives the polyomavirus middle T antigen (PyMT) breast cancer mouse models, which mimics all identifiable stages of human breast cancer progression (Fig. 4D and Fig. S1F). Mechanistically, PARP/HDACi increased the acetylation of H3K9 and H2A.X phosphorylation and reduced the expression of RAD51 and BRAC1 on tumor cells in MDA-MB-436 and 4T1 mice (Fig. 4G, H and Fig. S1G), suggesting that the antitumor effect in vivo coincides with disruption of PARP and HDAC enzymatic activity. Hematoxylin and eosin (HE) and Ki67 staining further demonstrated a decrease in the Ki67-positive staining in MDA-MB-436 xenograft mice upon PARP/HDACi treatment (Fig. 4I). These results demonstrated that PARP/HDACis exerts an antitumor effect in vivo and might be well-tolerated TNBC treatments.

### PARP/HDACi treatment produces type I IFNs through the cGAS–STING pathway

**Fig. 5:**
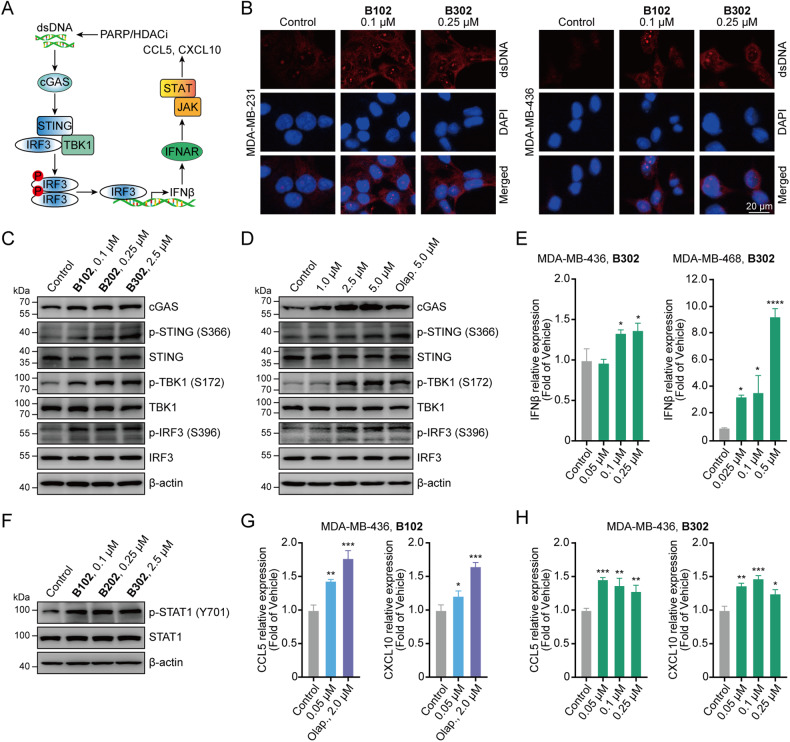
Dual PARP and HDAC inhibitors restore type I IFN signaling via the cGAS–STING pathway, leading to the production of proinflammatory chemokines. **A** Schematic illustration of dual PARP and HDAC inhibitors activated the cGAS–STING pathway. **B** MDA-MB-231 and MDA-MB-436 cells were treated with **B102** or **B302** for 48 h, fixed, and then subjected to immunofluorescence analysis for dsDNA; scale bars, 20 μm. **C** MDA-MB-436 cells were treated with **B102,** **B202**, or **B302** for 48 h, and the cell lysates were analyzed by western blotting with cGAS–STING pathway antibodies. **D** **B302** treatment resulted in concentration-dependent activation of the cGAS–STING signaling pathway. MDA-MB-436 cells were treated with **B302** at different concentrations for 48 h, and the cell lysates were analyzed by western blotting with cGAS–STING pathway antibodies. **E** MDA-MB-436 and MDA-MB-468 cells were treated with **B302** at different concentrations for 48 h. The relative mRNA expression levels of IFNβ were determined by RT‒PCR assay. **F** PARP/HDACi increases IFNβ–induced STAT1 phosphorylation. MDA-MB-436 cells were treated with **B102,** **B202**, or **B302** for 48 h, and the cell lysates were analyzed by western blotting with the indicated antibodies. **G** MDA-MB-436 cells were treated with **B102** or olaparib for 48 h. The relative mRNA expression levels of CCL5 and CXCL10 were determined by RT‒PCR assay. Olap., olaparib. **H** MDA-MB-436 cells were treated with **B302** at different concentrations for 48 h. The relative mRNA expression levels of CCL5 and CXCL10 were determined by RT‒PCR assay. Data are presented as the mean ± SD, unpaired Student’s *t* test, *n* = 3; *p < 0.05; **p < 0.01; ***p < 0.001; ****p < 0.0001.

PARP/HDACi treatment markedly increased γH2A.X levels (Fig. 3A, B), induced DNA DSBs, and led to the accumulation of cytosolic DNA fragments. Thus, we examined whether PARP/HDACi can induce the accumulation of cytosolic DNA, which could activate the cGAS–STING signaling pathway and induce the transcription of type I IFNs in breast cancer cells (Fig. 5A). We found that treatment with PARP/HDACi induced cytosolic DNA accumulation (Fig. 5B), and activated the cGAS–STING signaling pathway by inducing the phosphorylation of STING at the Ser366 site [p-STING (S366)], p-TBK1 (S172) and p-IRF3 (S396) in MDA-MB-436 cells (Fig. 5C). Also, treatment with **B302** led to concentration-dependent activation of the cGAS–STING signaling pathway (Fig. 5D).

IRF3 has been previously reported to translocate to the nucleus as a transcription factor to induce robust transcription of type I IFN genes, particularly IFNβ [35, 36]. Treatment with PARP/HDACi significantly upregulated the mRNA levels of IFNβ in TNBC cells and MDA-MB-436 tumor-bearing mice (Fig. 5E and Fig. S1H). IFNβ bound to IFNAR and activated the downstream JAK–STAT pathway [37]. We found that PARP/HDACi treatment restored type I IFN signaling in MDA-MB-436 cells as measured by induced STAT1 phosphorylation (Fig. 5F). Activation of the JAK–STAT signaling pathway leads to the production of proinflammatory chemokines, such as CCL5 and CXCL10, to increase T-cell infiltration [20, 21, 38, 39]. Our results showed that PARP/HDACi treatment augmented the mRNA levels of CCL5 and CXCL10 in three different TNBC cell lines and MDA-MB-436 tumor-bearing mice (Fig. 5G, H and Fig. S2). Similar to PARP/HDACi treatment, the rise in the mRNA levels of CCL5 and CXCL10 was observed in MDA-MB-436 and MDA-MB-468 cells treated with the PARPi olaparib (Fig. 5G and Fig. S2A). These results implied that PARP/HDACi potently activates the cGAS–STING signaling pathway in TNBC cells, resulting in subsequent activation of TBK1–IRF3 pathway and producing proinflammatory chemokines through type I IFN signaling.

### PARP/HDACi upregulates neoantigen generation, antigen-processing, and presentation genes, and PD-L1

**Fig. 6:**
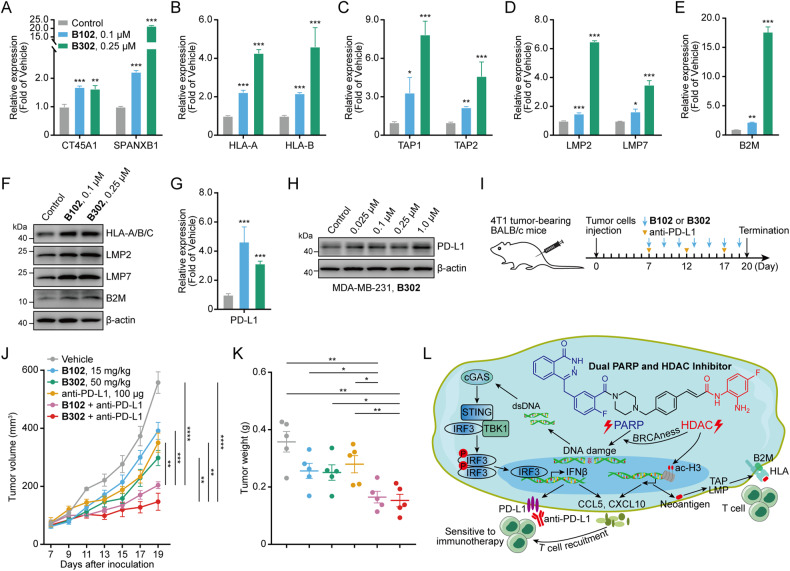
Dual PARP and HDAC inhibitors promote neoantigen generation and upregulate antigen-processing and presentation genes and PD-L1. **A** MDA-MB-231 cells were treated with **B102** or **B302** for 48 h. The relative mRNA expression levels of neoantigens were determined by RT‒PCR assay. **B**–**E** MDA-MB-231 cells were treated with **B102** or **B302** for 48 h. The relative mRNA expression levels of antigen-processing and presentation genes were determined by RT‒PCR assay. **F** MDA-MB-436 cells were treated with **B102** or **B302** for 72 h, and the cell lysates were analyzed by western blotting with the indicated antibodies. **G** MDA-MB-231 cells were treated with **B102** or **B302** for 48 h. The relative mRNA expression levels of PD-L1 were determined by RT‒PCR assay. Data are presented as the mean ± SD, unpaired Student’s *t* test, *n* = 3; *p < 0.05; **p < 0.01; ***p < 0.001. **H** MDA-MB-231 cells were treated with **B302** for 48 h, and the cell lysates were analyzed by western blotting with the indicated PD-L1 antibodies. **I** Schematic of the combination of **B102** or **B302** and anti-PD-L1 study in syngeneic murine 4T1 model. **J** The tumor growth curve was plotted as tumor volume versus time since tumor inoculation. Error bars represent the means ± SEMs, unpaired Student’s *t* test, *n* = 5; **p < 0.01; ***p < 0.001; ****p < 0.0001. **K** The tumor weight at the end of the experiments is shown. Error bars represent the means ± SEMs, unpaired Student’s *t* test, *n* = 5; *p < 0.05; **p < 0.01. **L** Schematic illustration of novel dual PARP and HDAC inhibitors stimulated signaling in breast cancer.

As previously reported, PARPi and HDACi have immunomodulatory activities, including increasing neoantigen generation and upregulating antigen-processing and presentation gene expression [22–24]. First, we assessed the effects of PARP/HDACi on the expression of the neoantigens CT45A1 and SPANXB1. The results showed upregulation of neoantigen transcription induced by PARP/HDACi treatment in TNBC cell lines and tumor-bearing mice (Fig. 6A and Fig. S3A, S4A). We also found that PARP/HDACi significantly upregulated the antigen-processing and presentation genes, such as HLA-A and HLA-B (Fig. 6B and Fig. S3B, S4B), TAP1 and TAP2 (Fig. 6C and Fig. S3C), LMP2 and LMP7 (Fig. 6D and Fig. S3D, S4C) and B2M (Fig. 6E, F and Fig. S3E, S4D).

Elevated PD-L1 expression in cancer cells might lead to the suppression of the therapeutic efficacy of PD-1 or PD-L1 antibody, both of which are approved by the FDA as an immune checkpoint blockade (ICB) therapeutic strategies to restore T-cell function [40–42]. PARPis and HDACis can increase the expression of PD-L1, but the combination of ICB with PARPi or HDACi would synergistically inhibit tumor growth and prolong survival [26, 27]. Our results showed that PARP/HDACi treatment increases PD-L1 mRNA (Fig. 6G and Fig. S3F, S4E) and protein (Fig. 6H and Fig. S3G, H) expression in three TNBC cell lines, indicating that PARP/HDACi can be combined with ICB therapy to observe the synergistic antitumor effect in the immunocompetent mouse model. The combination of PARP/HDACi and anti-PD-L1 studies showed that both PARP/HDACi and anti-PD-L1 restricted tumor growth, but the combined treatment demonstrated a better therapeutic outcome than each treatment alone (Fig. 6I–K and Fig. S5). The synergistic effect of PARP/HDACi and anti-PD-L1 ICB is a potential therapeutic approach to treat breast cancer.

## Discussion

PARPis have been approved as the treatment for many solid tumors [5, 6, 43], but some patients frequently do not respond to PARPis, and the efficacy of a single-agent PARPi exhibiting durable therapeutic effects, is still below expectations. Moreover, acquired resistance to PARPis also arises in some advanced diseases, which called for the development of new therapeutic or combination treatment strategies to sensitize or overcome resistance to PARPis. Anti-angiogenic agents and HDAC inhibitors have been reported to induce BRCAness to improve PARPi efficacy [8–10, 44]. Recent studies have revealed that epigenetic therapy can regulate cancer immunopathology and mediate antitumor immunity [45–49]. However, epigenetic modulators have limited activity in solid tumors and are mostly approved for clinical use in hematological malignancies [12, 50]. In this study, we found a positive correlation between PARP and HDAC, and duals inhibitors showed a synergistic effect in breast cancer cells (Fig. 1). We designed and synthesized another series of novel benzamide derivatives of olaparib as dual PARP and HDAC inhibitors, some of which exert PARP and HDAC enzyme inhibitory activities and exhibit antitumor effects in vitro and in vivo. PARP/HDACi led to a reduction in the functional defects in HRR to restore synthetic lethality, which might be due to the downregulation of the expression of critical components of the HRR genes RAD51 and BRCA1 (Fig. 3C–E and Fig. 4G, H). These synergistic effects of PARP/HDACi addressed the previous issue that PARP inhibitors could only be used for BRCA-mutated cells and avoided the acquired resistance to PARP inhibitors used alone.

Clinical trials and recent studies suggested that patients with the mismatch repair (MMR)-deficient or microsatellite instability (MSI)­-high (MSI-H) phenotype exhibit a higher TMB level, increasingly activated CD8^+^ cytotoxic T-cell infiltration and upregulation of inhibitory immune receptors CTLA-4, PD-1 and/or PD-L1, making these patients susceptible to ICB therapy as a possible curative treatment [51–55]. However, ICB therapies do not work for MMR-proficient cancers, which harbor fewer somatic mutations and high genomic stability. Without inherent genomic instability, chemotherapy can also induce or amplify genomic instability and promote aberrant cytosolic nucleic acids, such as double-stranded DNA (dsDNA) and double-stranded RNA (dsRNA), which can be recognized by pattern recognition receptors (PRRs), including cGAS and RIG-I, respectively, and then activate the cGAS–STING DNA-sensing pathway and RIG-I/MDA5–MAVS RNA-sensing pathway [36, 56]. Therefore, we hypothesized that inhibitors could target DNA damage repair or induce tumor-specific mutations to enhance neoantigen generation, which could improve the effect of immunotherapy and promote an antitumor immune response. Here, we designed PARP/HDACis and demonstrated that PARP/HDACis can induce DNA damage and cytosolic nucleic acid formation (Fig. 3A, B) followed by activation of the cGAS–STING–TBK1–IRF3 signaling pathway (Fig. 5A–D), upregulation of IFNβ gene expression (Fig. 5E), and subsequent activation of the JAK–STAT pathway (Fig. 5F), inducing the secretion of the chemokines CCL5 and CXCL10 (Fig. 5G, H and Fig. S2). Further studies will be required to determine the precise antitumor effects of PARP/HDACi on T-cell infiltration and T-cell subsets in vivo.

Recent findings have shown that PD-L1 overexpression on cancer cells evades antitumor immunity, but upregulated PD-L1 improves ICB therapy efficacy in a mouse model [57, 58] and sensitizes immunotherapy-resistant tumors to ICB therapy [59]. PARP and HDAC inhibitors upregulate PD-L1 [26, 27], and their combination with anti-PD-1 or anti-PD-L1 ICB shows synergistic effects [17–20]. Moreover, PARPi and HDACi potentiate immunomodulatory activities with an increase in neoantigens and upregulation of antigen-processing and presentation genes [22–24]. In this study, our results showed increases in PD-L1 expression on cancer cells (Fig. 6G, H and Fig. S3F–H, S4E), induction of neoantigen expression (Fig. 6A and Fig. S3A, S4A), upregulation of MHC and antigen-processing genes (Fig. 6B–F and Fig. S3B–E, S4B–D), and rendering immunologically cold breast cancer higher sensitivity to ICB (Fig. 6I–K and Fig. S5).

In summary, we designed and synthesized novel dual PARP and HDAC inhibitors that induce BRCAness to restore synthetic lethality, activating tumoral IFN signaling via the cGAS–STING pathway, followed by the induction cytokine production and the promotion of neoantigen generation and presentation to enhance the immune response (Fig. 6L).

## Materials and methods

### Cells and cell culture

The TNBC cell lines MDA-MB-231, MDA-MB-436, MDA-MB-468, and 4T1, the breast cancer cell line MCF-7, and the breast cell lines MCF-10A were kindly provided by Cell Bank, Chinese Academy of Sciences. All cell lines were cultured according to the supplier’s instructions, characterized by short tandem repeat (STR) profiling, and there was no mycoplasma contamination.

### Western blot analysis

Western blot analysis was performed as previously described [60], and all western blot analyses were performed at least three times.

### Cell viability assay

The viability assay was measured by a 3-(4,5-dimethylthiazol-2-yl)−2,5-diphenyltetrazolium bromide (MTT) assay.

### Apoptosis assay

The effect of PARP/HDACi treatment on apoptosis was measured by an Annexin V-FITC/PI dual staining assay.

### Cell migration and invasion assay

Cell migration and invasion assays were performed using Transwell chambers with 8 μm pores without Matrigel (for migration assay) or with Matrigel (for invasion assay).

### Animal study

MDA-MB-436, MDA-MB-231, and 4T1 cells were orthotopically injected into the fat pads of 6- to 7-week-old female NOD-Prkdc^scid^ Il2rg^em1Smoc^ (M-NSG), BALB/c nude and BALB/c mice, respectively. Fourteen 6-week-old female MMTV-PyMT mice were divided into two groups. The two mice in each group were littermates. All animal procedures were approved by the Institutional Animal Care and Use Committee at Shenzhen University.

Details about the compound design and chemical synthesis, enzymatic inhibition assays, cell culture, western blot analysis, cell viability assay, colony formation assay, apoptosis assay, cell migration, and invasion assay, wound healing assay, quantitative real-time PCR, immunofluorescence analyses, animal study, database analysis, and statistical analysis are described in the Supplemental Information. All these analyses were performed at least three times.

### Supplementary information

Revised Supporting Information

Original Data File

Checklist

## Data Availability

The data that support the findings of this study are available from the corresponding author upon request.
