# Supplementary material for: Novel dual inhibitors of PARP and HDAC induce intratumoral STING-mediated antitumor immunity in triple-negative breast cancer
Source: Cell Death Dis. 2024 Jan 5;15(1):10. doi: 10.1038/s41419-023-06303-z (PMC10770036; doi:10.1038/s41419-023-06303-z)
Supplement: Supplementary file 1 — Revised Supporting Information [file 41419_2023_6303_MOESM1_ESM.docx]

**Supporting Information**

**Novel Dual Inhibitors of PARP and HDAC Induce Intratumoral STING-Mediated Antitumor Immunity in Triple-Negative Breast Cancer**

# Figs. S1 to S5


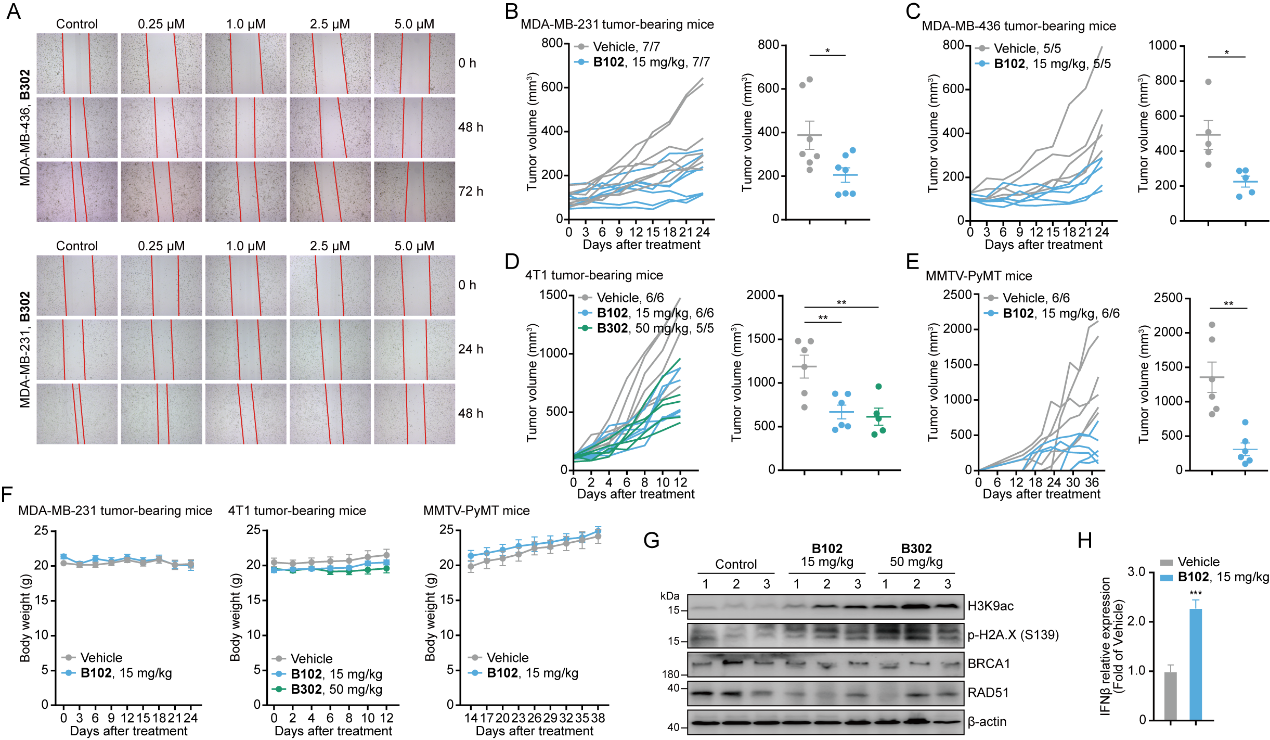


**Figure S1.** Dual PARP and HDAC inhibitors exert an antitumor effect *in vitro* and *in vivo*. (A) Wound healing assay of **B302** at different concentrations in MDA-MB-436 and MDA-MB-231 cells. (B‒E) The tumor growth curve and tumor size at the end of the experiments is shown. Error bars represent the means ± SEMs, unpaired Student’s *t* test, n ≥ 5; *, p < 0.05; **, p < 0.01. (F) The body weights of tumor-bearing mice were not obviously altered after treatment with **B102** (15 mg/kg/d) or **B302** (50 mg/kg/d). Error bars represent the means ± SEMs, unpaired Student’s *t* test, n ≥ 5. (G) 4T1 tumor-bearing mouse tissue lysates were analyzed by western blotting with the indicated antibodies. n = 3. (H) The relative mRNA expression levels of IFNβ were determined by RT‒PCR assay in MDA-MB-436 tumor-bearing mice. Data are presented as the mean ± SD, unpaired Student’s *t* test, n = 3; ***, p < 0.001.


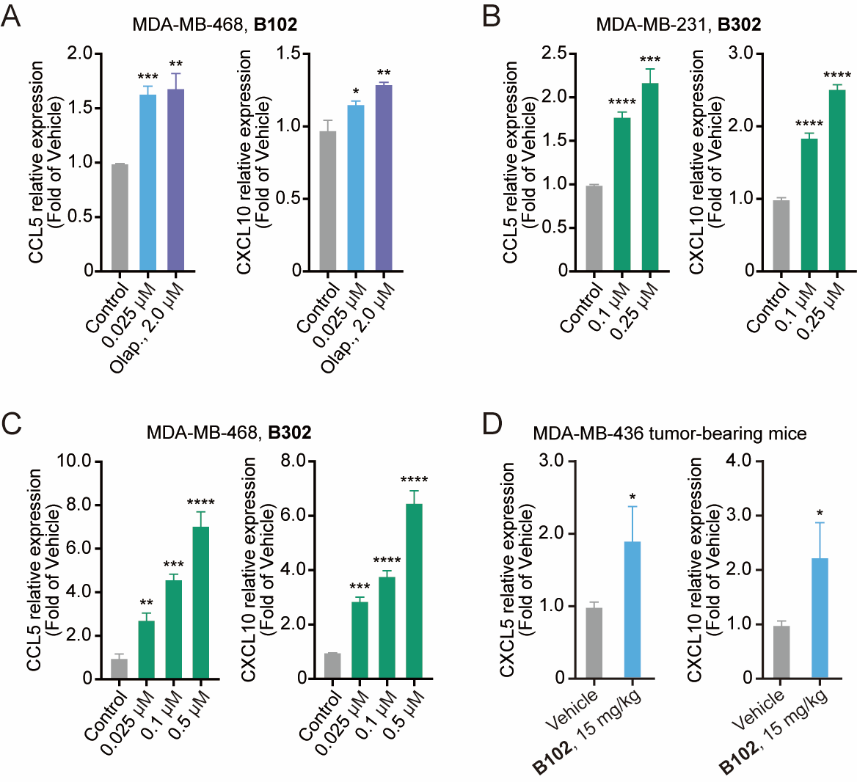


**Figure S2.** Dual PARP and HDAC inhibitors restore type I IFN signaling via the cGAS–STING pathway, leading to the production of proinflammatory chemokines. (A) MDA-MB-468 cells were treated with **B102** or olaparib for 48 h. The relative mRNA expression levels of CCL5 and CXCL10 were determined by RT‒PCR assay. Olap., olaparib. (B–C) MDA-MB-231 and MDA-MB-468 cells were treated with **B302** at different concentrations for 48 h. The relative mRNA expression levels of CCL5 and CXCL10 were determined by RT‒PCR assay. Data are presented as the mean ± SD, unpaired Student’s *t* test, n = 3; *, p < 0.05; **, p < 0.01; ***, p < 0.001; ****, p < 0.0001. (D) The relative mRNA expression levels of CCL5 and CXCL10 were determined by RT‒PCR assay in MDA-MB-436 tumor-bearing mice. Data are presented as the mean ± SD, unpaired Student’s *t* test, n = 3; *, p < 0.05.


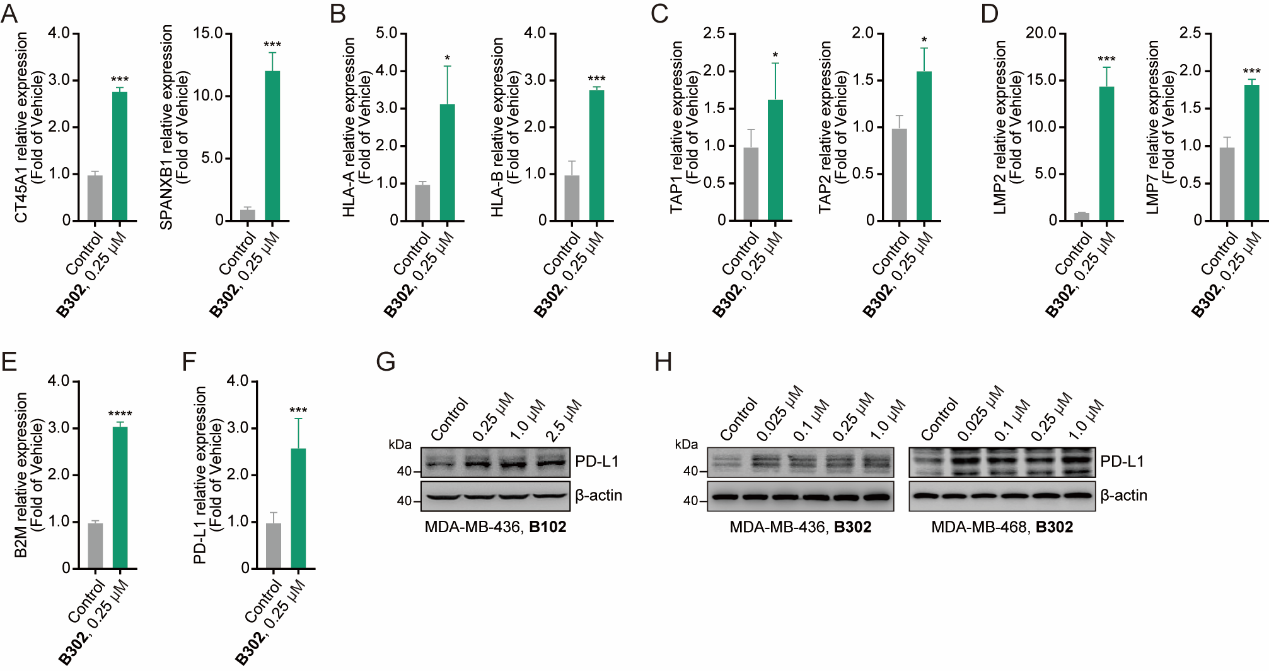


**Figure S3.** Dual PARP and HDAC inhibitors promote neoantigen generation and upregulate antigen-processing and presentation genes and PD-L1. (A) MDA-MB-436 cells were treated with **B302** for 48 h. The relative mRNA expression levels of neoantigen were determined by RT‒PCR assay. (B–E) MDA-MB-436 cells were treated with **B302** for 48 h. The relative mRNA expression levels of antigen-processing and presentation genes were determined by RT‒PCR assay. (F) MDA-MB-436 cells were treated with **B302** for 48 h. The relative mRNA expression levels of PD-L1 were determined by RT‒PCR assay. (G) MDA-MB-436 cells were treated with **B102** at different concentrations for 48 h, and the cell lysates were analyzed by western blotting with the indicated PD-L1 antibodies. (H) MDA-MB-436 and MDA-MB-468 cells were treated with **B302** at different concentrations for 48 h, and the cell lysates were analyzed by western blotting with the indicated PD-L1 antibodies. Data are presented as the mean ± SD, unpaired Student’s *t* test, n = 3; *, p < 0.05; ***, p < 0.001; ****, p < 0.0001.


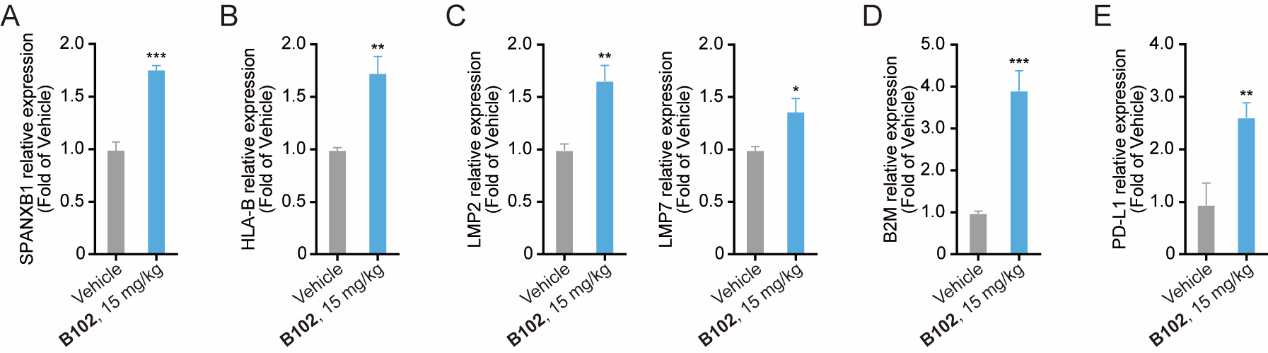


**Figure S4.** Dual PARP and HDAC inhibitors promote neoantigen generation and upregulate antigen-processing and presentation genes and PD-L1 in MDA-MB-436 tumor-bearing mice. (A) The relative mRNA expression levels of SPANXB1 were determined by RT‒PCR assay. (B) The relative mRNA expression levels of HLA-B were determined by RT‒PCR assay. (C) The relative mRNA expression levels of LAMP2 and LAMP7 were determined by RT‒PCR assay. (D) The relative mRNA expression levels of B2M were determined by RT‒PCR assay. (E) The relative mRNA expression levels of PD-L1 were determined by RT‒PCR assay. Data are presented as the mean ± SD, unpaired Student’s *t* test, n = 3; *, p < 0.05; **, p < 0.01; ***, p < 0.001.


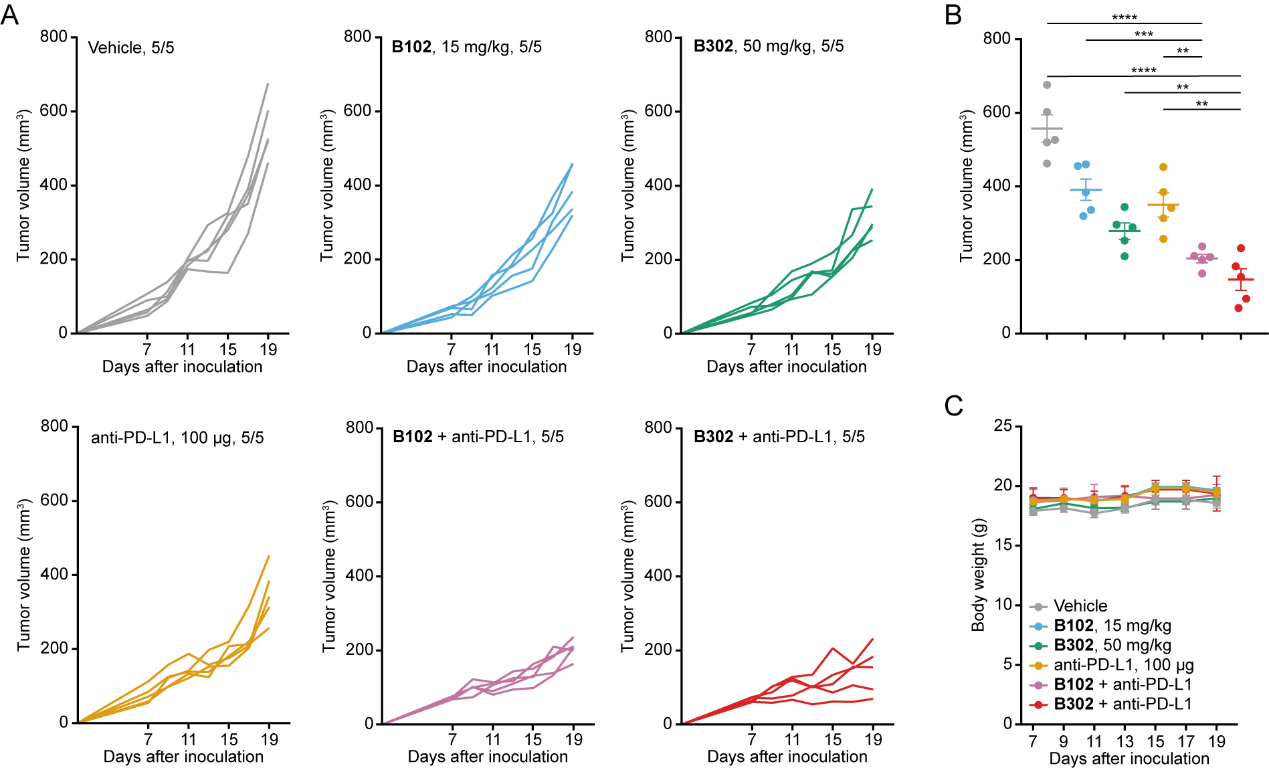


**Figure S5.** The combination of PARP/HDACi and anti-PD-L1 exert a synergistic antitumor effect *in vivo*. (A) The tumor growth curve was plotted as tumor volume versus time since tumor inoculation. (B) The tumor size at the end of the experiments is shown. Error bars represent the means ± SEMs, unpaired Student’s *t* test, n = 5; **, p < 0.01; ***, p < 0.001; ****, p < 0.0001. (C) The body weights of tumor-bearing mice were not obviously altered.

# Materials and methods

## PARP and HDAC enzymatic inhibition assays

The PARP1 and PARP2 inhibitory activities of the synthesized compounds **B101**–**B602** were evaluated by using the PARP1 Colorimetric Assay Kit (BPS Bioscience, San Diego, CA, USA, catalog number 80580) and PARP2 Colorimetric Assay Kit (BPS Bioscience, catalog number 80581), respectively, according to the manufacturer’s protocol. Briefly, the 1× histone mixture was added to each well and incubated overnight at 4 °C. Each well was washed three times with 200 µL 1× TBST buffer and filled with 200 µL Blocking buffer at room temperature for 90 min. Then, each well was filled with 25 µL of PARP master mixture (5 μL activated DNA + 2.5 μL 10× PARP Assay mixture + 2.5 μL 10× PARP buffer + 15 μL distilled water). Next, 5 µL of the compound of different concentrations was added to each well, and PARP enzyme was added and incubated at room temperature for 1 h. Next, streptavidin HRP solution was added and incubated at room temperature for 30 min. After adding 100 µL of colorimetric HRP substrate to each well, the plate was incubated until a blue color developed in the positive well, which contained PARP enzyme without inhibitors. The reaction was then quenched with 2 M sulfuric acid, and absorbance at 450 nm was measured using a microplate reader (BioTek, Winooski, VT, USA). After calculating the inhibition rate, the IC_50_ value was determined.

The *in vitro* HDAC inhibition assays were carried out by Sundia MediTech Co. Ltd. in Shanghai, China. The general procedures were as follows: each compound at various concentrations was incubated with recombinant HDAC1 (Active Motif, Carlsbad, CA, USA) and HDAC6 (BPS Bioscience) at room temperature for 15 min, and the enzyme activity was then measured by adding a mixture of Ac-peptide-AMC substrates to initiate the coupled reaction in Tris-based assay buffer and incubating at room temperature for 60 min. Reactions were stopped with trypsin, and the samples were incubated for another 90 min at 37 °C. The release of AMC was monitored by measuring the fluorescence at excitation = 355 nm and emission = 460 nm using a SynergyMx (BioTek). IC_50_ values were obtained using GraphPad Prism software.

## Cells and cell culture

The TNBC cell lines MDA-MB-231, MDA-MB-436, MDA-MB-468 and 4T1, the breast cancer cell line MCF-7, and the breast cell lines MCF-10A were kindly provided by Cell Bank, Chinese Academy of Sciences. MDA-MB-231, MDA-MB-436 and MDA-MB-468 cells were cultured in Leibovitz’s L-15 (Procell, Wuhan, China) supplemented with 10% fetal bovine serum (FBS, Gibco, Grand Island, NY, USA) and 1% penicillin/streptomycin (Gibco) at 37 °C without 5% CO_2_. 4T1 and MCF-10A cells were cultured in Roswell Park Memorial Institute (RPMI) 1640 Medium (Corning, Tewksbury, MA, USA) supplemented with 10% FBS and 1% penicillin/streptomycin and incubated at 37 °C with 5% CO_2_. MCF-7 cells were cultured in Dulbecco’s modified Eagle’s medium (DMEM, Corning) supplemented with 10% FBS and 1% penicillin/streptomycin and incubated at 37 °C with 5% CO_2_. All cell lines were characterized by short tandem repeat (STR) profiling, and there was no mycoplasma contamination.

## Western blot analysis

Western blot analysis was performed as previously described [1]. Briefly, whole cell or tissue lysates were prepared using ice-cold Nonidet P-40 buffer mixed with protease and phosphatase inhibitors (Roche, IN, USA) and centrifuged at 14,000 × *g* at 4 °C for 10 min. The protein lysates were fractionated by sodium dodecyl sulfate–polyacrylamide gel electrophoresis (SDS–PAGE) and transferred to polyvinylidene fluoride (PVDF) membranes (Merck, Darmstadt, Germany). Membranes were incubated in 1× TBST buffer containing 5% nonfat powdered milk at room temperature for 2 h. The indicated primary antibody was incubated with PVDF membranes, followed by a washing step using 1× TBST and a secondary antibody conjugated to HRP. Signal detection was carried out using the Western ECL Substrate kit (Bio-Rad, Hercules, CA, USA). The following antibodies were used for immunoblotting: phospho-histone H2A.X (Ser139) (20E3) #9718, Acetyl-Histone H3 (Lys9) (C5B11) #9649, Histone H3 (D1H2) XP #4499, BRCA1 #9010, Cleaved PARP (Asp214) #9541, Cleaved Caspase-8 (Asp391) (18C8) #9496, Cleaved Caspase-7 (Asp198) #9491, Cleaved Caspase-3 (Asp175) (5A1E) #9664, cGAS (D1D3G) #15102, Phospho-STING (Ser366) (E9A9K) #50907, STING (D2P2F) #13647, Phospho-TBK1/NAK (Ser172) (D52C2) #5483, TBK1/NAK (D1B4) #3504, Phospho-IRF-3 (Ser396) (D6O1M) #29047, IRF-3 (D6I4C) #11904, Phospho-STAT1 (Tyr701) (58D6) #9167, STAT1 (D1K9Y) #14994, PD-L1 (E1L3N) #13684, purchased from Cell Signaling Technology (Danvers, MA, USA); RAD51 (abs100449) purchased from Absin (Shanghai, China); HLA-A/B/C (sc-52810), LMP2 (sc-373996), LMP7 (sc-365699), B2M (sc-13565), purchased from Santa Cruz (Dallas, TX, USA); β-actin (AA128) was purchased from Beyotime Biotechnology (Shanghai, China). All western blot analyses were performed at least three times.

## Cell viability assay

The viability of MDA-MB-231, MDA-MB-436, MDA-MB-468, MCF-7 and MCF-10A cells was measured by a 3-(4,5-dimethylthiazol-2-yl)-2,5-diphenyltetrazolium bromide (MTT) assay (Sangon Biotech, Shanghai, China). In brief, cells were seeded at a density of 6,000 cells in 96-well plates and cultured overnight. Then, the cells were treated with different concentrations of PARP/HDACi, PARPi olaparib (Selleck, Houston, TX, USA) and HDACi chidamide (Selleck) for 72 h. Ten microliters of MTT (5 mg/mL) was added to each well and incubated for 4 h in the dark. After aspiration of the medium, the formazan crystals were dissolved in 100 μL of DMSO. The absorbance at 490 nm was measured using a microplate reader (BioTek). All these analyses were performed at least three times.

## Colony formation assay

MDA-MB-231, MDA-MB-436 and MDA-MB-468 cells were seeded at a density of 2,000 cells in 6-well plates and cultured overnight. Then, the cells were treated with different concentrations of PARP/HDACi, PARPi olaparib and HDACi chidamide and further cultured for approximately 14 days. The cells were fixed with 4% paraformaldehyde (Merck) for 1 h at room temperature and stained with 0.1% crystal violet (Beyotime Biotechnology) for 30 min. Under an inverted phase contrast microscope (Leica Microsystems, Heidelberg, Germany), cell colonies were photographed at 5× magnification. All these analyses were performed at least three times.

## Apoptosis assay

The effect of PARP/HDACi treatment on apoptosis was measured by an Annexin V-FITC/PI dual staining assay. MDA-MB-436 cells were seeded in 6-well plates and cultured overnight. Then, the cells were treated with different concentrations of PARP/HDACi for 48 h. The cells were digested and washed with PBS buffer. Then, the collected cells were resuspended in 500 μL of binding buffer and stained with 5 μL of Annexin V-FITC and 5 μL of PI for 15 min in the dark using an Annexin V-FITC/PI Apoptosis Detection Kit (Yeason, Shanghai, China). The proportion of apoptotic cells was analyzed by BD FACSCalibur flow cytometry (BD Biosciences, San Jose, CA, USA).

## Cell migration and invasion assay

Cell migration and invasion assays were performed using Transwell chambers with 8 μm pores (Corning) without Matrigel (for migration assay) or with Matrigel (for invasion assay). MDA-MB-436 and MDA-MB-231 cells were resuspended in serum-free Leibovitz’s L-15 medium and seeded in the upper chamber at a density of 12,000 cells/100 μL. The chambers were incubated in 24-well plates containing Leibovitz’s L-15 medium with 10% FBS and PARP/HDACi or DMSO. After 48 h, the cells were fixed with 4% paraformaldehyde and stained with crystal violet. The cells on the upper surface of the filters were removed by swabbing with a cotton swab, and the images were captured at 5× magnification under an inverted phase contrast microscope (Leica Microsystems).

## Wound healing assay

MDA-MB-231 and MDA-MB-436 cells were seeded at a density of 1 × 10^6^ cells in 6-well plates. After 24 h, the cells reached confluence. A 200 μL pipet tip was used to scoop a physical gap, and the plates were washed with PBS to remove cell debris. Then, the cells were treated with different concentrations of PARP/HDACi or DMSO as a control. Consequently, the width of the wound was captured under an inverted phase contrast microscope (Leica Microsystems) at 0, 24, 48 and 72 h time points.

## Quantitative real-time PCR

Total RNA was extracted using the Eastep Super Total RNA Extraction Kit (Promega, Charbonnières, France) according to the manufacturer’s protocol. Reverse transcription was performed using HyperScript RT SuperMix for qPCR (APExBIO, Houston, TX, USA). Gene expression was quantified with Hieff qPCR SYBR Green Master Mix (Yeason) on an ABI 7500 (Applied Biosystems, Grand Island, NY, USA). Data were then analyzed using the 2^-ΔΔCT^ method. The primers were designed and synthesized by Sangon Biotech, and the sequences are shown in Supporting Information Table S1.

## Immunofluorescence analyses

MDA-MB-231, MDA-MB-436 and MDA-MB-468 cells were added to 6-well culture plates with three glass slides and incubated at 37 °C for 24 h. Cells were then treated with DMSO or different concentrations of PARP/HDACi for 48 h. Cells were fixed with 4% paraformaldehyde and permeabilized with 0.2% Triton X-100 in PBS. Following PBS wash, glass slides were blocked and permeabilized with 5% normal goat serum for 30 min and incubated with antibody against p-H2A.X (Ser139) (CST, 9718), dsDNA (Merck, MAB1293) overnight at 4 °C. Then, the cells were incubated with corresponding FITC- or Cy3-conjugated secondary antibodies (Proteintech, Wuhan, China) followed by nuclear staining with DAPI (Thermo Fisher Scientific, Waltham, MA, USA). Immunofluorescence images were acquired on a ZEISS laser scanning confocal microscope (LSM 800, ZEISS, Jena, Germany).

## Animal study

MDA-MB-436 cells (5 × 10^6^) were suspended in PBS and Matrigel (Corning) at a 1:1 ratio and orthotopically injected into the fat pads of 6- to 7-week-old female NOD-Prkdc^scid^ Il2rg^em1Smoc^ (M-NSG) mice from the Shanghai Model Organisms Center, Inc. MDA-MB-231 cells (5 × 10^6^) were suspended in PBS orthotopically injected into the fat pads of 6- to 7-week-old female BALB/c nude mice from the Shanghai Model Organisms Center, Inc. 4T1 cells (1 × 10^6^) were suspended in PBS and injected into the fat pads of 6- to 7-week-old male BALB/c mice from GemPharmatech Co., Ltd. When the tumor size reached 100 mm^3^, the mice were randomly divided into three treatment groups without blinding: **B102** (15 mg/kg), **B302** (50 mg/kg) or vehicle [10% N,N-dimethylacetamide (DMAC, Sangon Biotech) and 6% polyethylene glycol 12-hydroxystearate (Solutol HS-15, MedChem Express, Monmouth Junction, NJ, USA) in saline] treatment via an intraperitoneal injection once daily. For the MMTV-PyMT mice, fourteen 6-week-old female mice were randomly divided into two groups, and no blinding was performed. Each group of mice was treated with **B102** (15 mg/kg) or vehicle via an intraperitoneal injection once daily for 38 days. For the combination of **B102** or **B302** and anti-PD-L1 studies, the mice were randomly divided into four treatment groups without blinding: (1) vehicle; (2) **B102** (15 mg/kg, every 2 days) or **B302** (50 mg/kg, every 2 days); (3) anti-PD-L1 antibodies (100 μg, dissolved in PBS, every 5 days, Atezolizumab, Clone No.: 2B11D11, Shanghai Lollane biological technology Co., Ltd., Shanghai, China); or (4) combination of **B102** or **B302** and anti-PD-L1, all treatment via an intraperitoneal injection. Tumor size was monitored with a digital caliper, and tumor volume was calculated using the following equation: volume = (length × width^2^)/2. At the end of treatment, the mice were euthanized, and tumor tissues were harvested and weighed. All animal procedures were approved by the Institutional Animal Care and Use Committee at the Shenzhen University.

## Database analysis and code availability

Gene expression data were retrieved from the UCSC TOIL RNA-seq recompute project [2] via the UCSC Xena database (https://xenabrowser.net/datapages/). Clinical information of samples in the TCGA-BRCA project [3] was retrieved from the TCGA Hub via UCSC Xena. Kaplan–Meier overall survival curves of human breast tumors generated using Kaplan–Meier Plotter according to PARP1 and HDAC1 gene [4] or protein [5] expression levels, with auto select best cut-off selected. The bioinformatics analyses in this study were performed using R 4.2.3 and R package survival (version 3.5-5), and the R package ggplot2 (version 3.4.2) was used to plot gene difference analysis, correlation analysis and Kaplan–Meier survival curves.

## Statistical analysis

All experiments that required statistical analysis were performed independently with at least three replications. The experimental data are expressed as the mean value ± standard deviation (SD) or standard error of the mean (SEM). The statistical analysis was conducted using GraphPad Prism 8.0. The significance of differences was tested using an unpaired Student’s *t* test for the comparison of two samples and one-way analysis of variance (ANOVA) for the comparison of multiple samples. A statistically significant difference was defined as a difference with a p value < 0.05. Statistical computations were performed using R 4.2.0. Group-wise comparison of different gene expression levels was performed using a 2-tailed Mann‒Whitney U test. Correlation analyses between HDAC1 and PARP1 expression levels were performed according to the Pearson test. Mantel–Cox log-rank test was used for Kaplan–Meier survival curves. Inclusion/exclusion criteria were all pre-established and no samples or animals were excluded from the analysis.

# Sequences of oligonucleotides

**Table S1.** Primers sequences

| **Gene** | **Forward Primer (5’-3’)** | **Reverse Primer (5’-3’)** |
| --- | --- | --- |
| BRCA1 | TGAGGCATCAGTCTGAAAGTTCTGGA | CTGATGTGCTTTGTTCTGGA |
| RAD51 | TGCTTATTGTAGACAGTGCCACC | CACCAAACTCATCAGCGAGTC |
| CCL5 | CCAGCAGTCGTCTTTGTCAC | CTCTGGGTTGGCACACACTT |
| CXCL10 | GTGGCATTCAAGGAGTACCTC | TGATGGCCTTCGATTCTGGATT |
| IFNβ | AACTTGCTTGGATTCCTACAAAG | TATTCAAGCCTCCCATTCAATTG |
| PD-L1 | TCACTTGGTAATTCTGGGAGC | CTTTGAGTTTGTATCTTGGATGCC |
| CT45A1 | GCACCTGTGGGAGGAAACG | CCTGACTGCAGTAGGTCCTTG |
| SPANXB1 | CCTGACTGCAGTAGGTCCTTG | CCACTAGTATGGTCGAGGACTC |
| HLA-A | TCAGATAGAAAAGGAGGGAGTTACA | ACAAGCTGTGAGGGACACAT |
| HLA-B | CCTGAGATGGGAGCCGTCTT | CTCCGATGACCACAACTGCT |
| TAP1 | CTCTGGAAACCCTGTGCGT | GACCTTCCACTAGACCATGAGC |
| TAP2 | GACCTTCCACTAGACCATGAGC | GGAGGATTAAGATTAGTACGATGGT |
| LMP2 | CTATCCAGCGTACTCCAAAG | GAAAGACCAGTCCTTGCTGA |
| LMP7 | GGTCCTACATTAGTGCCTTACGG | CGCAGATAGTACAGCCTGCATT |
| B2M | AGACCTTTGGGCTGCCTTAT | TAGCCTCCCTCACTCCAAGA |
| GAPDH | CAAGGTCATCCATGACAACTTTG | GTCCACCACCCTGTTGCTGTAG |

# Chemistry

All reagents and solvents were analytically pure and obtained from commercial sources, and no further purification was needed. All reactions were conducted under an atmosphere of nitrogen. Nuclear magnetic resonance (NMR) spectra were acquired on a Bruker 400 (400 MHz ^1^H, 100 MHz ^13^C) spectrometer. Chemical shifts were reported in ppm (δ) using tetramethylsilane (TMS) as an internal standard and CDCl_3_ or DMSO-*d*_6_ as solvents. Multiplicities are abbreviated as follows: s = singlet, d = doublet, t = triplet, m = multiplet, br s = broad singlet. Coupling constant (J) values are given in hertz (Hz). The mass spectra were recorded on a Waters Micromass Q-TOF Premier Mass Spectrometer. Melting points were determined using a Hanon MP430 Melting Point Apparatus. Silica gel thin-layer chromatography was performed on precoated F254 plates. No effort was put into yield optimization and generally represented the result of a single experiment. Compounds **B11** and **B102** were synthesized by following the procedures reported in the previous literature [6], as well as compound **B101**–**B602** following reported protocols.

# General procedure for compounds B101–B602

Compound **1** (400 mg, 1.09 mmol, 1 eq) was dissolved in acetonitrile (10 mL), and then Methyl 3-(4-bromomethyl)cinnamate (1 eq) and DIEA (2 eq) were added. The mixture was stirred at room temperature for 2 h, and then the precipitate was filtered, washed with water, acetonitrile and ethyl ether, and dried to give product **B1**.

LiOH (4 eq) was added to a stirring solution of **B1** (1 eq) in a THF/water mix (3:1, 10 mL). The resultant mixture was stirred at room temperature overnight. The resultant aqueous solution was tapped up with water (10 mL) and then acidified to pH 1 using aqueous 2 M HCl. Then the resulting mixture was extracted with EtOAc. The organic layer was washed with brine, dried over Na_2_SO_4_, filtered, and concentrated in vacuo to give **B10** as white solid.

Compound **B10** (1 eq) was dissolved in DMF (10 mL), and 1,2-phenylenediamine or 4-Fluoro-1,2-phenylenediamine (1 eq), HATU (2 eq) and DIEA (2 eq) were added. The mixture was stirred at room temperature for 2 h, and then the resulting mixture was tapped up with water, filtered. The solid was washed with ethyl ether and purified by flash column chromatography (DCM/MeOH, 10:1 *V*/*V*) to yield **B101**–**B602**, which were then characterized by ^1^H NMR, ^13^C NMR, melting point and high resolution mass spectrum (HRMS, ESI).

(E)-N-(2-aminophenyl)-3-(4-((4-(2-fluoro-5-((4-oxo-3,4-dihydrophthalazin-1-yl)methyl)benzoyl)piperazin-1-yl)methyl)phenyl)acrylamide(**B101**); yellow solid; yield 70%; mp 140–141 °C; ^1^H NMR (400 MHz, DMSO-*d*_6_) δ 12.59 (s, 1H), 9.38 (s, 1H), 8.24 (dd, *J* = 7.9, 1.4 Hz, 1H), 7.94 (d, *J* = 8.0 Hz, 1H), 7.90–7.76 (m, 2H), 7.60–7.49 (m, 3H), 7.44–7.27 (m, 5H), 7.20 (t, *J* = 9.0 Hz, 1H), 6.93–6.83 (m, 2H), 6.73 (dd, *J* = 8.0, 1.4 Hz, 1H), 6.60–6.53 (m, 1H), 4.94 (s, 2H), 4.31 (s, 2H), 3.60 (s, 2H), 3.49 (s, 2H), 3.15 (d, *J* = 5.9 Hz, 2H), 2.39 (s, 2H), 2.24 (s, 2H). ^13^C NMR δ 164.22, 164.01, 159.86, 156.83 (d, *J* = 244.6 Hz), 145.36, 142.07, 140.05, 139.86, 135.31, 135.28, 134.25, 133.97, 132.08, 132.04, 132.00, 129.96, 129.56, 129.24, 129.20, 128.40, 128.08, 126.57, 126.26, 125.97, 125.19, 124.41, 124.22, 124.03, 122.47, 116.79, 116.51, 116.28, 61.90, 53.19, 52.68, 47.07, 41.86, 36.91. HRMS (ESI) m/z calculated for [M-H]**^-^** 617.26709, found 617.26727.

1. -N-(2-amino-4-fluorophenyl)-3-(4-((4-(2-fluoro-5-((4-oxo-3,4-dihydrophthalazin-1-yl)methyl)benzoyl)piperazin-1-yl)methyl)phenyl)acrylamide(**B102**). brown solid; yield 65%; mp 139-140 °C; ^1^H NMR (400 MHz, DMSO-*d*_6_) δ 12.59 (s, 1H), 9.32 (s, 1H), 8.24 (dd, *J* = 7.8, 1.5 Hz, 1H), 7.94 (d, *J* = 8.1 Hz, 1H), 7.89–7.76 (m, 2H), 7.61–7.48 (m, 3H), 7.37 (dd, *J* = 15.4, 6.8 Hz, 3H), 7.31–7.16 (m, 3H), 6.82 (d, *J* = 15.8 Hz, 1H), 6.50 (dd, *J* = 11.2, 2.9 Hz, 1H), 6.33 (d, *J* = 2.9 Hz, 1H), 5.25 (s, 2H), 4.30 (s, 2H), 3.60 (s, 2H), 3.49 (s, 2H), 3.14 (s, 2H), 2.39 (s, 2H), 2.24 (s, 2H). ^13^C NMR (100 MHz, DMSO-*d*_6_) δ 164.26, 159.88, 155.63, 145.38, 140.06, 139.91, 135.30, 134.23, 133.97, 132.04, 129.96, 129.57, 129.22, 128.40, 128.07, 127.04, 126.58, 125.95, 124.42, 124.23, 122.35, 116.50, 116.28, 102.71, 102.49, 102.17, 61.89, 53.17, 52.66, 47.07, 41.88, 36.92. HRMS (ESI) m/z calculated for [M-H]**^-^** 635.25767, found 635.25793.

N-(2-aminophenyl)-4-((4-(2-fluoro-5-((4-oxo-3,4-dihydrophthalazin-1-yl)methyl)benzoyl)piperazin-1-yl)methyl)benzamide (**B201**); white solid; yield 68%; mp 157–158 °C; ^1^H NMR (400 MHz, DMSO-*d*_6_) δ 12.60 (s, 1H), 9.65 (s, 1H), 8.25 (d, *J* = 7.8 Hz, 1H), 8.03–7.74 (m, 5H), 7.42 (d, *J* = 7.9 Hz, 3H), 7.30 (d, *J* = 6.3 Hz, 1H), 7.25–7.09 (m, 2H), 6.95 (t, *J* = 7.7 Hz, 1H), 6.77 (d, *J* = 8.0 Hz, 1H), 6.58 (t, *J* = 7.4 Hz, 1H), 4.89 (s, 2H), 4.31 (s, 2H), 3.58 (d, *J* = 26.6 Hz, 4H), 3.19–3.14 (m, 2H), 2.40 (s, 2H), 2.26 (s, 2H). ^13^C NMR (100 MHz, DMSO-*d*_6_) δ 165.65, 164.26, 159.88, 156.85 (d, *J* = 244.5 Hz), 145.36, 143.59, 141.86, 135.32, 135.28, 133.96, 132.09, 132.03, 129.58, 129.25, 129.22, 129.18, 128.42, 128.26, 127.13, 126.94, 126.58, 125.95, 124.42, 124.24, 123.93, 116.80, 116.67, 116.50, 116.28, 61.81, 53.18, 52.64, 49.11, 47.07, 41.87, 36.93. HRMS (ESI) m/z calculated for [M+H]**^+^** 591.25144, found 591.25171.

N-(2-amino-4-fluorophenyl)-4-((4-(2-fluoro-5-((4-oxo-3,4-dihydrophthalazin-1-yl)methyl)benzoyl)piperazin-1-yl)methyl)benzamide (**B202**)，white solid; yield 59%，mp 164–165 °C; ^1^H NMR (400 MHz, DMSO-*d*_6_) δ 12.59 (s, 1H), 9.57 (s, 1H), 8.35–8.21 (m, 1H), 7.94 (dd, *J* = 8.2, 3.8 Hz, 3H), 7.88–7.77 (m, 2H), 7.41 (d, *J* = 7.9 Hz, 3H), 7.29 (dd, *J* = 6.6, 2.3 Hz, 1H), 7.20 (t, *J* = 9.0 Hz, 1H), 7.09 (dd, *J* = 8.7, 6.2 Hz, 1H), 6.52 (dd, *J* = 11.2, 2.9 Hz, 1H), 6.34 (d, *J* = 2.9 Hz, 1H), 5.22 (s, 2H), 4.31 (s, 2H), 3.57 (d, *J* = 24.5 Hz, 4H), 3.15 (d, *J* = 4.7 Hz, 2H), 2.40 (d, *J* = 5.2 Hz, 2H), 2.26 (d, *J* = 5.0 Hz, 2H). ^13^C NMR (100 MHz, DMSO-*d*_6_) δ 165.90, 164.25, 162.70, 160.32, 159.87, 158.05, 155.62, 146.00, 145.88, 145.37, 141.88, 135.32, 135.29, 133.96, 133.83, 132.04, 132.01, 129.57, 129.24, 129.21, 129.15, 129.05, 128.95, 128.41, 128.29, 126.58, 125.96, 124.41, 124.23, 119.84, 116.50, 116.29, 102.65, 102.43, 102.11, 101.86, 61.80, 53.18, 52.63, 49.10, 47.06, 41.86, 36.91. HRMS (ESI) m/z calculated for [M+H]**^+^** 609.24202, found 609.24213.

N-(2-aminophenyl)-2-(4-((4-(2-fluoro-5-((4-oxo-3,4-dihydrophthalazin-1-yl)methyl)benzoyl)piperazin-1-yl)methyl)phenyl)acetamide (**B301**)，yellow solid; yield 60%，mp 118–119 °C; ^1^H NMR (400 MHz, DMSO-*d*_6_) δ 12.59 (s, 1H), 9.35 (s, 1H), 8.24 (dd, *J* = 7.8, 1.5 Hz, 1H), 7.98 – 7.90 (m, 1H), 7.81 (dtd, *J* = 24.6, 7.3, 1.3 Hz, 2H), 7.39 (ddd, *J* = 8.2, 5.2, 2.3 Hz, 1H), 7.32–7.26 (m, 3H), 7.25–7.21 (m, 2H), 7.19–7.09 (m, 2H), 6.87 (td, *J* = 7.6, 1.6 Hz, 1H), 6.69 (dd, *J* = 8.0, 1.4 Hz, 1H), 6.56–6.45 (m, 1H), 4.82 (s, 2H), 4.30 (s, 2H), 3.62 (d, *J* = 3.9 Hz, 2H), 3.43 (s, 2H), 3.15–3.09 (m, 2H), 2.86 (s, 1H), 2.71 (s, 1H), 2.37 (t, *J* = 5.1 Hz, 2H), 2.21 (d, *J* = 5.6 Hz, 2H). ^13^C NMR (100 MHz, DMSO-*d*_6_) δ 169.64, 164.24, 162.82, 159.88, 156.84 (d, *J* = 244.5 Hz), 145.37, 142.36, 136.35, 135.58, 135.28, 135.25, 133.95, 132.03, 131.96, 129.72, 129.64, 129.56, 129.44, 129.38, 129.23, 129.19, 128.44, 128.40, 126.57, 126.37, 125.93, 125.78, 125.74, 124.43, 124.25, 123.86, 116.71, 116.49, 116.40, 116.27, 62.02, 53.16, 52.64, 49.11, 47.07, 42.90, 41.87, 36.92, 36.28, 31.29. HRMS (ESI) m/z calculated for [M+H]**^+^** 605.26709, found 605.26709.

N-(2-amino-4-fluorophenyl)-2-(4-((4-(2-fluoro-5-((4-oxo-3,4-dihydrophthalazin-1-yl)methyl)benzoyl)piperazin-1-yl)methyl)phenyl)acetamide (**B302**); brown solid; yield 69.5%，mp 119–120 °C; ^1^H NMR (400 MHz, DMSO-*d*_6_) δ 12.59 (s, 1H), 9.29 (s, 1H), 8.24 (dd, *J* = 7.9, 1.5 Hz, 1H), 7.93 (d, *J* = 8.0 Hz, 1H), 7.89–7.74 (m, 2H), 7.39 (ddd, *J* = 8.2, 5.2, 2.2 Hz, 1H), 7.31–7.26 (m, 3H), 7.25–7.18 (m, 3H), 7.07 (dd, *J* = 8.7, 6.3 Hz, 1H), 6.46 (dd, *J* = 11.2, 2.9 Hz, 1H), 6.31–6.23 (m, 1H), 5.15 (s, 2H), 4.30 (s, 2H), 3.60 (s, 4H), 3.32 (s, 1H), 3.13 (d, *J* = 8.4 Hz, 2H), 2.97 (s, 1H), 2.36 (t, *J* = 5.0 Hz, 2H), 2.22 (s, 2H). ^13^C NMR (100 MHz, DMSO-*d*_6_) δ 169.87, 164.21, 164.17, 162.23, 159.86, 158.93, 158.04, 156.85, 155.61, 145.37, 144.79, 144.67, 136.33, 135.96, 135.53, 135.29, 135.26, 133.96, 133.67, 132.07, 132.03, 131.99, 129.74, 129.56, 129.51, 129.48, 129.38, 129.25, 129.21, 128.46, 128.39, 127.76, 127.60, 127.49, 127.24, 126.57, 125.95, 124.41, 124.23, 119.85, 119.83, 116.49, 116.28, 102.55, 102.32, 101.98, 101.73, 62.01, 54.12, 53.16, 52.63, 47.05, 42.76, 42.63, 41.85, 41.19, 36.91. HRMS (ESI) m/z calculated for [M+H]**^+^** 623.25767, found 623.25793.

N-(2-aminophenyl)-2-(4-(2-fluoro-5-((4-oxo-3,4-dihydrophthalazin-1-yl)methyl)benzoyl)piperazin-1-yl)acetamide (**B401**); yellow solid; yield 62.5%; mp 121–122 °C; ^1^H NMR (400 MHz, DMSO-*d*_6_) δ 12.59 (s, 1H), 9.15 (d, *J* = 2.5 Hz, 1H), 8.24 (dd, *J* = 7.8, 1.4 Hz, 1H), 7.97–7.79 (m, 2H), 7.40 (dt, *J* = 6.0, 2.9 Hz, 1H), 7.33 (dd, *J* = 6.5, 2.3 Hz, 1H), 7.24–7.16 (m, 3H), 6.92 (ddd, *J* = 12.6, 6.7, 2.4 Hz, 2H), 6.74 (dd, *J* = 7.9, 1.5 Hz, 1H), 6.59–6.51 (m, 1H), 4.78 (s, 2H), 4.31 (s, 2H), 3.67 (s, 2H), 3.15 (s, 2H), 2.99 (s, 2H), 2.59 (d, *J* = 5.3 Hz, 2H), 2.44 (s, 2H). ^19^F NMR (376 MHz, DMSO-*d*_6_) δ -119.86. ^13^C NMR (100 MHz, DMSO-*d*_6_) δ 168.43, 164.30, 164.25, 159.88, 158.93, 158.04, 156.88, 156.83 (d, *J* = 244.3 Hz), 156.21, 155.61, 145.39, 142.20, 135.99, 135.34, 135.31, 133.99, 133.70, 132.06, 132.00, 129.57, 129.38, 129.29, 129.25, 128.40, 127.90, 127.24, 126.57, 126.36, 126.32, 125.96, 125.44, 124.42, 124.24, 124.13, 124.09, 120.47, 117.15, 116.83, 116.49, 116.27, 112.07, 61.48, 53.26, 52.83, 47.06, 42.64, 41.85, 41.20, 38.65, 36.92, 29.49. HRMS (ESI) m/z calculated for [M+H]**^+^** 515.22014, found 515.22052.

N-(2-amino-4-fluorophenyl)-2-(4-(2-fluoro-5-((4-oxo-3,4-dihydrophthalazin-1-yl)methyl)benzoyl)piperazin-1-yl)acetamide (**B402**); brown solid; yield 78%; mp 166–167 °C; ^1^H NMR (400 MHz, DMSO-*d*_6_) δ 12.59 (s, 1H), 9.06 (s, 1H), 8.24 (d, *J* = 7.8 Hz, 1H), 7.96 (d, *J* = 8.0 Hz, 1H), 7.84 (dt, *J* = 26.3, 7.4 Hz, 2H), 7.45–7.37 (m, 1H), 7.35–7.29 (m, 1H), 7.21 (t, *J* = 9.1 Hz, 1H), 7.15–7.06 (m, 1H), 6.49 (dd, *J* = 11.4, 3.0 Hz, 1H), 6.31 (td, *J* = 8.8, 2.9 Hz, 1H), 5.11 (s, 2H), 4.31 (s, 2H), 3.67 (s, 2H), 3.21 (s, 2H), 3.13 (s, 2H), 2.57 (t, *J* = 5.0 Hz, 2H), 2.43 (s, 2H). ^19^F NMR (376 MHz, DMSO-*d*_6_) δ -117.01, -119.85. ^13^C NMR (100 MHz, DMSO-*d*_6_) δ 168.81, 164.30, 162.33, 159.96, 159.88, 158.05, 155.62, 145.39, 144.97, 144.85, 135.34, 135.30, 133.98, 132.05, 131.99, 129.58, 129.27, 129.23, 128.41, 127.75, 127.65, 126.58, 125.95, 124.44, 124.26, 119.85, 116.48, 116.27, 102.83, 102.60, 102.31, 102.05, 61.38, 53.28, 52.84, 47.02, 41.82, 36.92. HRMS (ESI) m/z calculated for [M+H]**^+^** 533.21118, found 533.21072.

N-(2-aminophenyl)-3-(4-(2-fluoro-5-((4-oxo-3,4-dihydrophthalazin-1-yl)methyl)benzoyl)piperazin-1-yl)propanamide (**B501)**; yellow solid; yield 74%; mp 131–132 °C; ^1^H NMR (400 MHz, DMSO-*d*_6_) δ 12.59 (s, 1H), 9.23 (s, 1H), 8.24 (d, *J* = 7.8 Hz, 1H), 7.97–7.77 (m, 3H), 7.40 (s, 1H), 7.32–7.02 (m, 4H), 6.88 (t, *J* = 8.0 Hz, 1H), 6.67 (d, *J* = 8.1 Hz, 1H), 6.49 (d, *J* = 8.1 Hz, 1H), 4.91 (s, 2H), 4.31 (s, 2H), 3.60 (s, 2H), 3.14 (s, 2H), 3.03 (s, 2H), 2.63 (s, 2H), 2.31 (s, 2H), 1.22 (d, *J* = 8.1 Hz, 2H). ^13^C NMR (100 MHz, DMSO-*d*_6_) δ 170.50, 164.22, 159.88, 145.42, 143.17, 135.34, 134.02, 133.99, 132.17, 132.13, 132.09, 132.04, 129.55, 129.26, 129.21, 128.38, 126.72, 126.66, 126.57, 126.38, 125.97, 123.70, 116.56, 116.52, 116.48, 116.30, 115.94, 54.45, 53.14, 46.98, 41.74, 36.89, 36.87, 33.89. HRMS (ESI) m/z calculated for [M+H]**^+^** 529.23579, found 529.23621.

N-(2-amino-4-fluorophenyl)-3-(4-(2-fluoro-5-((4-oxo-3,4-dihydrophthalazin-1-yl)methyl)benzoyl)piperazin-1-yl)propanamide (**B502**); yellow solid; yield 64%，mp 119–120 °C; ^1^H NMR (400 MHz, DMSO-*d*_6_) δ 12.61 (s, 1H), 9.17 (s, 1H), 8.35–8.18 (m, 1H), 8.03–7.77 (m, 3H), 7.47–7.15 (m, 3H), 7.11–6.89 (m, 1H), 6.44 (d, *J* = 11.3 Hz, 1H), 6.34–6.19 (m, 1H), 5.28 (s, 2H), 4.31 (s, 2H), 3.60 (s, 2H), 3.14 (s, 2H), 2.62 (s, 4H), 2.30 (s, 4H). ^13^C NMR (100 MHz, DMSO-*d*_6_) δ 170.77, 164.25, 159.87, 145.38, 133.99, 132.06, 129.57, 128.41, 126.57, 125.96, 52.50, 46.93, 36.91, 33.89. HRMS (ESI) m/z calculated for [M+H]**^+^** 547.22637, found 547.22656.

N-(2-aminophenyl)-4-(4-(2-fluoro-5-((4-oxo-3,4-dihydrophthalazin-1-yl)methyl)benzoyl)piperazin-1-yl)butanamide (**B601**); yellow oil; yield 82%; ^1^H NMR (400 MHz, DMSO-*d*_6_) δ 12.59 (s, 1H), 9.08 (s, 1H), 8.24 (d, *J* = 7.9 Hz, 1H), 7.98–7.76 (m, 3H), 7.40 (s, 1H), 7.31–7.08 (m, 3H), 6.86 (t, *J* = 7.6 Hz, 1H), 6.68 (d, *J* = 8.1 Hz, 1H), 6.51 (t, *J* = 7.8 Hz, 1H), 4.82 (s, 2H), 4.31 (s, 2H), 3.59 (s, 2H), 3.13 (s, 2H), 2.87 (s, 1H), 2.71 (s, 1H), 2.36–2.18 (m, 6H), 1.80–1.64 (m, 2H). ^13^C NMR (100 MHz, DMSO-*d*_6_) δ 171.55, 164.25, 159.92, 158.05, 155.62, 145.43, 142.36, 135.30, 135.27, 134.01, 132.07, 129.56, 129.20, 128.37, 126.57, 126.24, 125.95, 125.79, 124.39, 124.20, 124.05, 121.69, 116.73, 116.50, 116.40, 116.29, 57.48, 53.17, 52.72, 46.92, 41.73, 36.92, 34.10, 22.67.HRMS (ESI) m/z calculated for [M+H]**^+^** 543.25146, found 543.25144.

N-(2-amino-4-fluorophenyl)-4-(4-(2-fluoro-5-((4-oxo-3,4-dihydrophthalazin-1-yl)methyl)benzoyl)piperazin-1-yl)butanamide (**B602**); brown oil; yield 89%; ^1^H NMR (400 MHz, DMSO-*d*_6_) δ 12.59 (s, 1H), 9.01 (s, 1H), 8.24 (d, *J* = 7.8 Hz, 1H), 8.02–7.77 (m, 3H), 7.43–7.00 (m, 4H), 6.56–6.40 (m, 1H), 6.29 (d, *J* = 9.2 Hz, 1H), 5.13 (s, 2H), 4.31 (s, 2H), 3.58 (s, 2H), 3.12 (s, 2H), 2.87 (s, 1H), 2.71 (s, 1H), 2.33–2.13 (m, 6H), 1.77–1.63 (m, 2H). ^13^C NMR (100 MHz, DMSO-*d*_6_) δ 171.79, 164.22, 162.17, 159.90, 159.80, 156.82 (d, *J* = 244.9 Hz), 145.42, 144.78, 144.66, 135.29 (d, *J* = 3.5 Hz), 134.00, 132.07, 129.56, 129.16, 128.38, 127.62, 127.52, 126.57, 125.96, 124.44, 124.25, 120.06, 116.50, 116.28, 102.53, 102.31, 101.97, 101.72, 57.56, 53.27, 52.80, 47.05, 41.85, 36.91, 34.04, 22.71. HRMS (ESI) m/z calculated for [M+H]**^+^** 561.24202, found 561.24207.

[1] Z. Yuan, S. Chen, Q. Sun, N. Wang, D. Li, S. Miao, C. Gao, Y. Chen, C. Tan, Y. Jiang, Olaparib hydroxamic acid derivatives as dual PARP and HDAC inhibitors for cancer therapy, Bioorg. Med. Chem., 25 (2017) 4100-4109.

# HPLC data of compounds B101–B602

HPLC analysis: Method: Agilent: LC1260II, Column: Shim-pack GIST C18, 5 μm, 4.6 × 250 mm, flow rate of 1 mL/min, HPLC analysis: MeOH-H_2_O (0.01% TFA), gradient elution, 0 min, 90:10, 25 min, 5:95.

**B101**


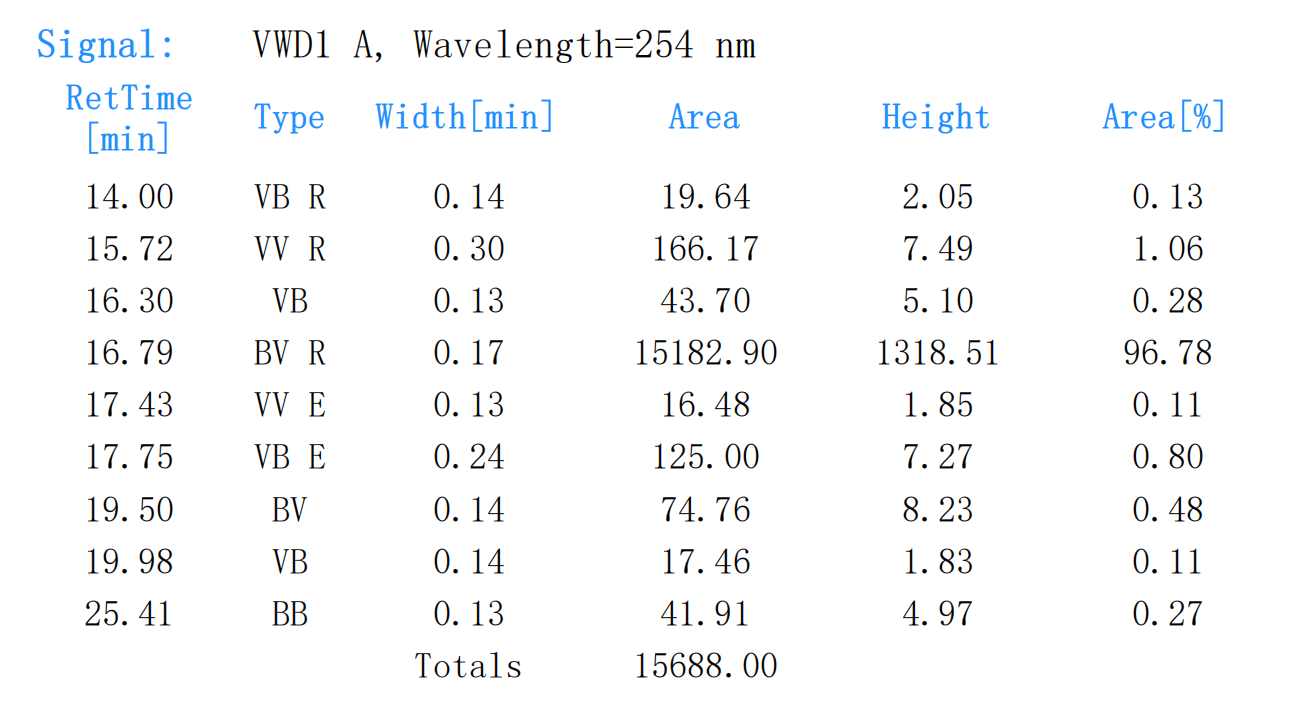


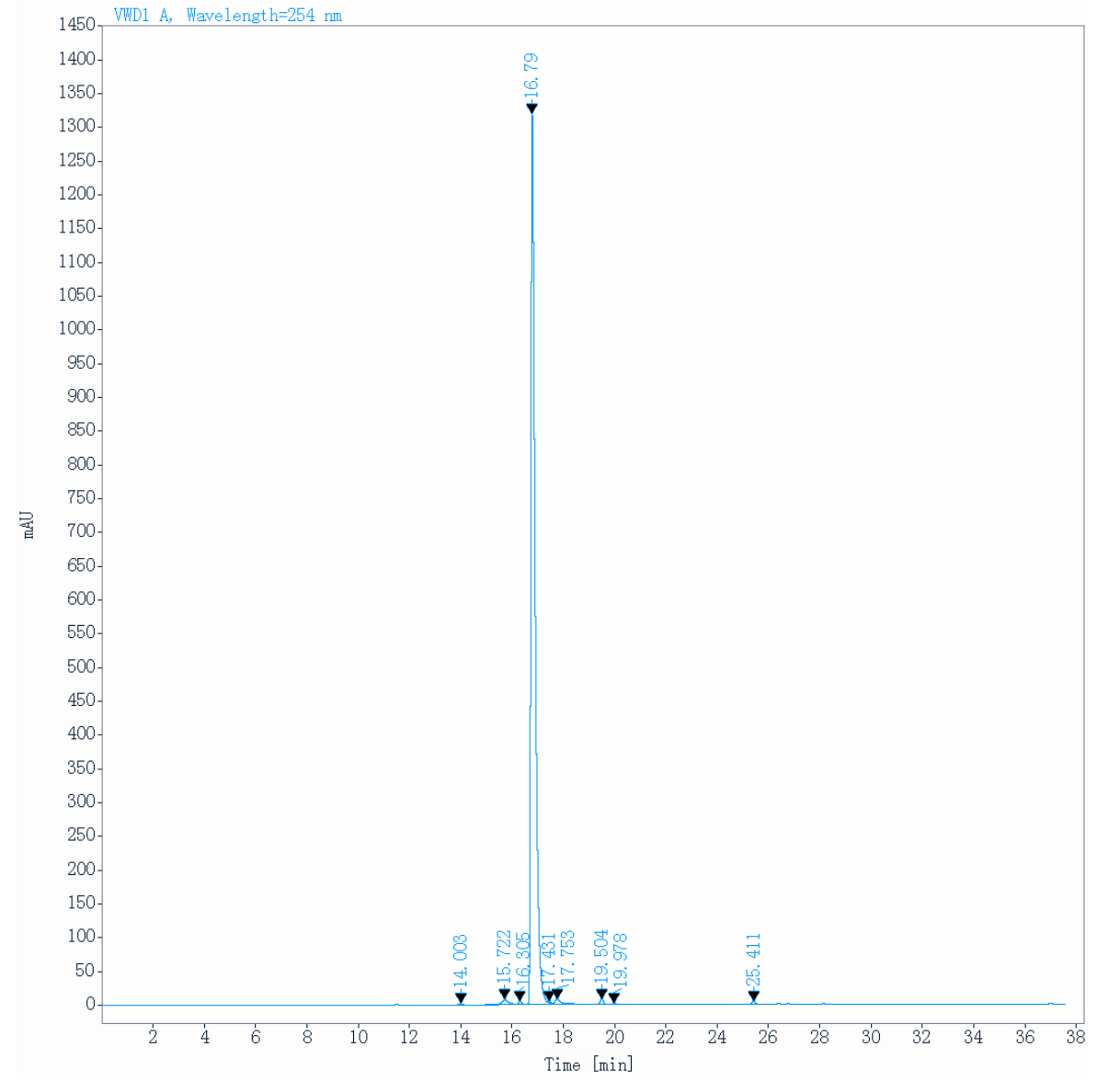


**B102**


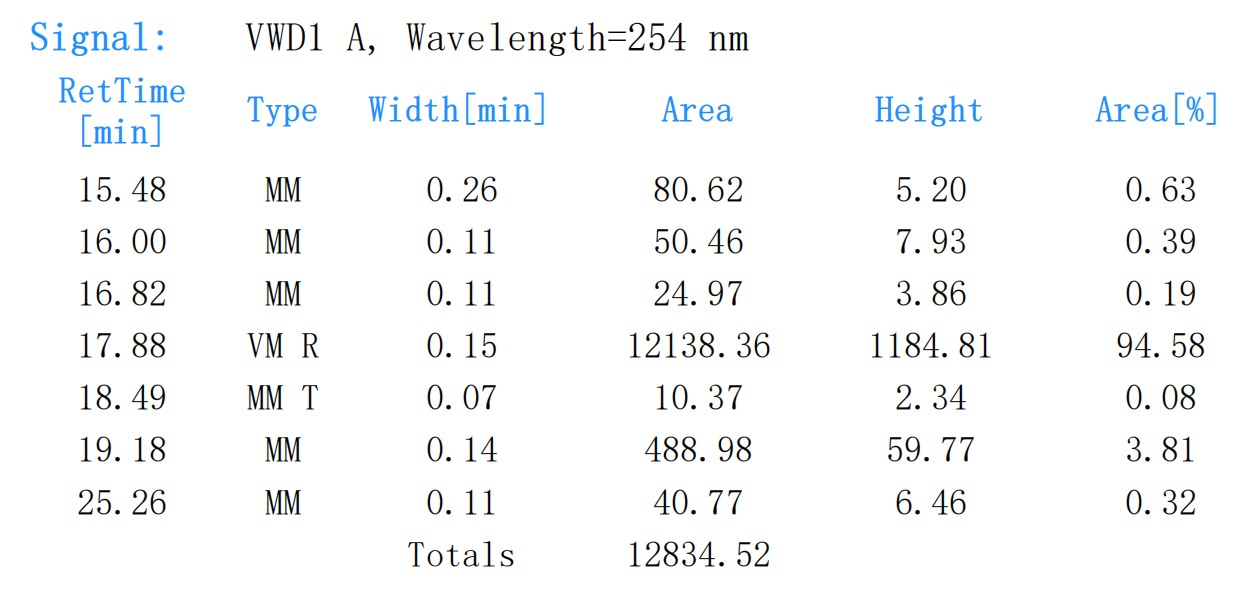


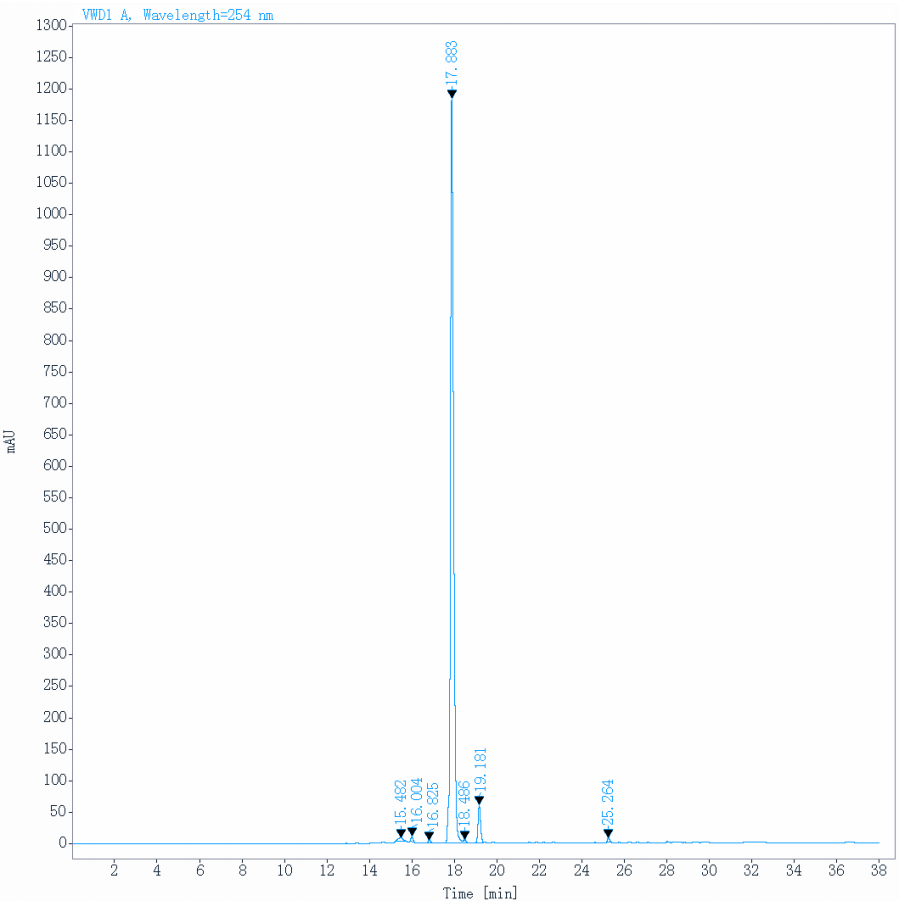


**B201**


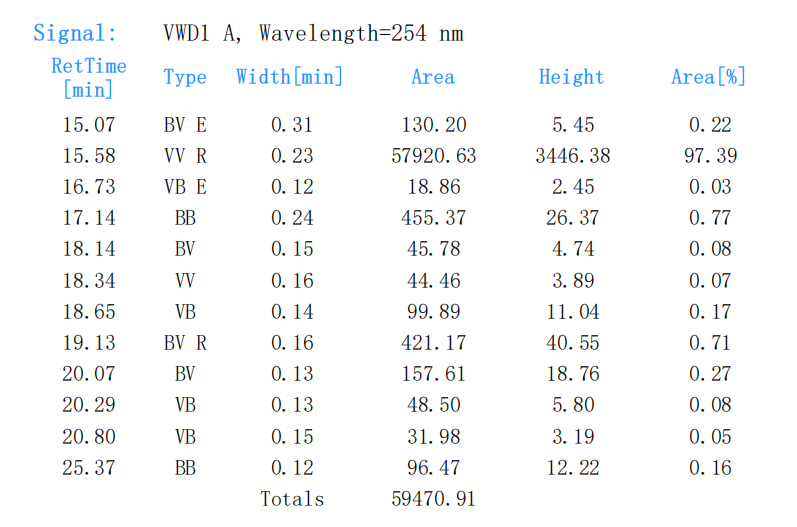


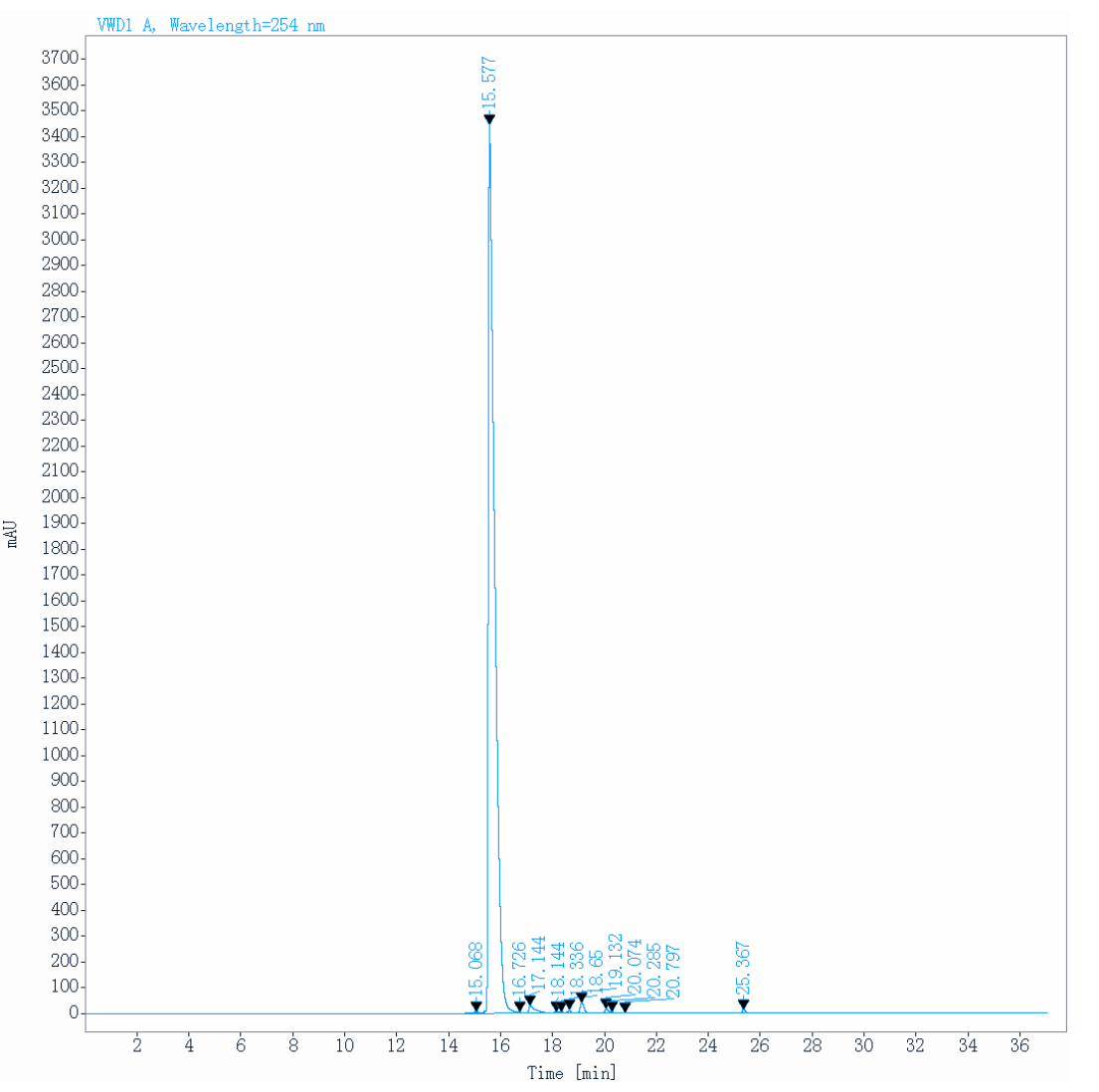


**B202**


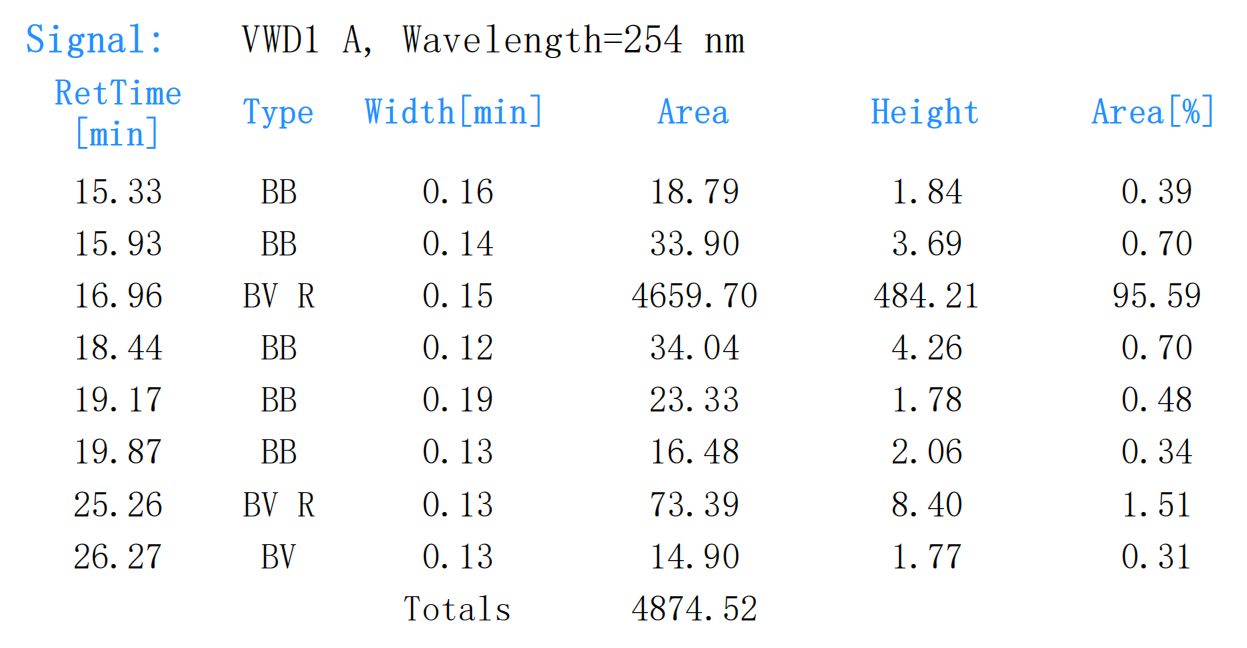


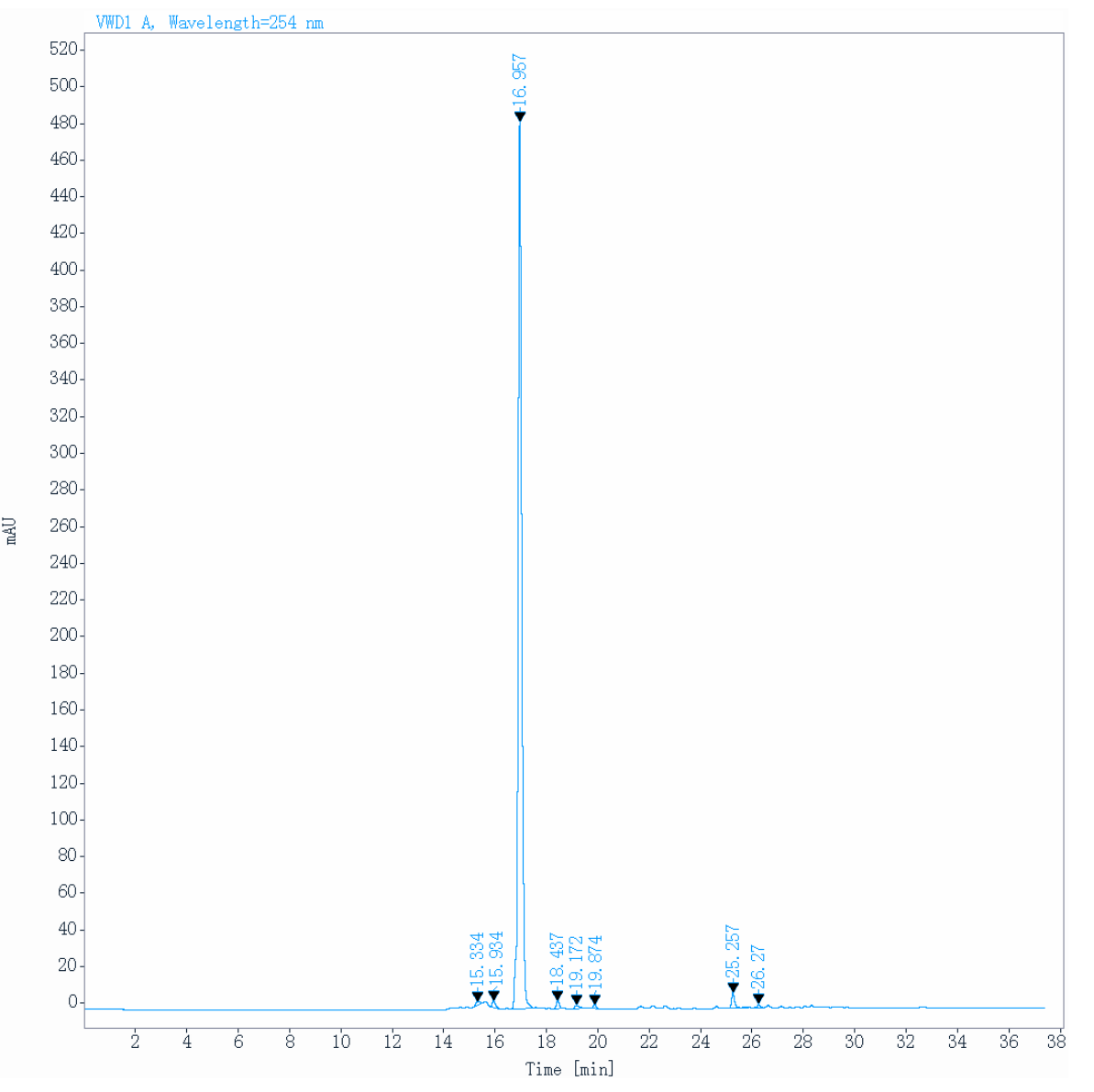


**B301**


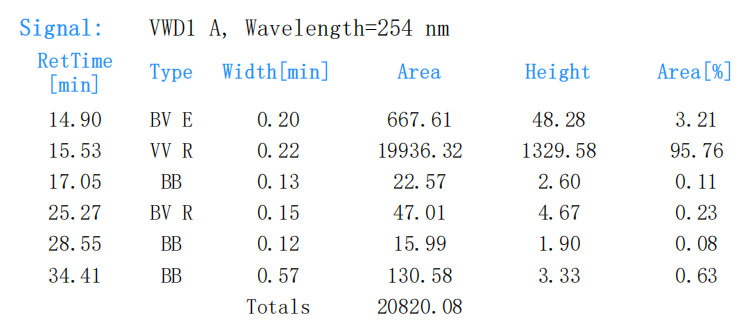


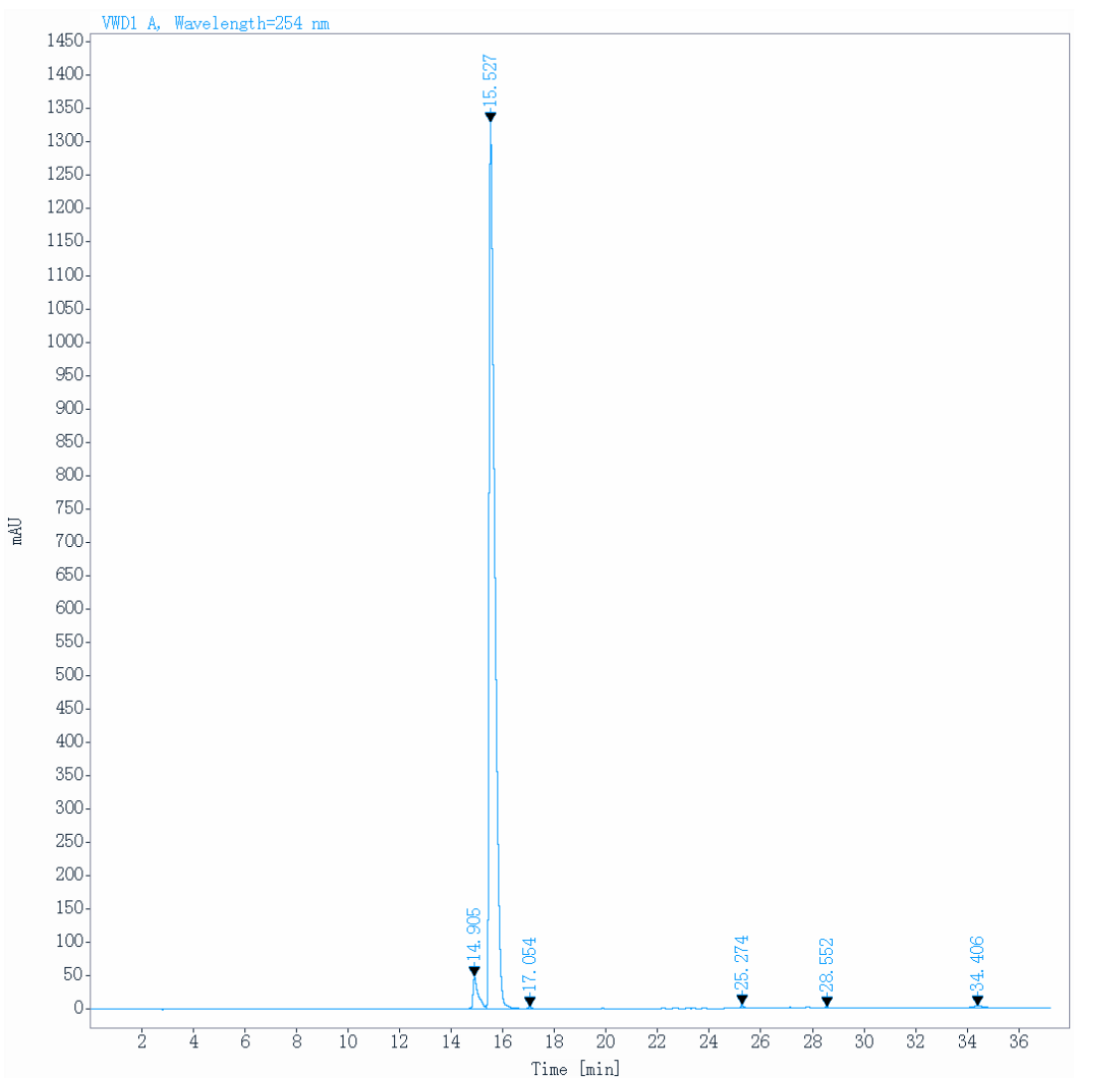


**B302**


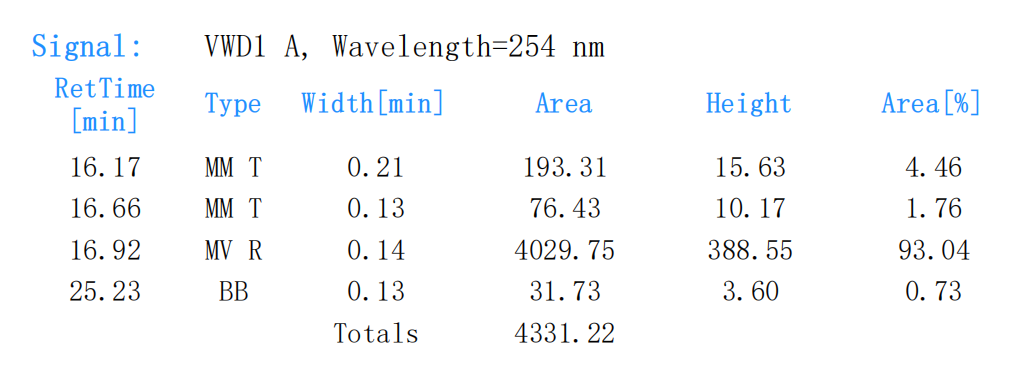


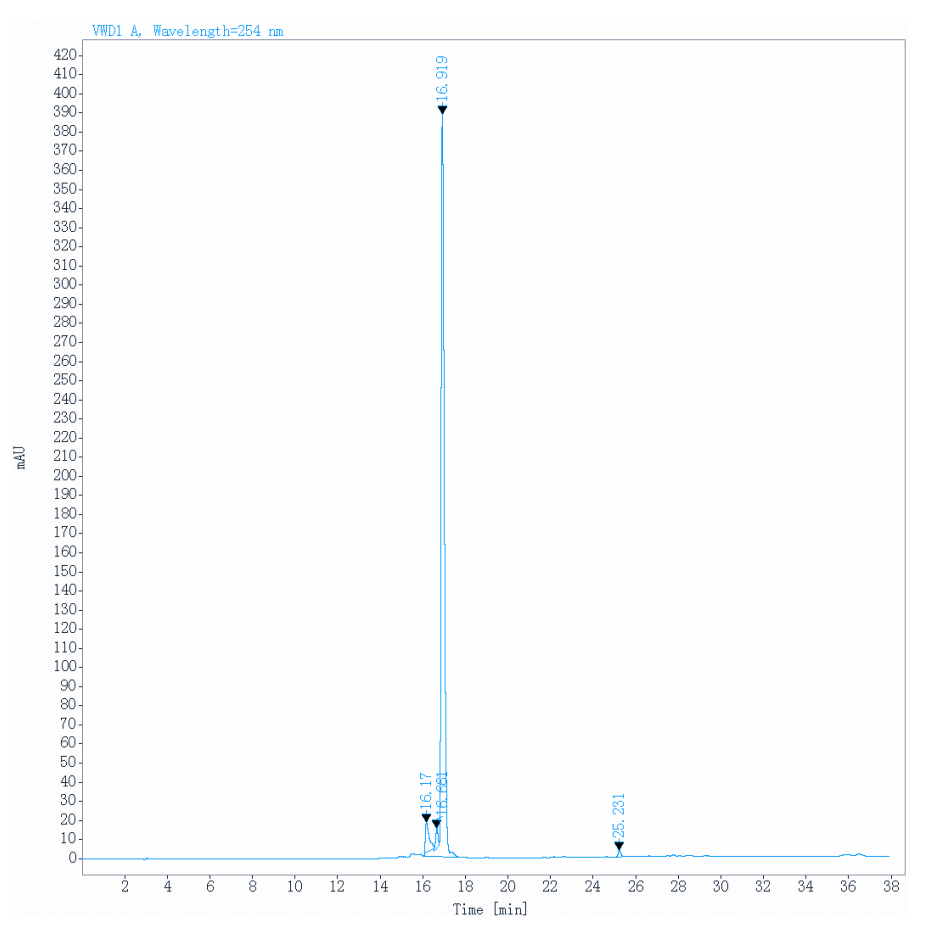


**B401**


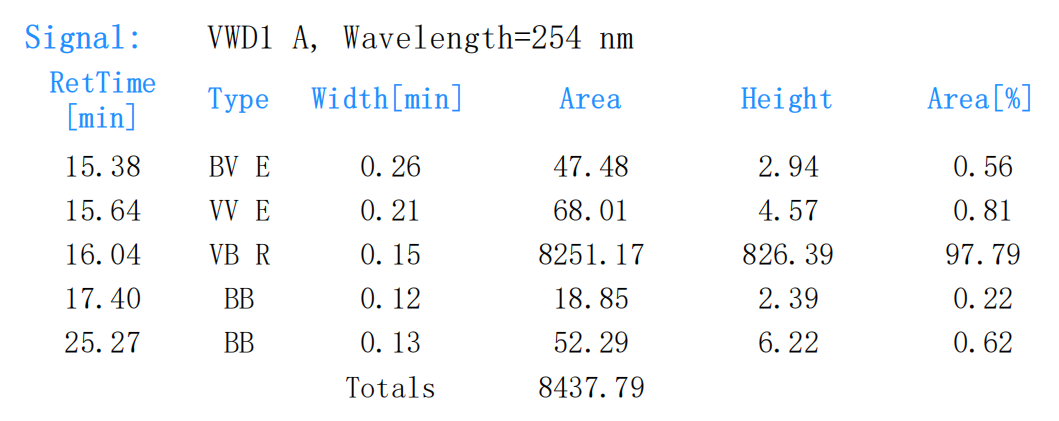


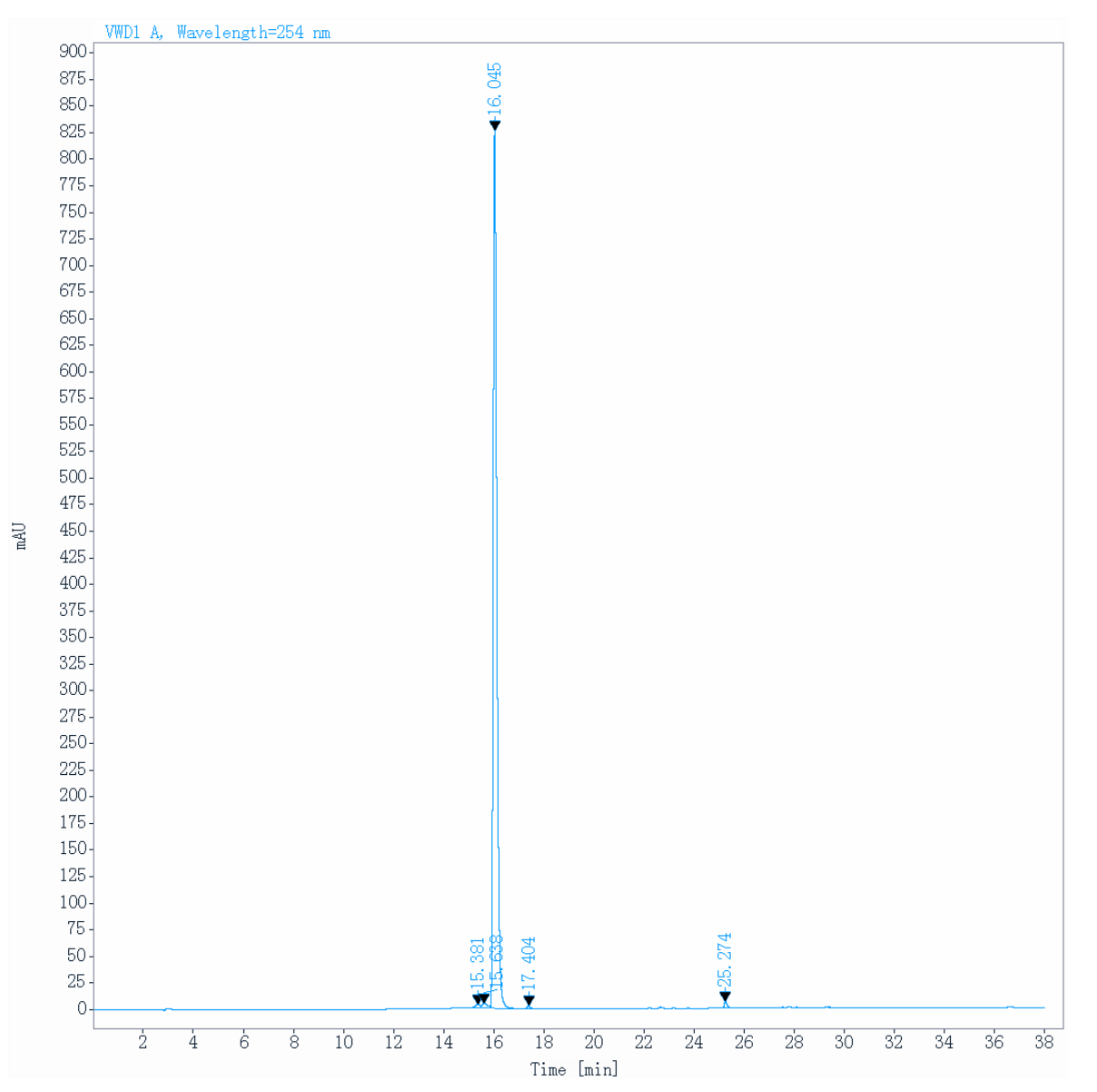


**B402**


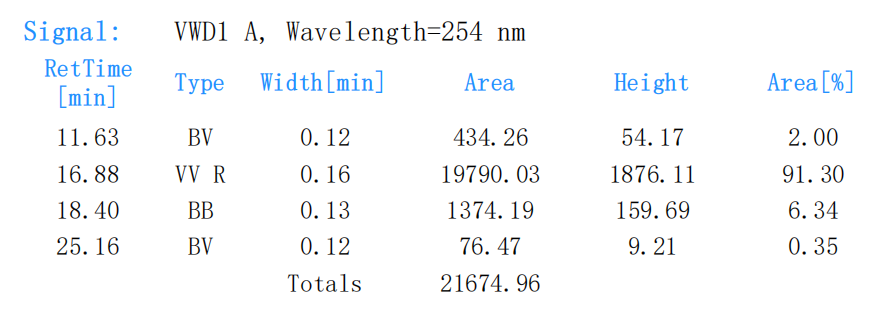


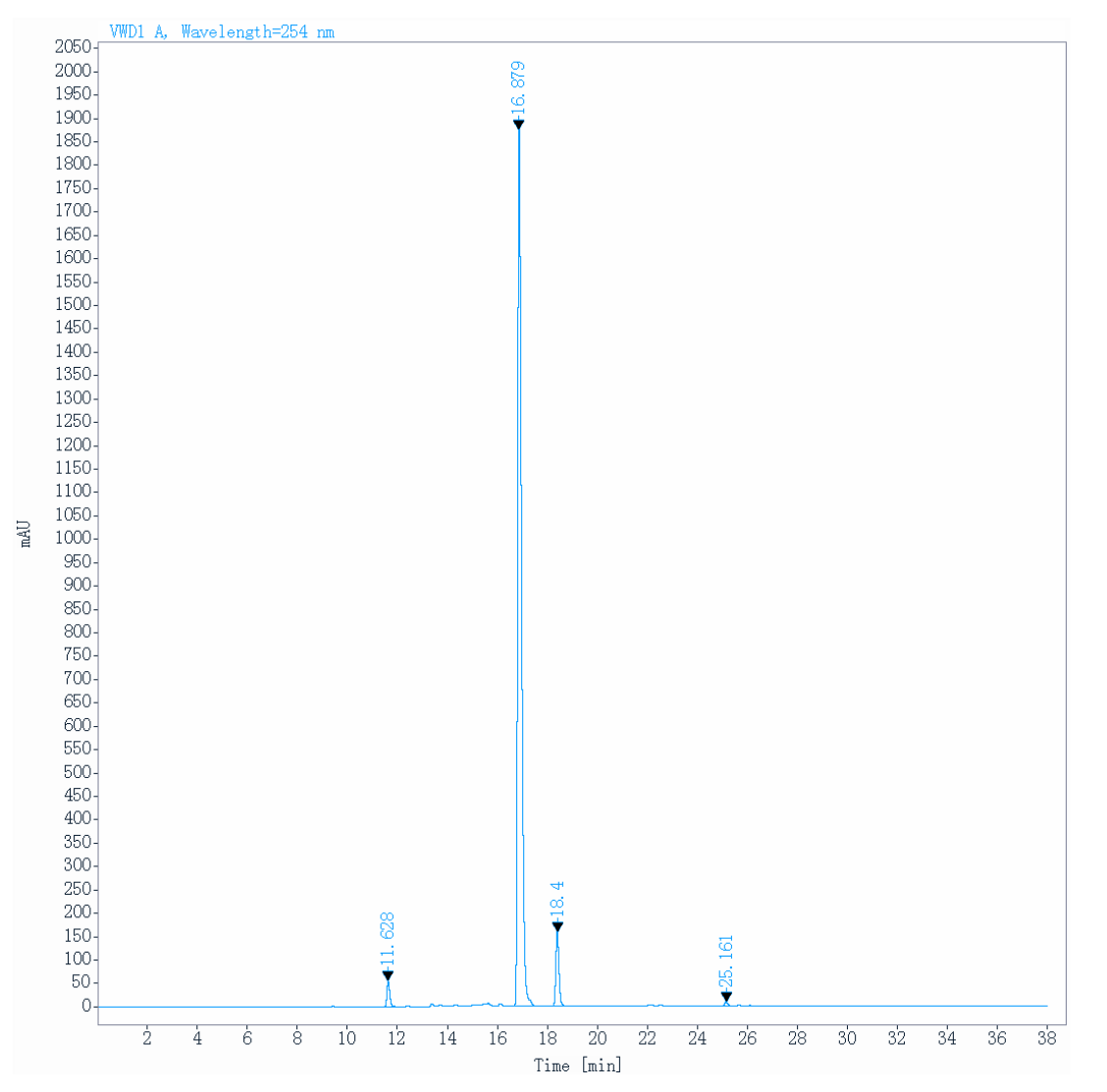


**B501**


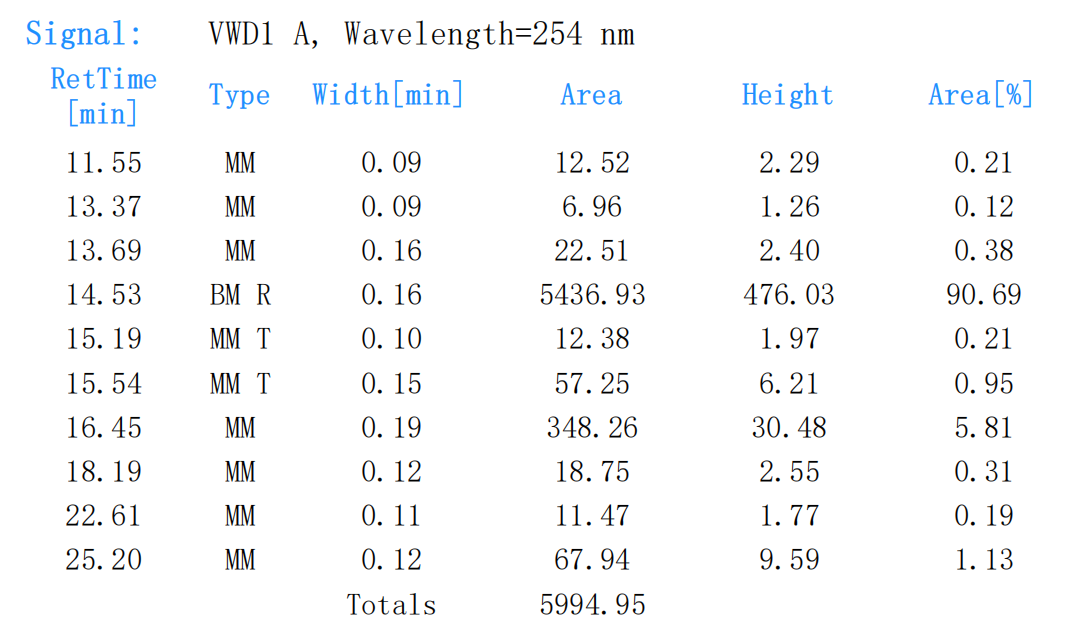


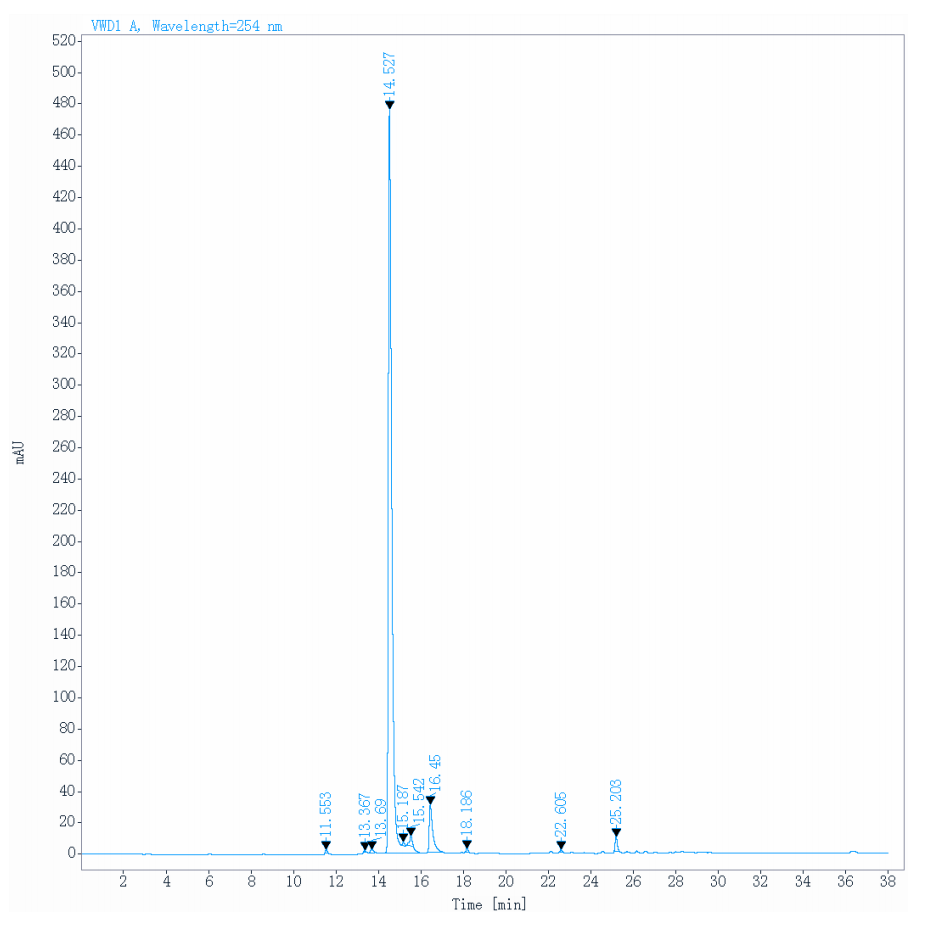


**B502**


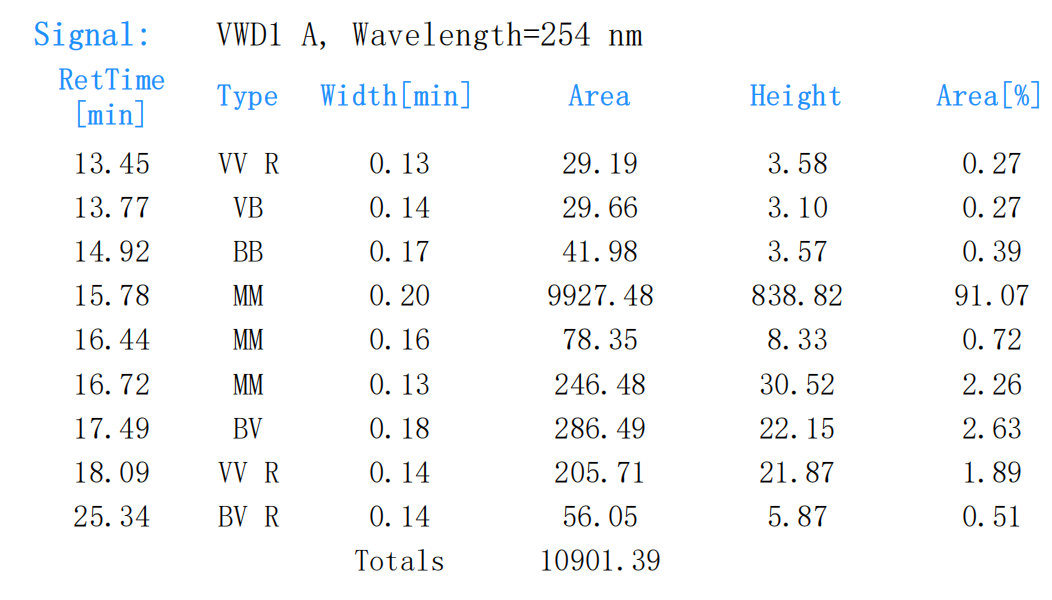


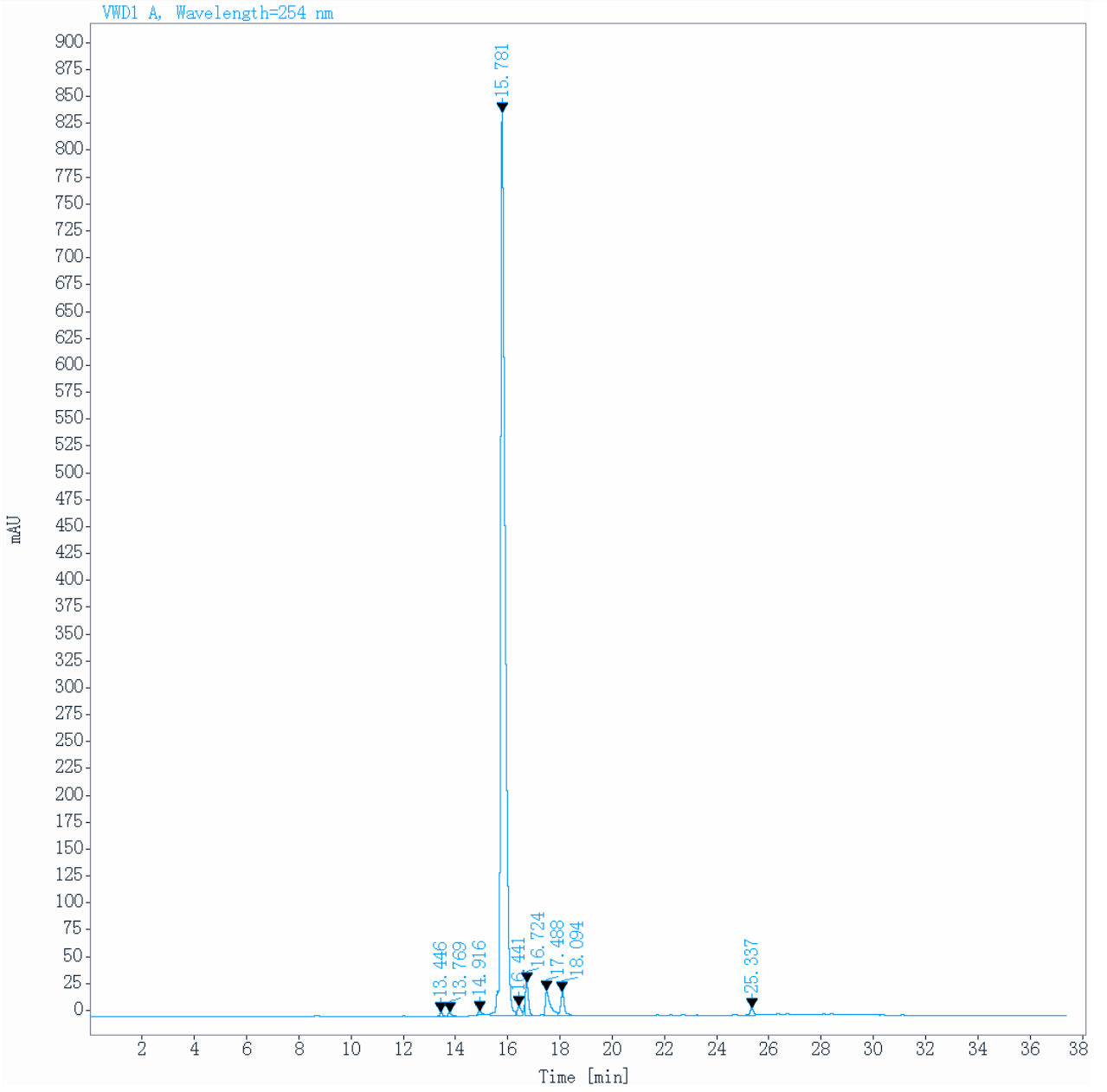


**B601**


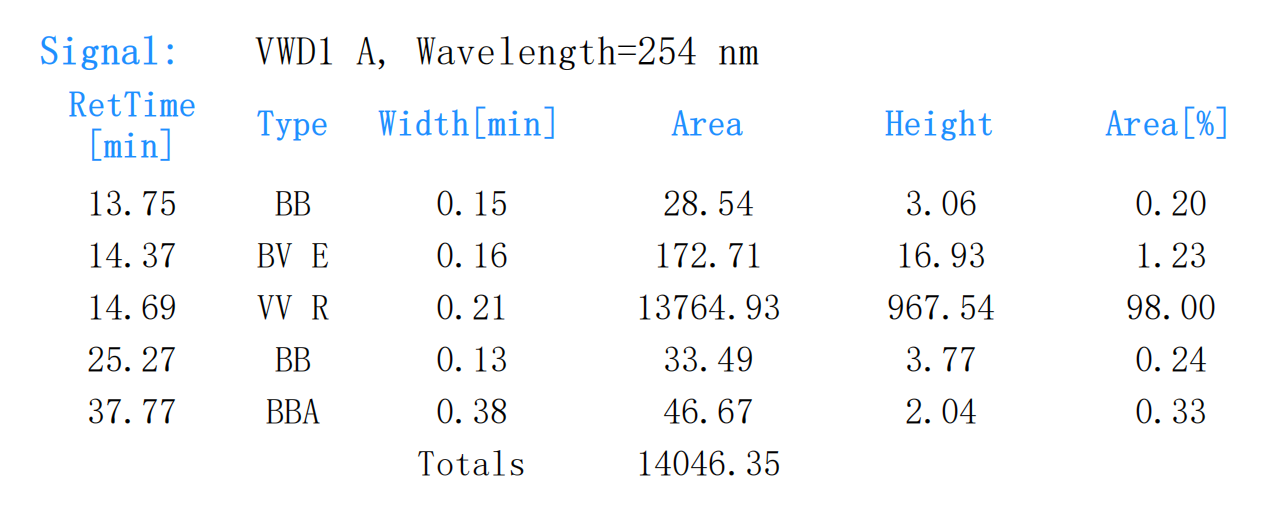


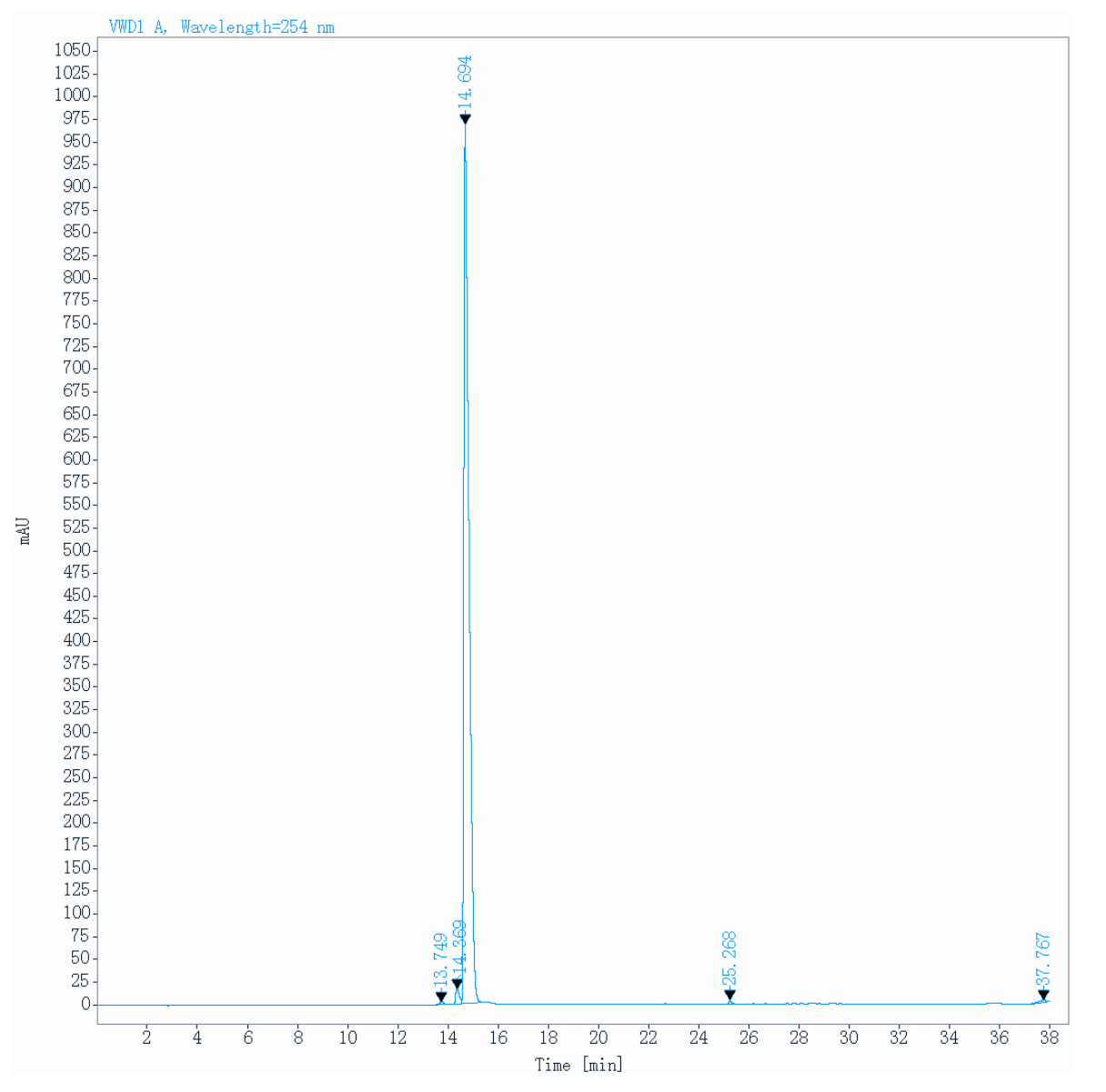


**B602**


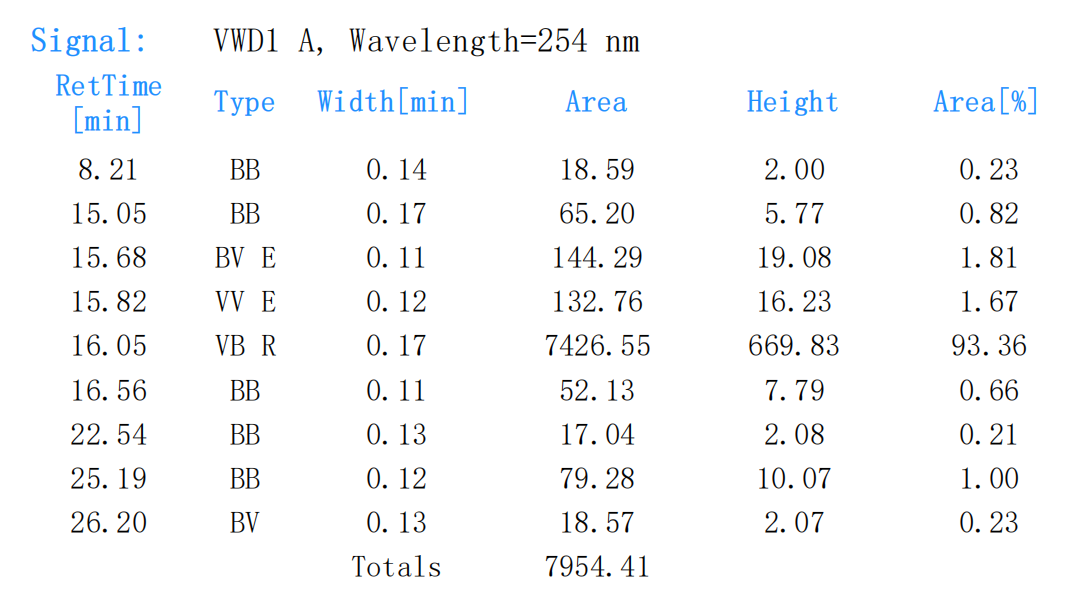


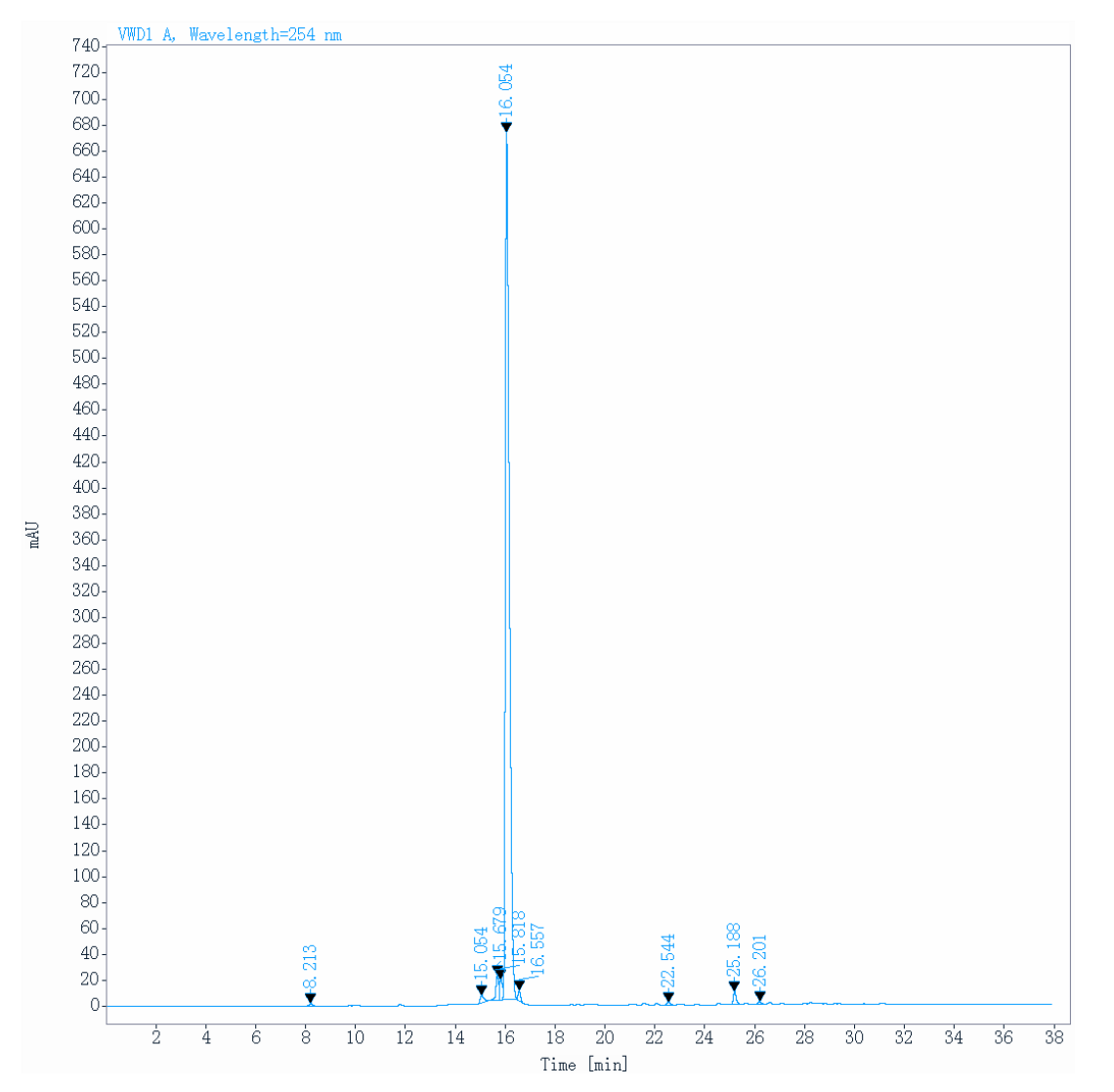


# NMR data of compounds B101–B602

**B101**


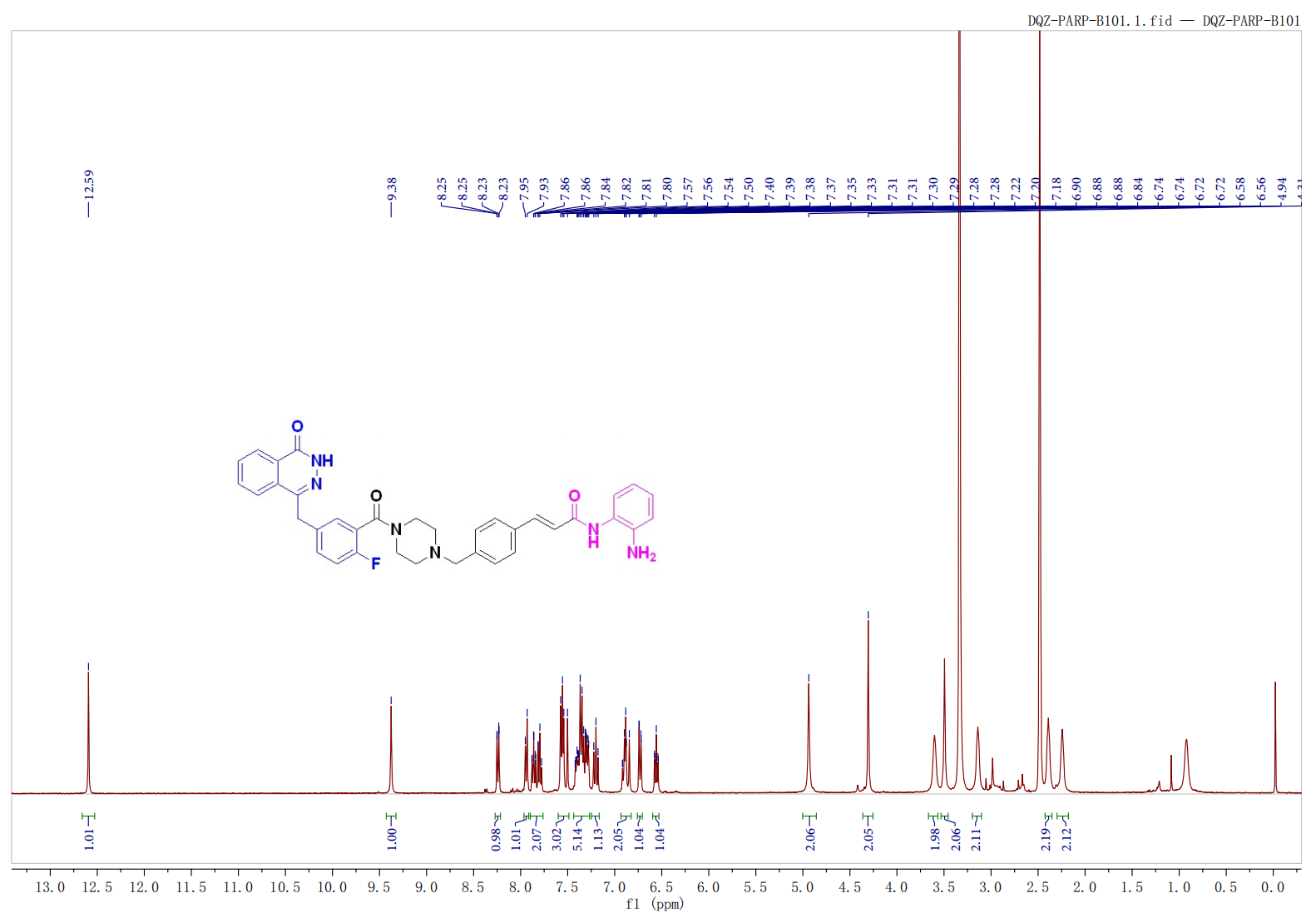


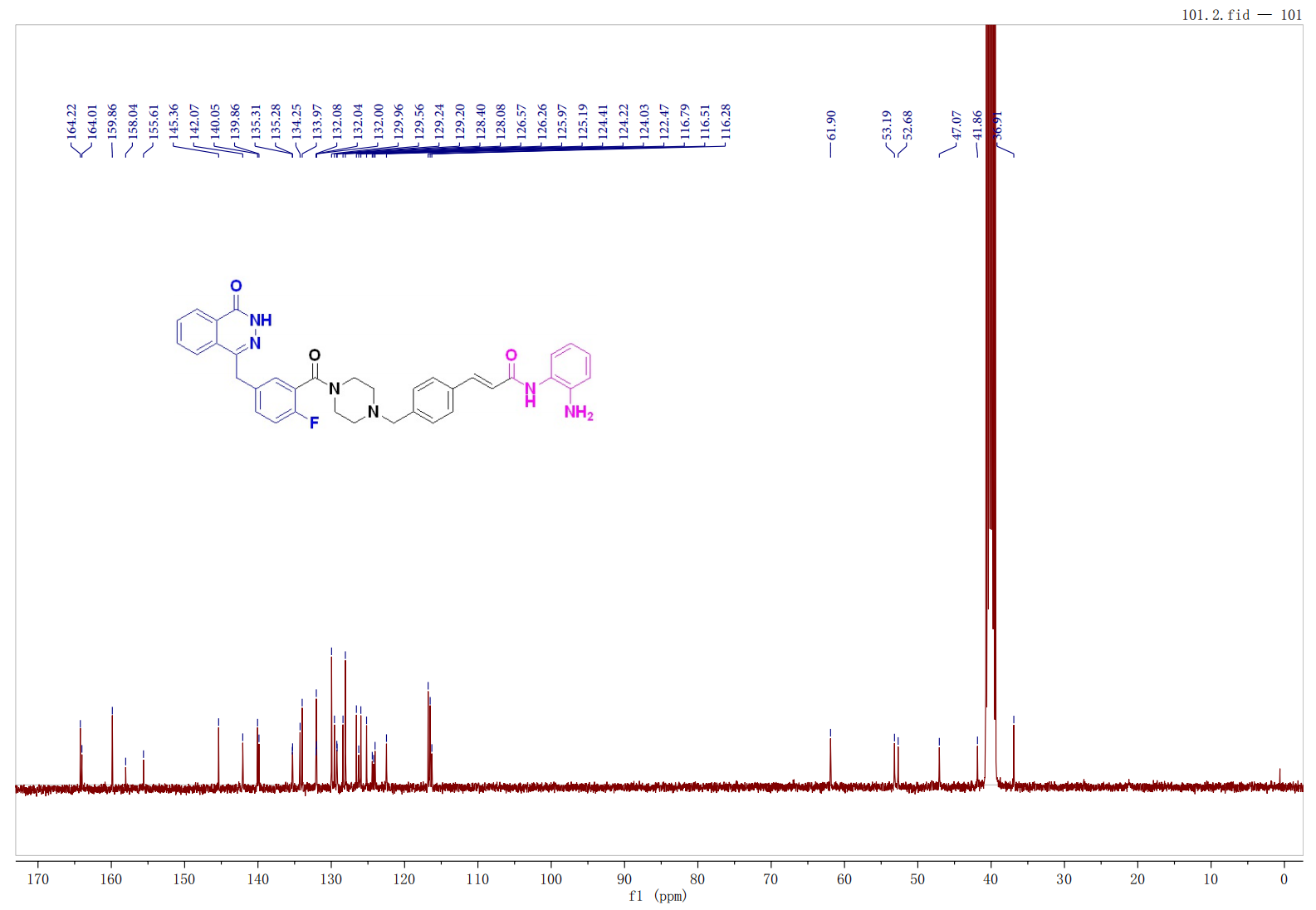


**B102**


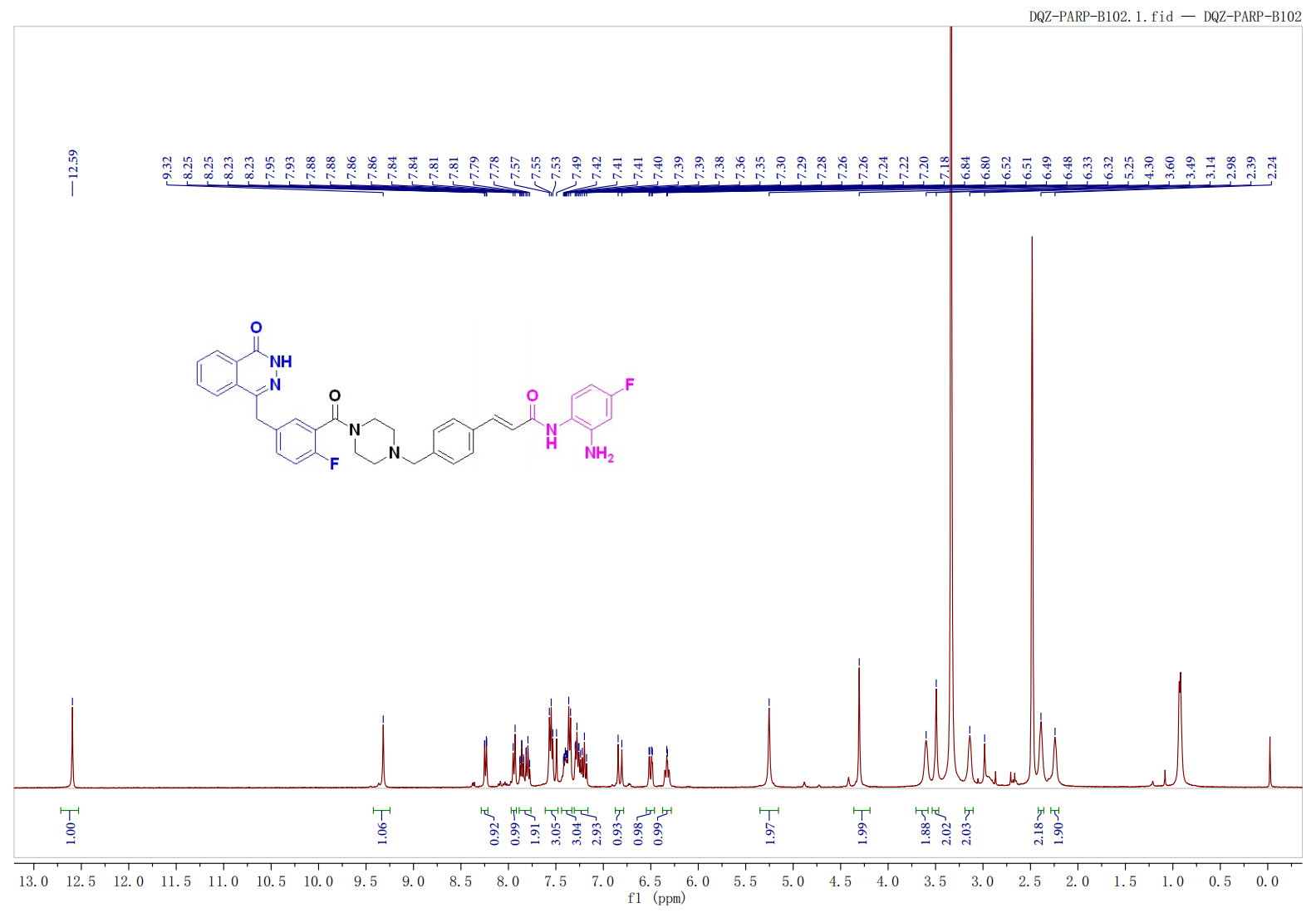


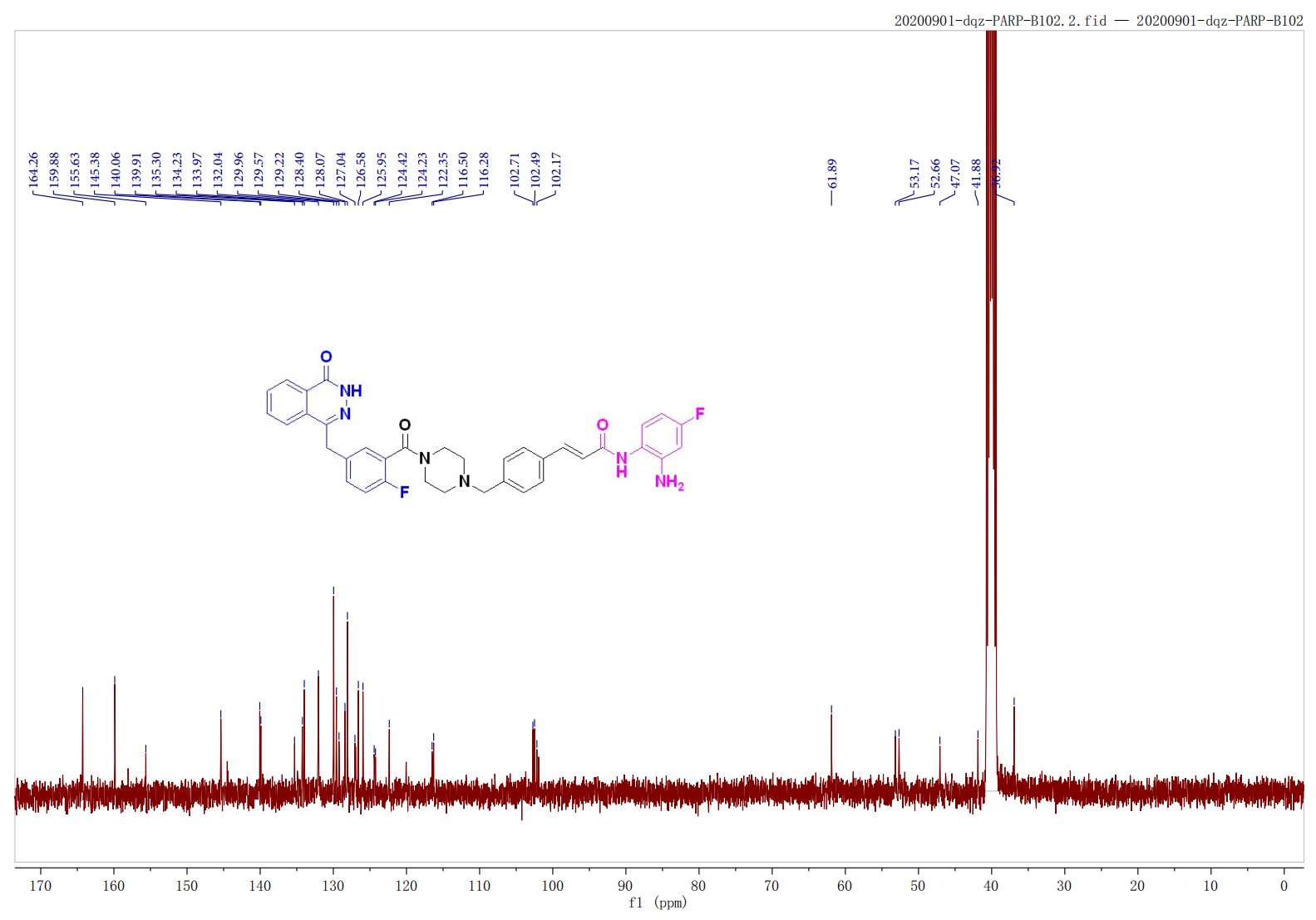


**B201**


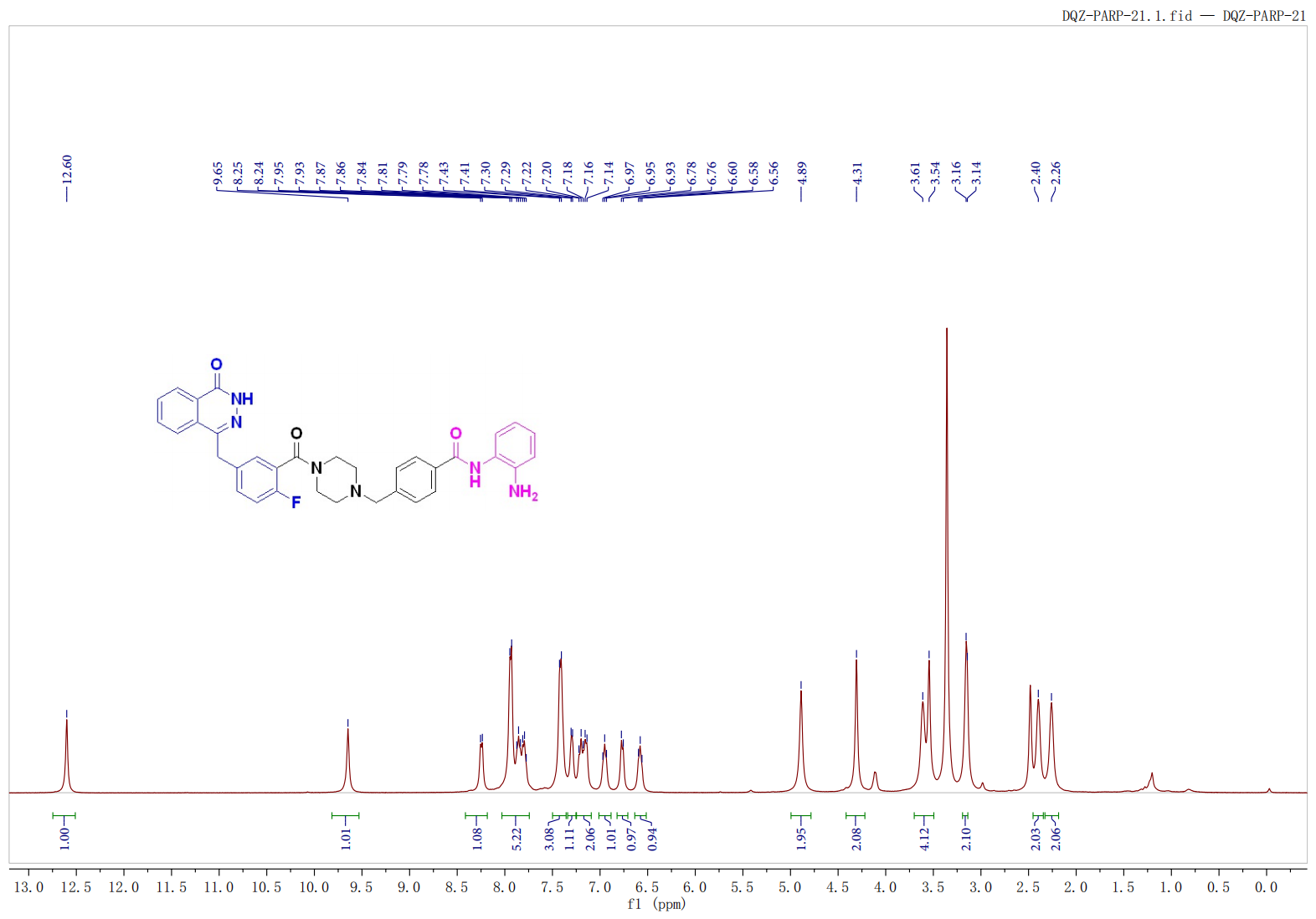


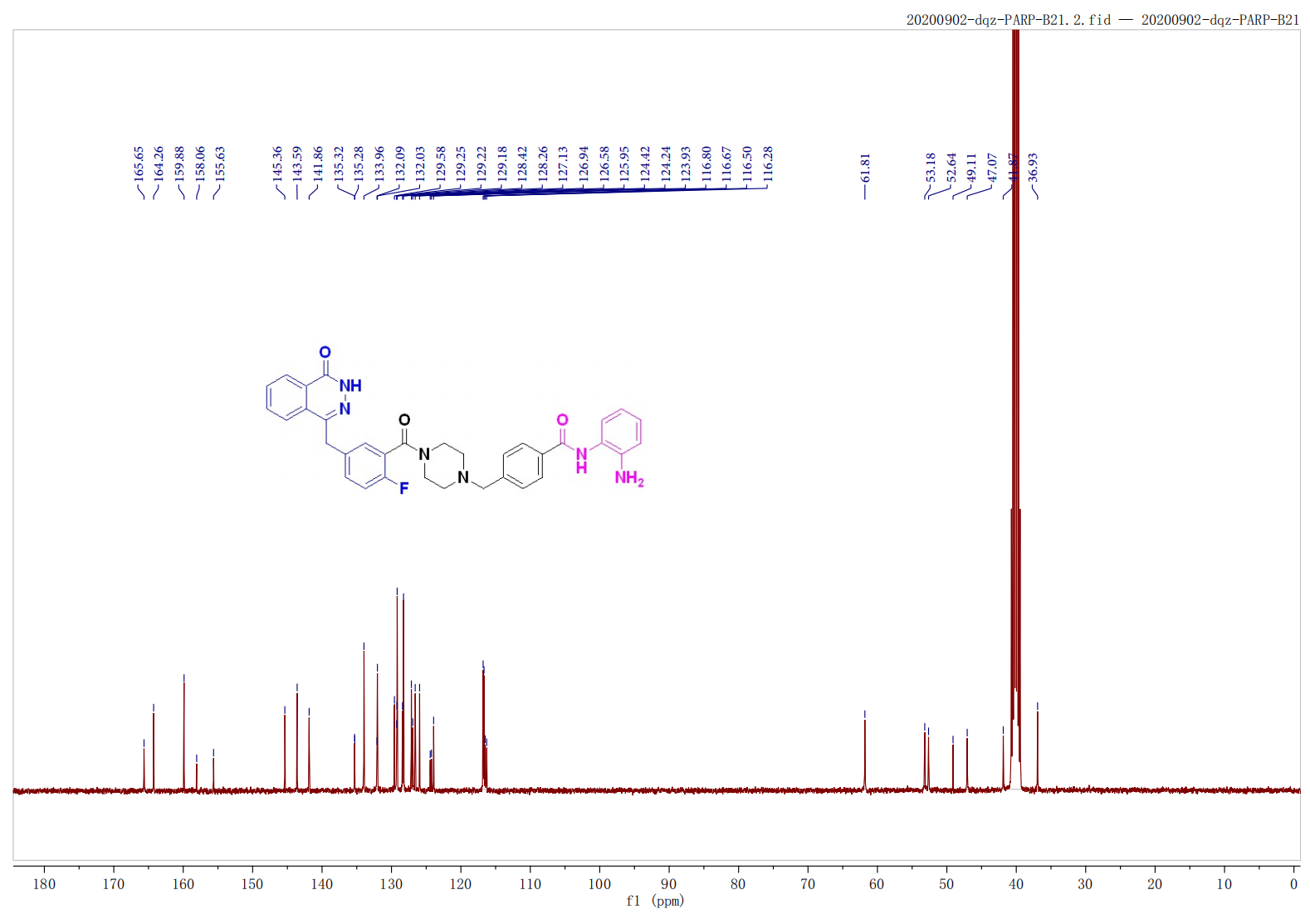


**B202**


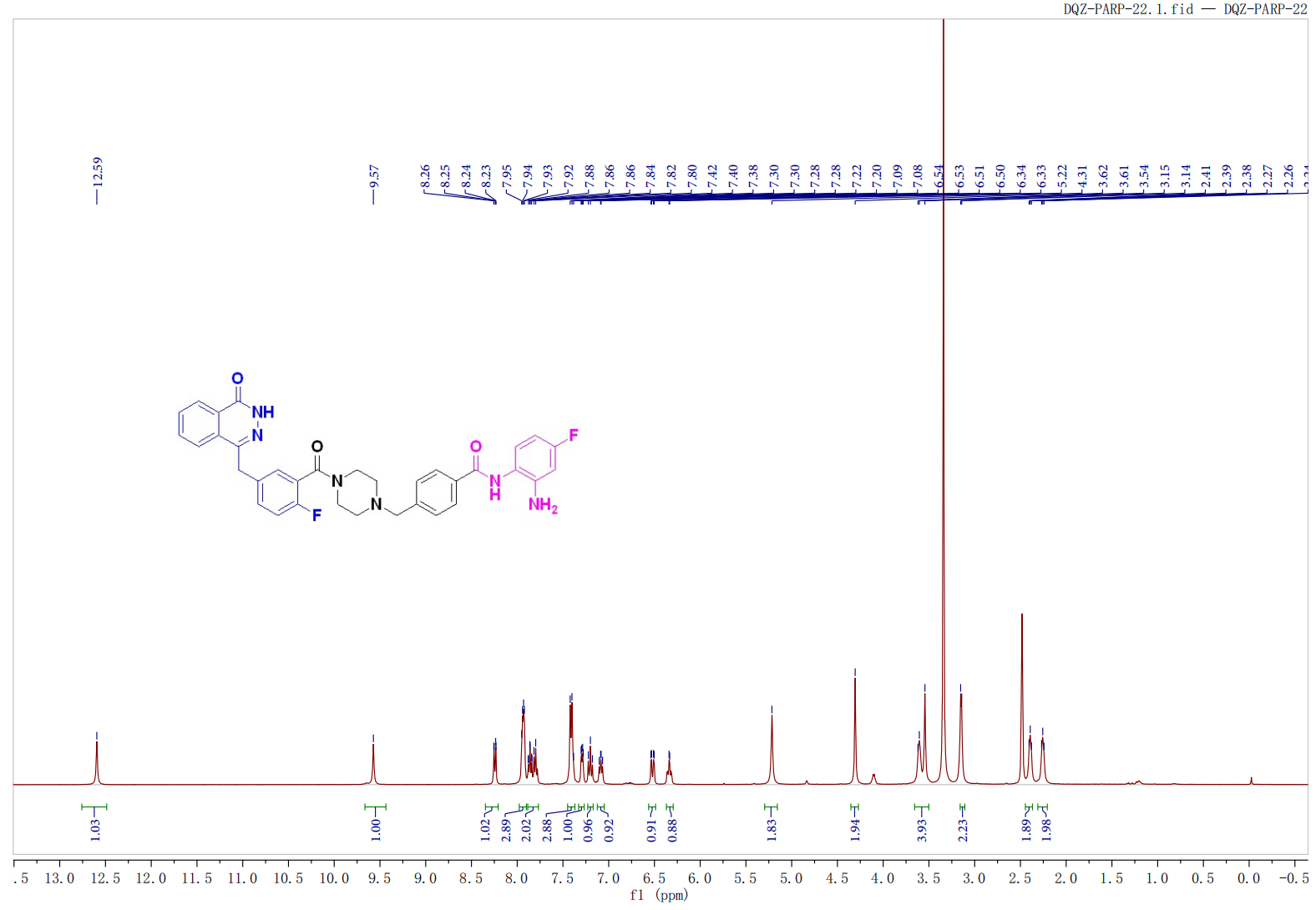


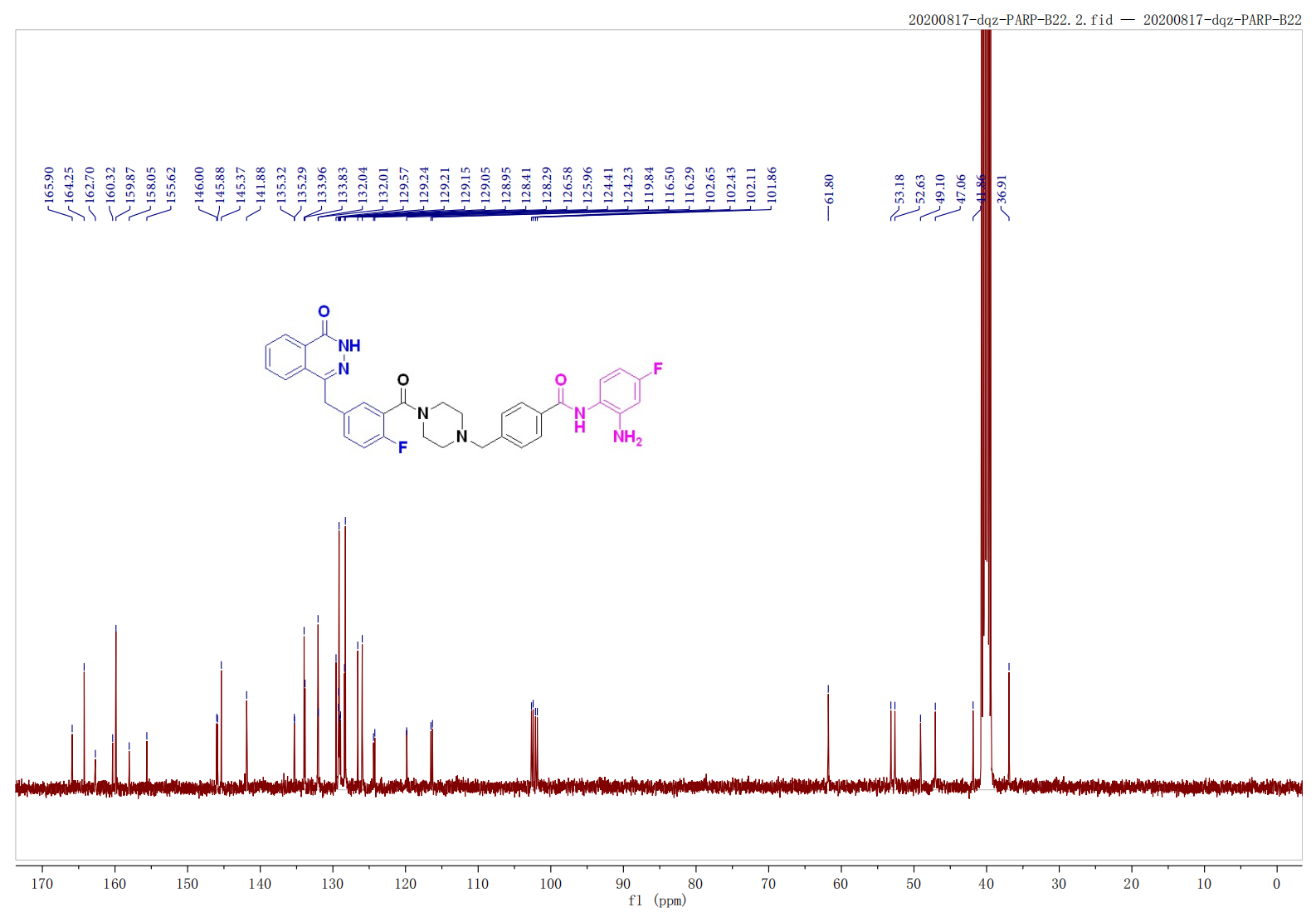


**B301**


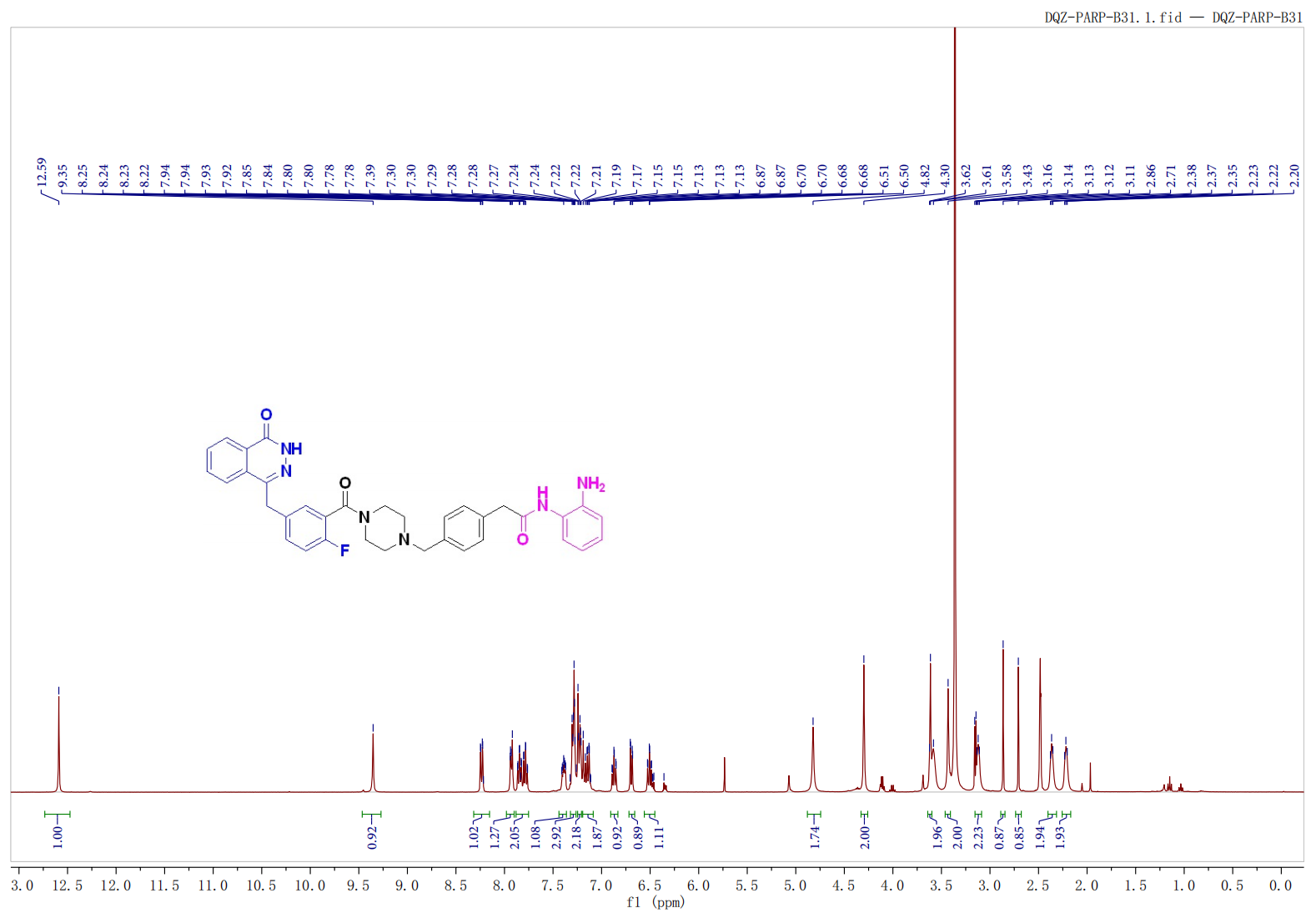


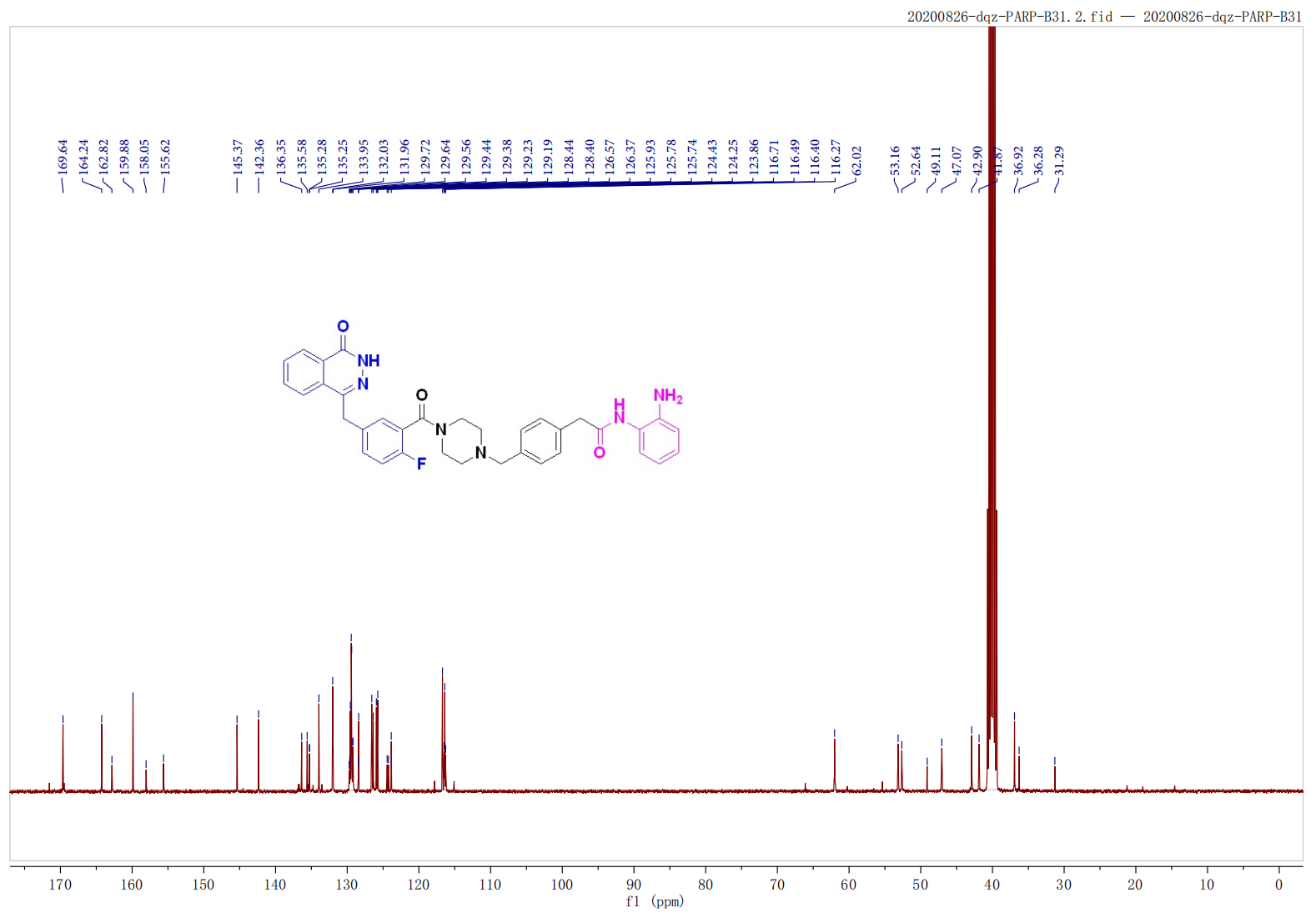


**B302**


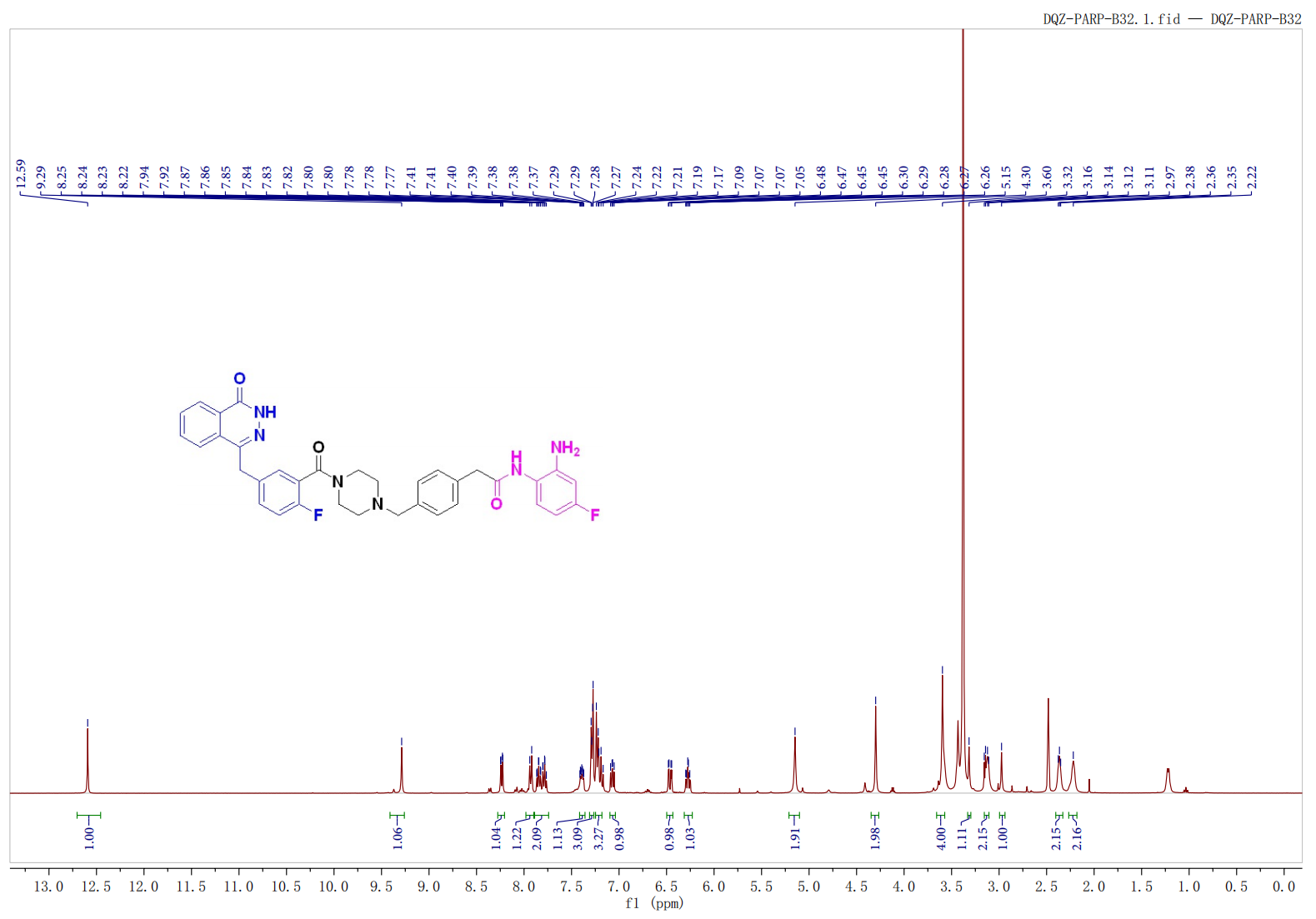


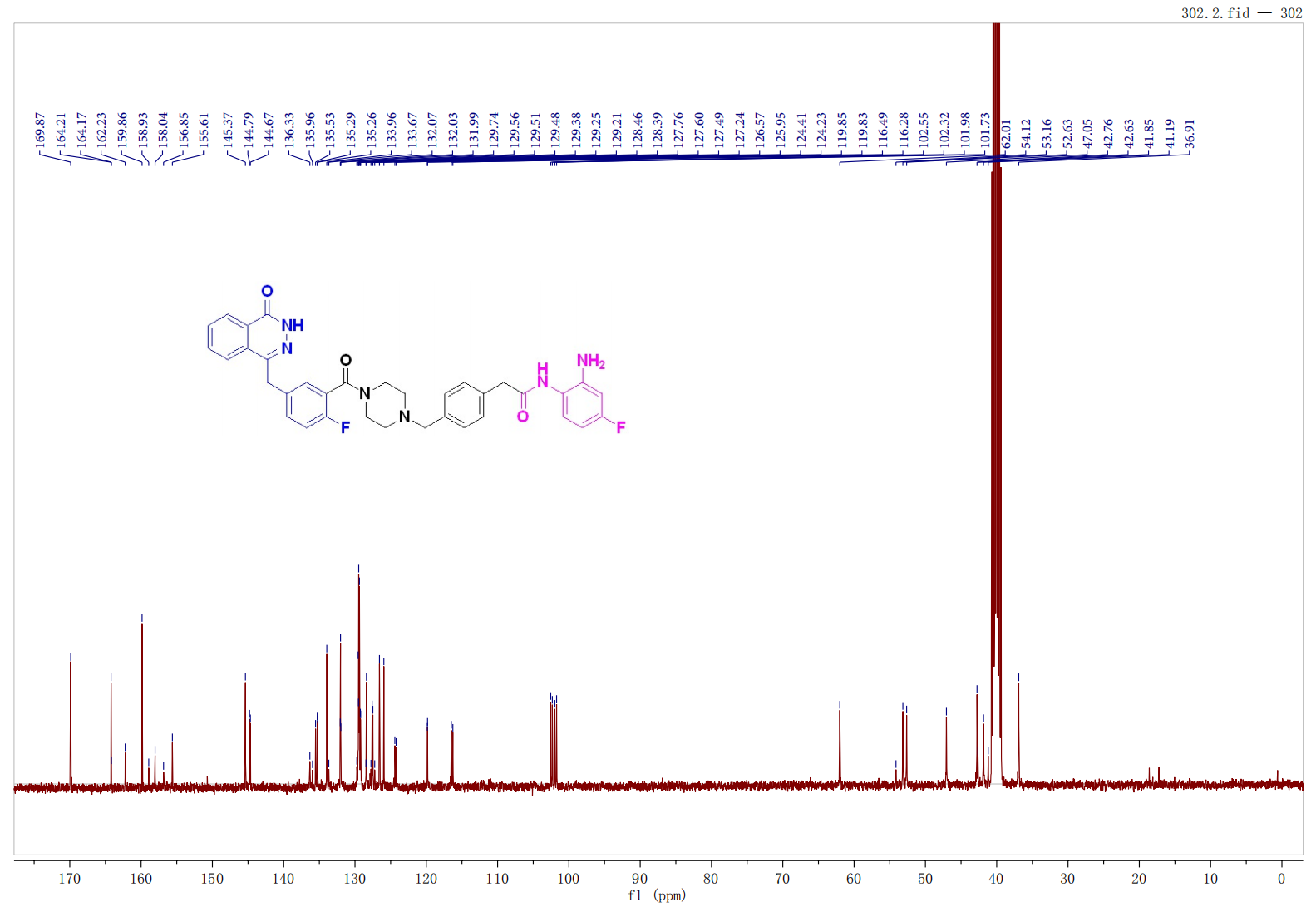


**B401**


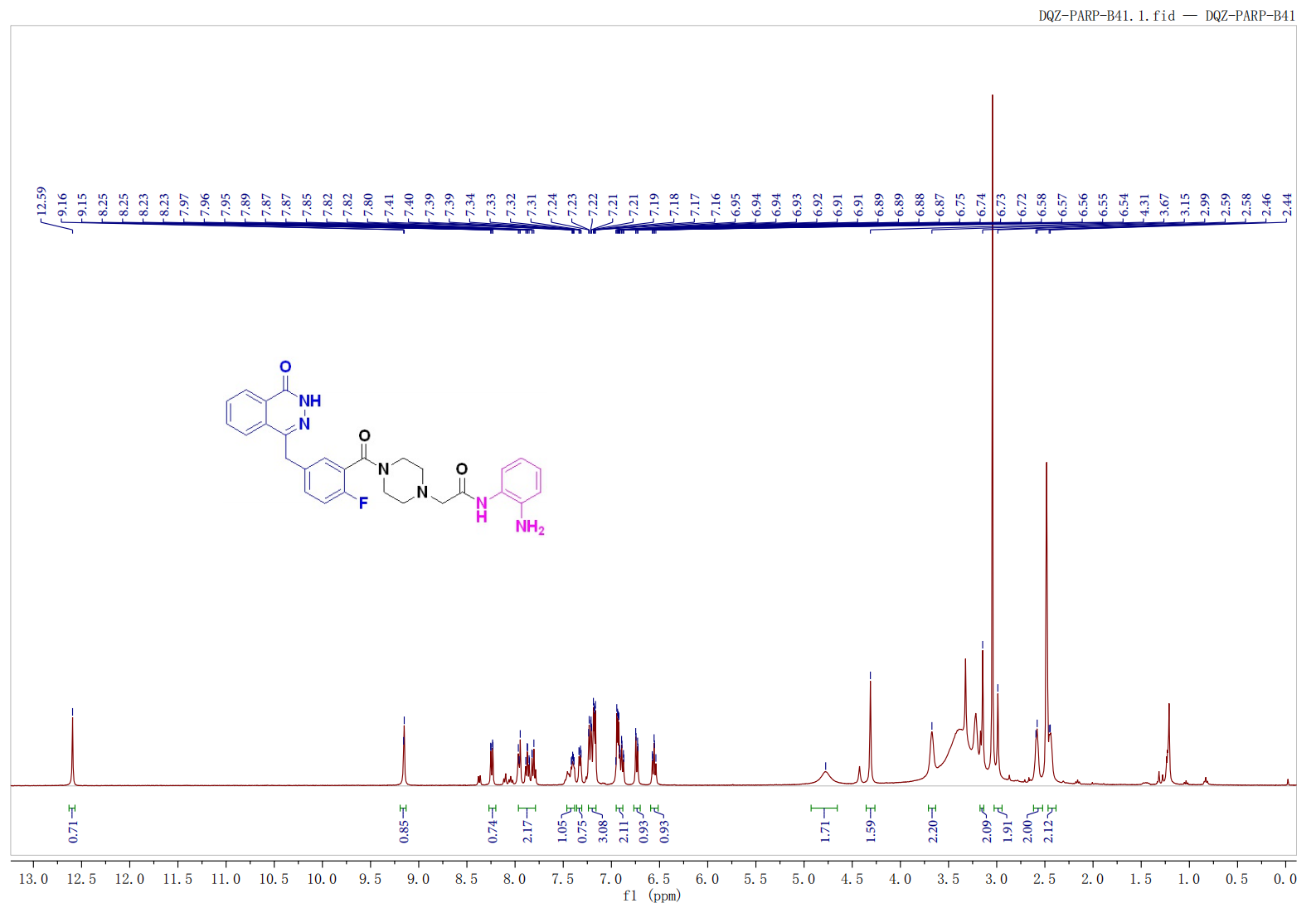


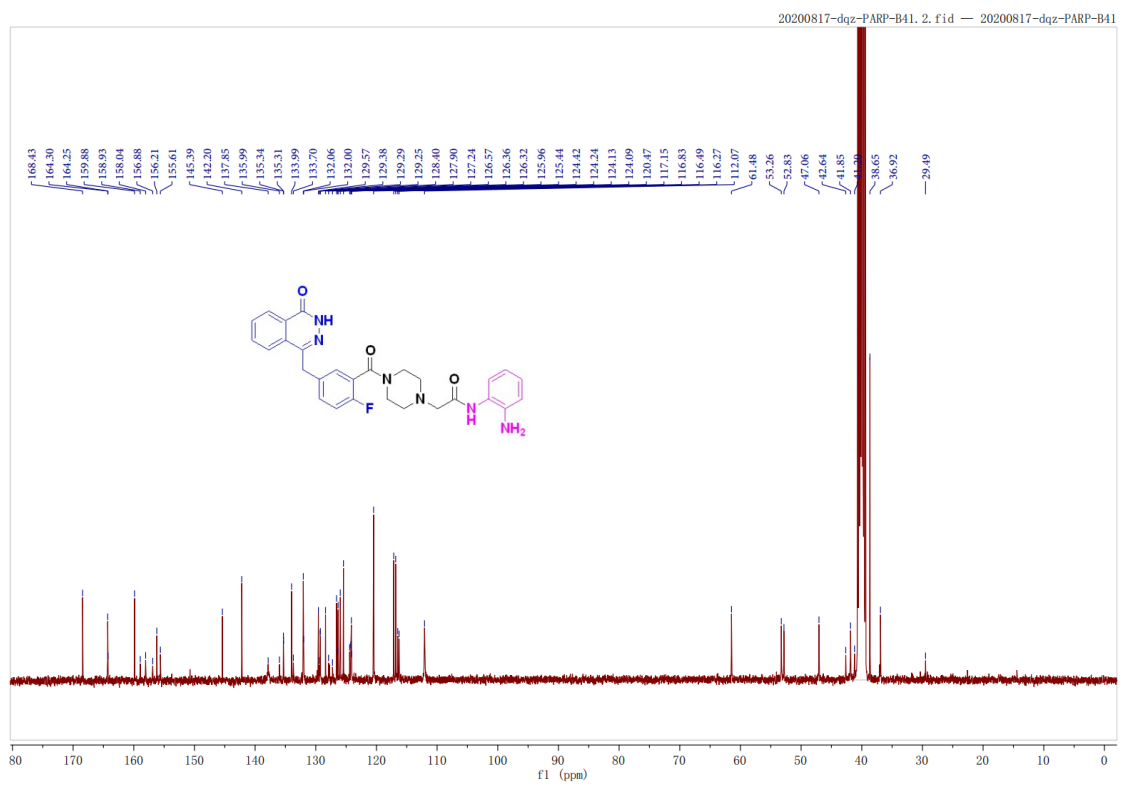


**B402**


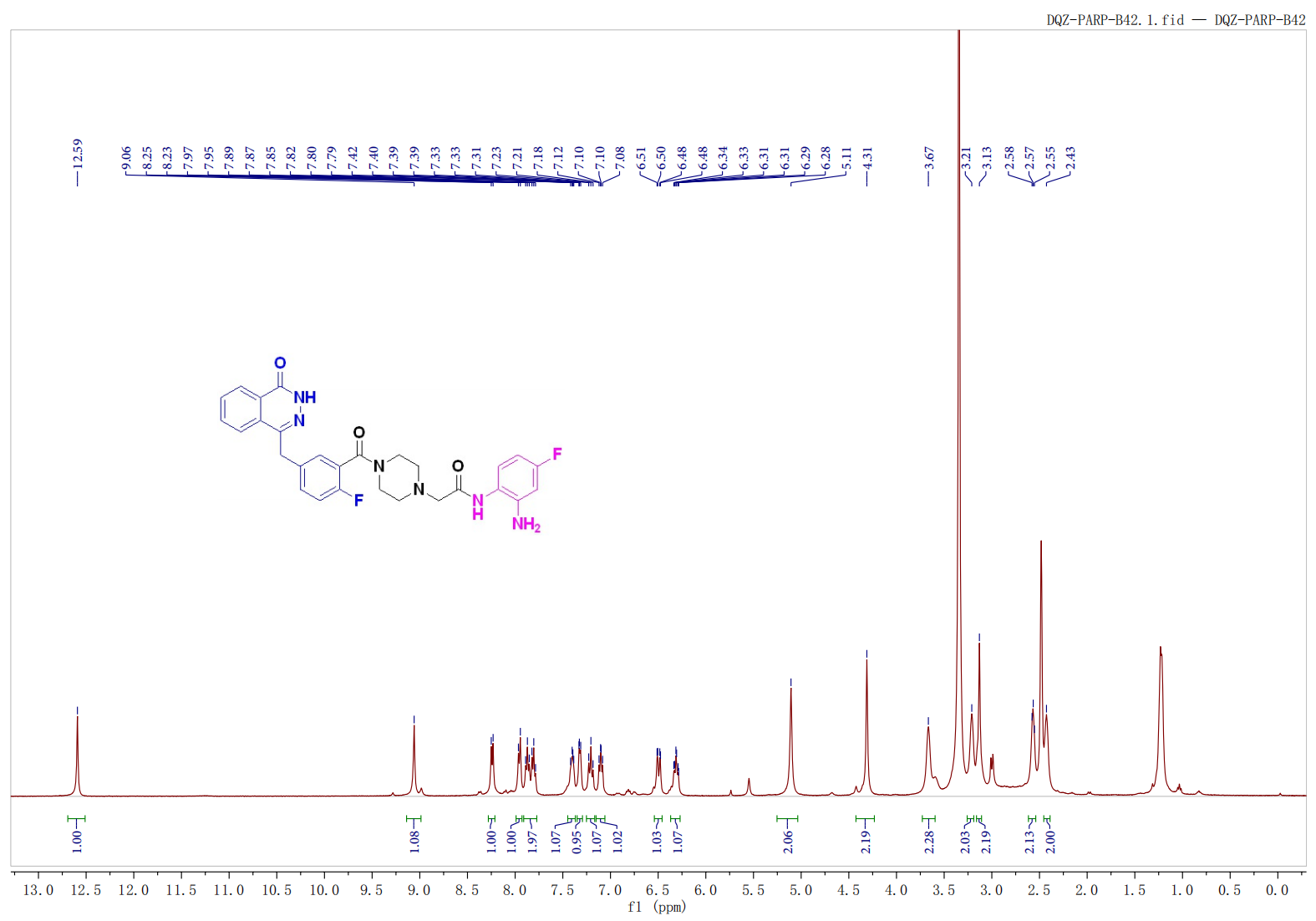


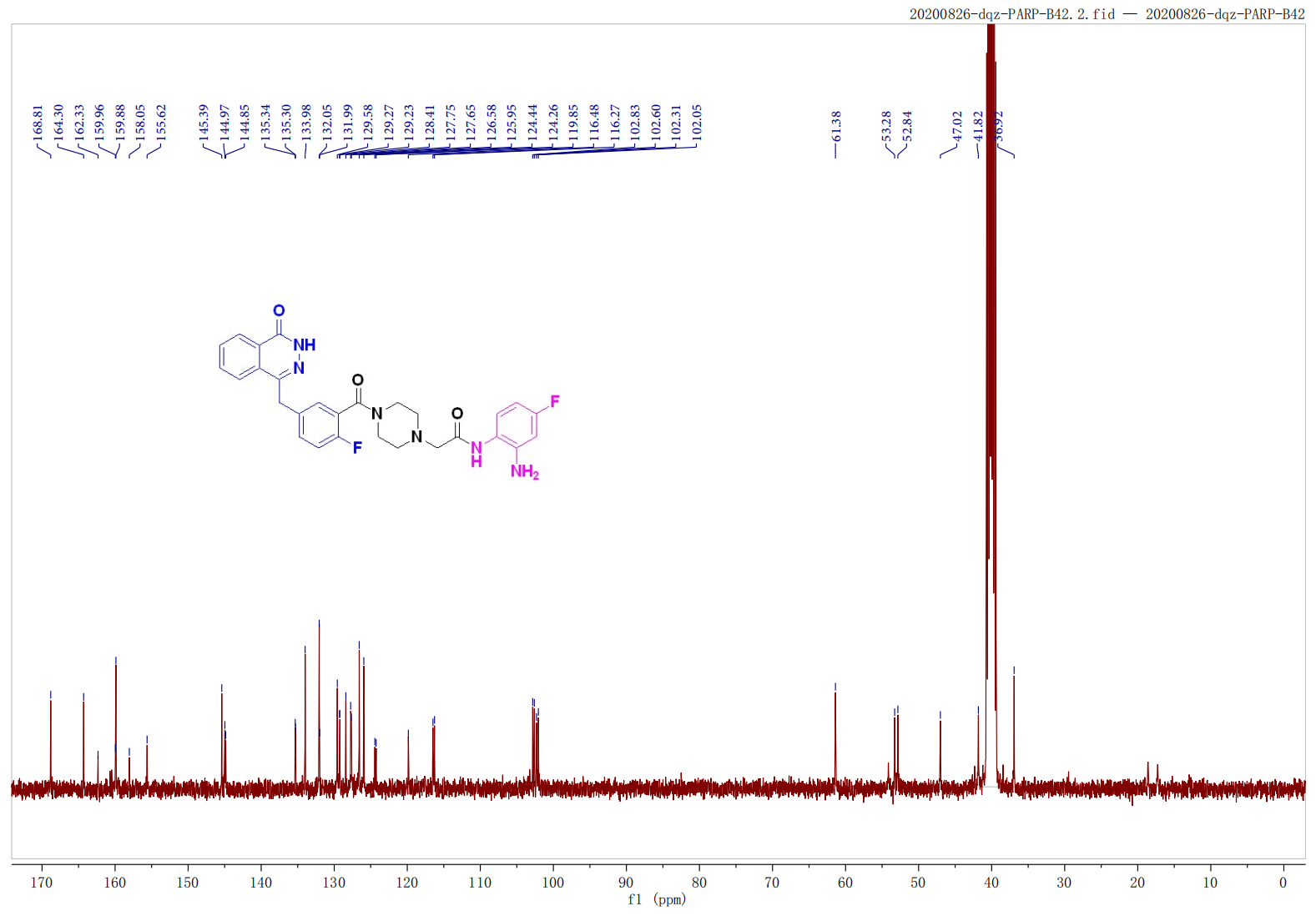


**B501**


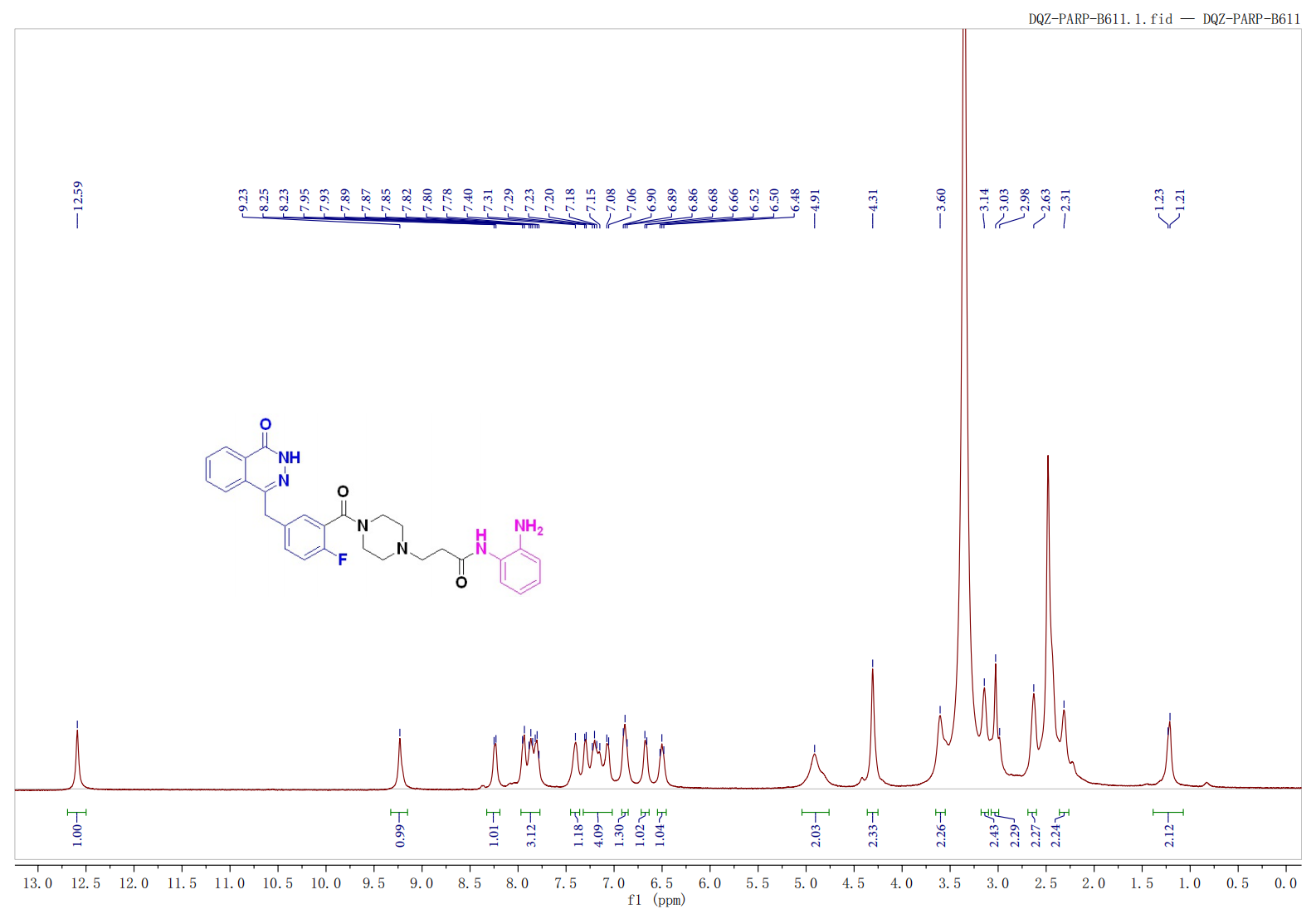


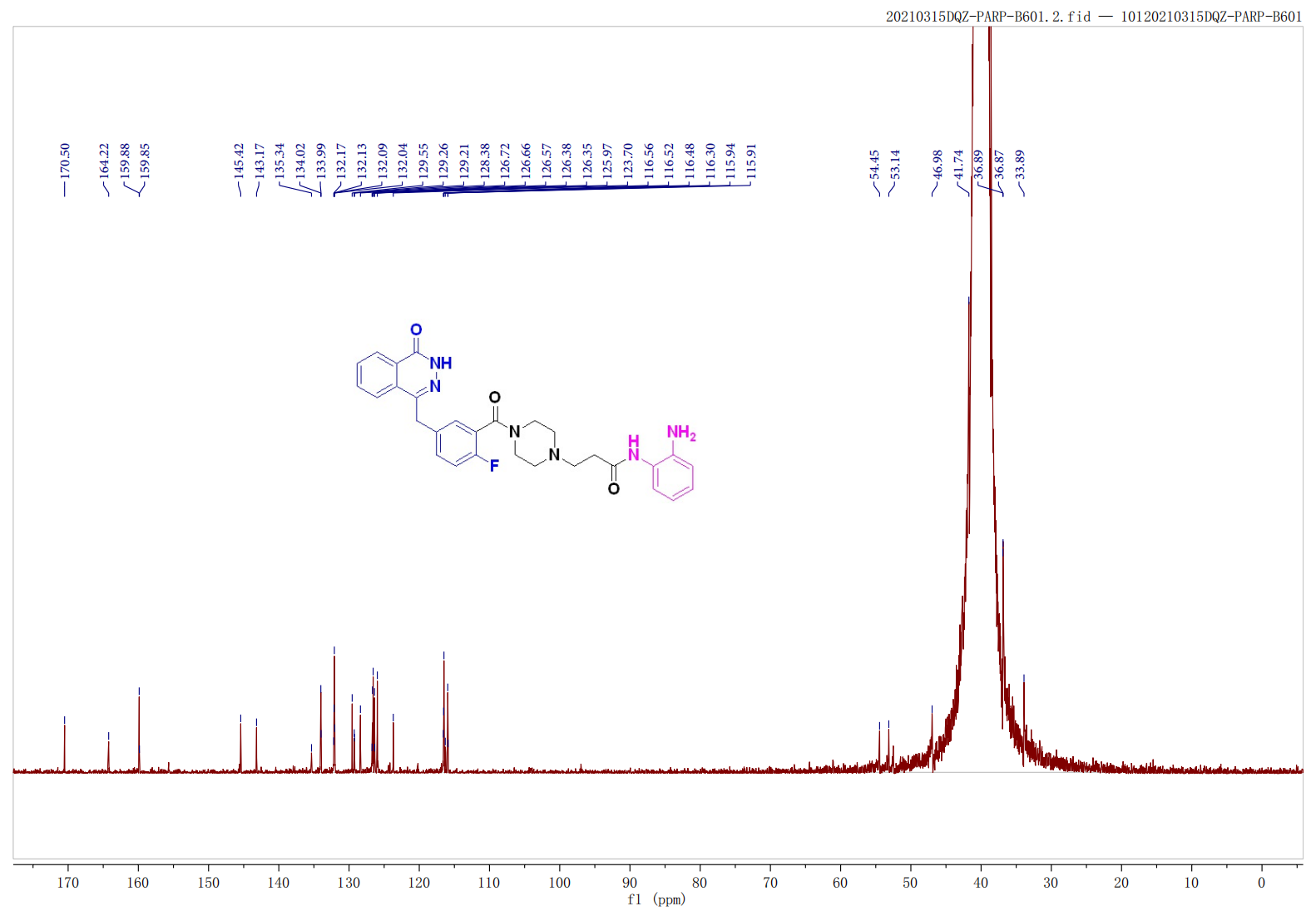


**B502**


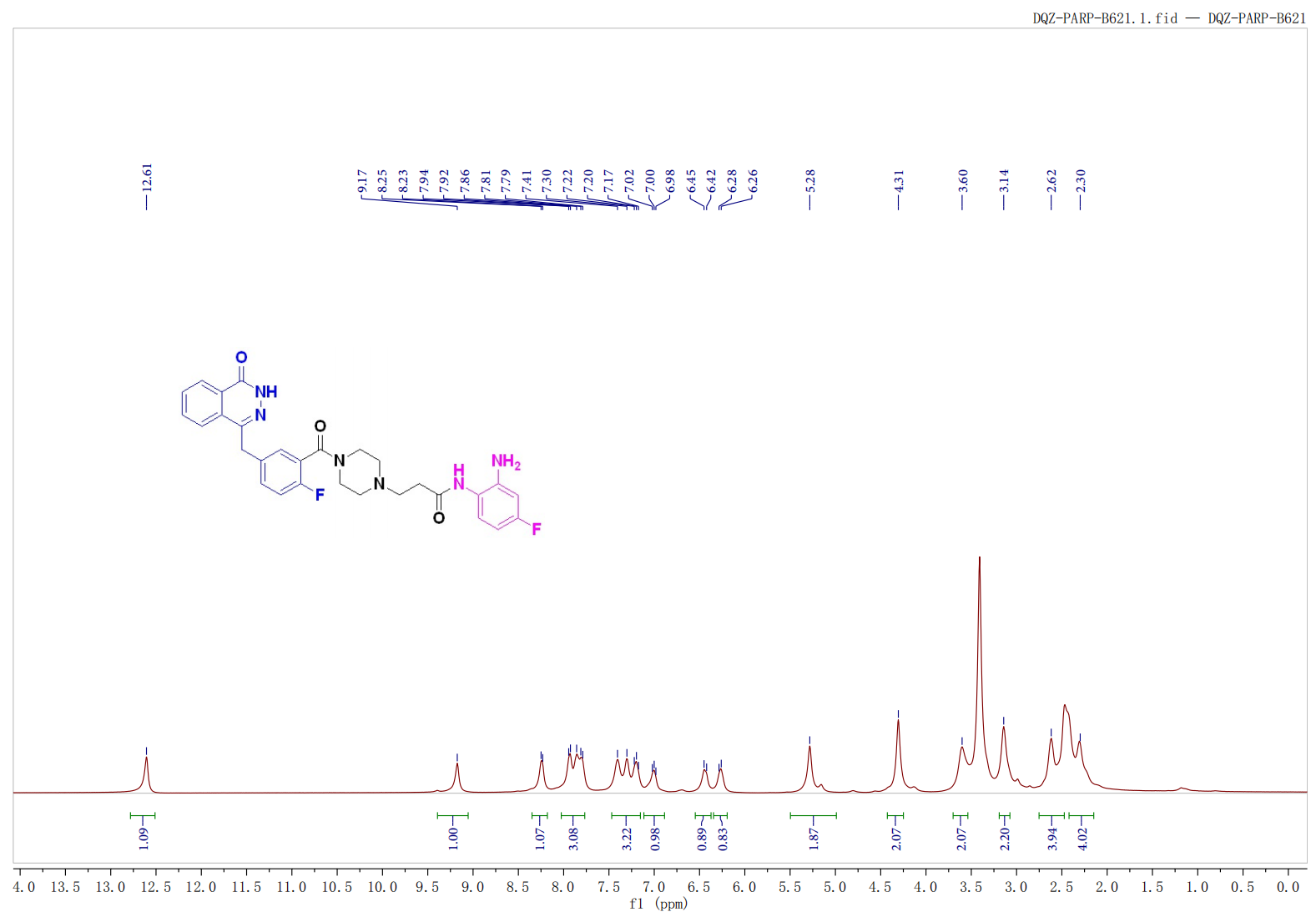


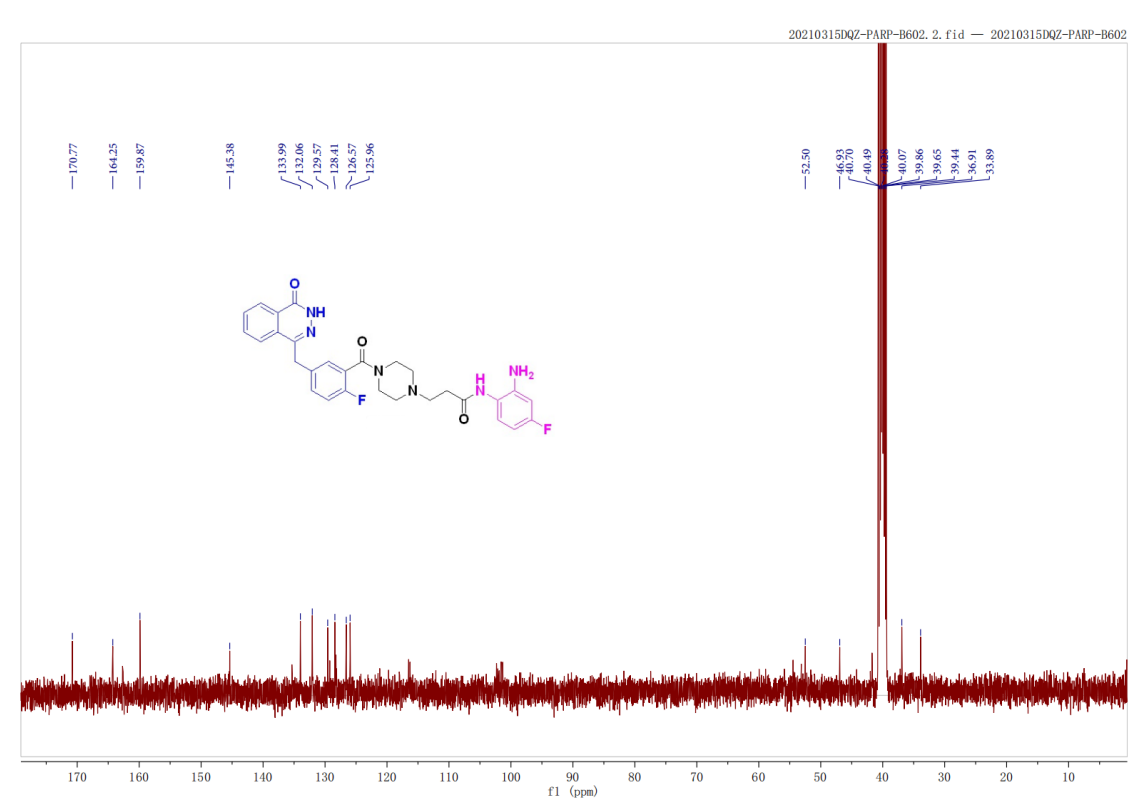


**B601**


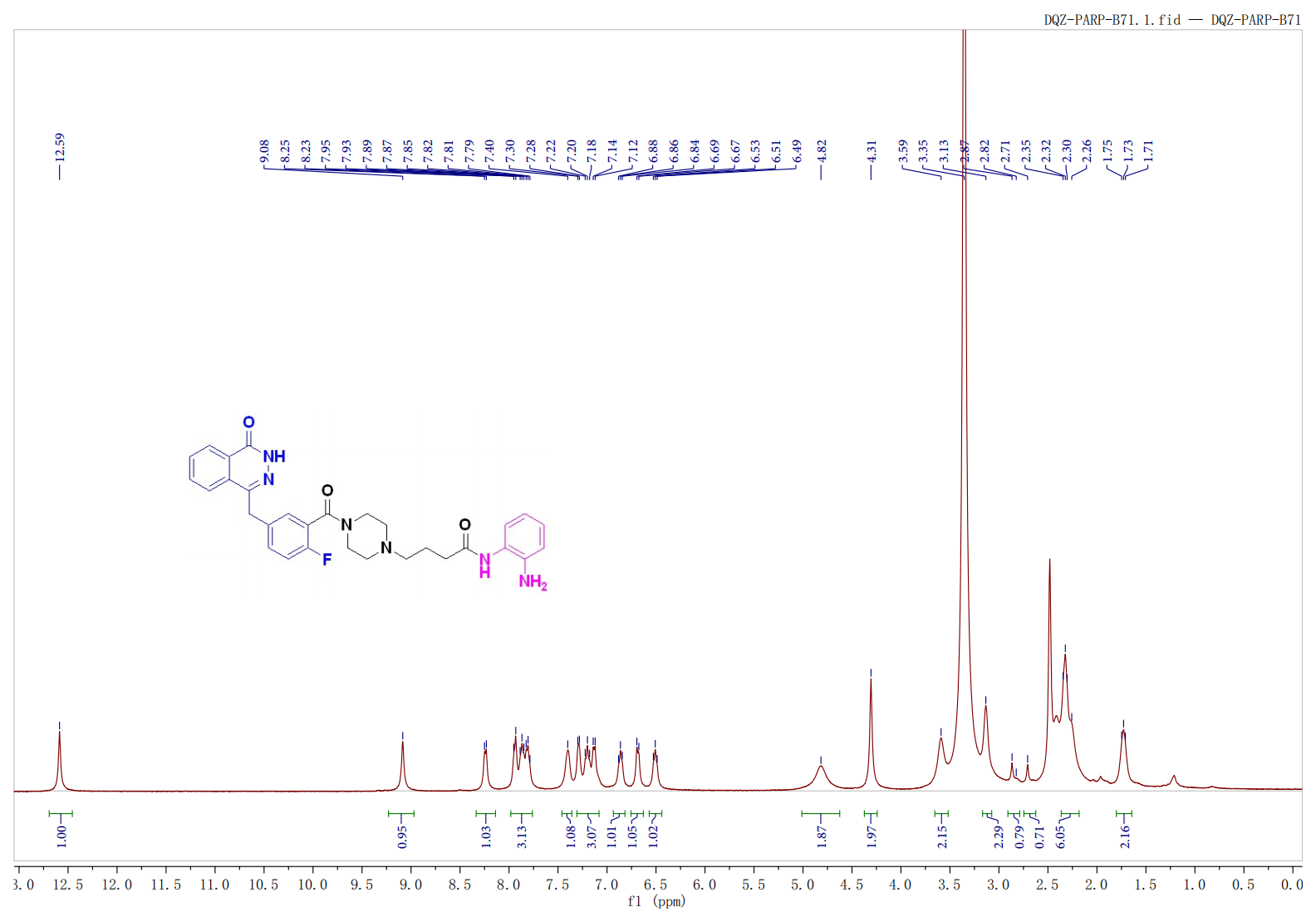


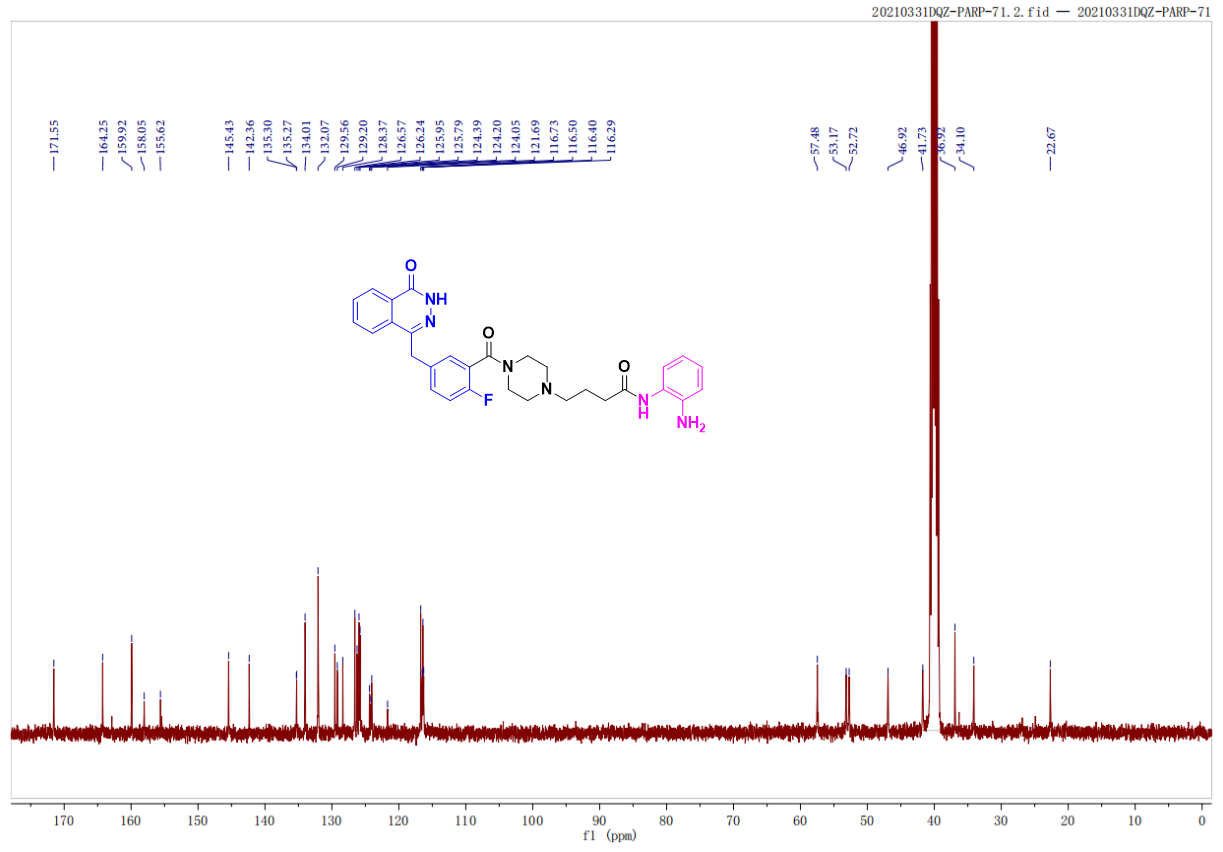


**B602**


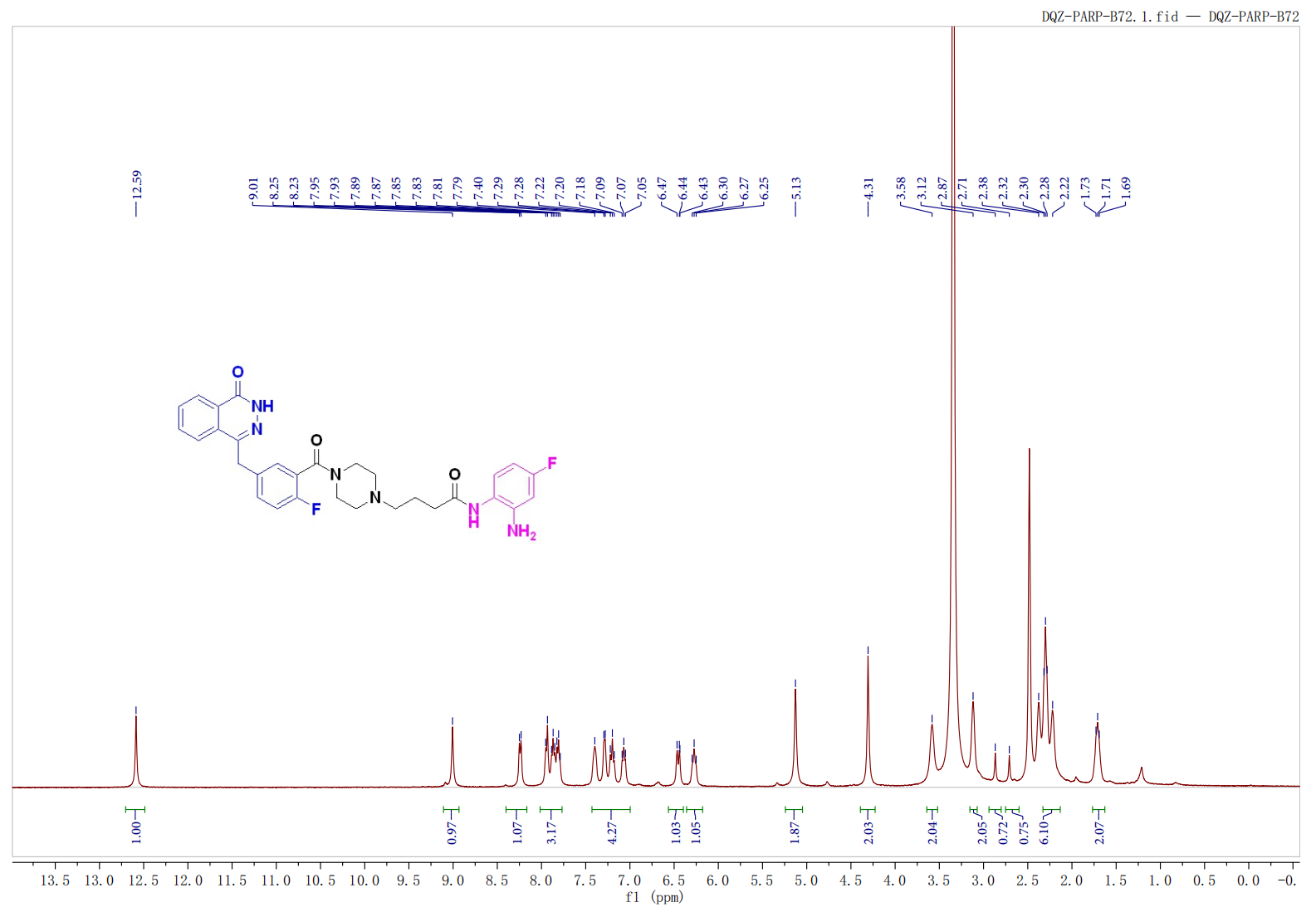


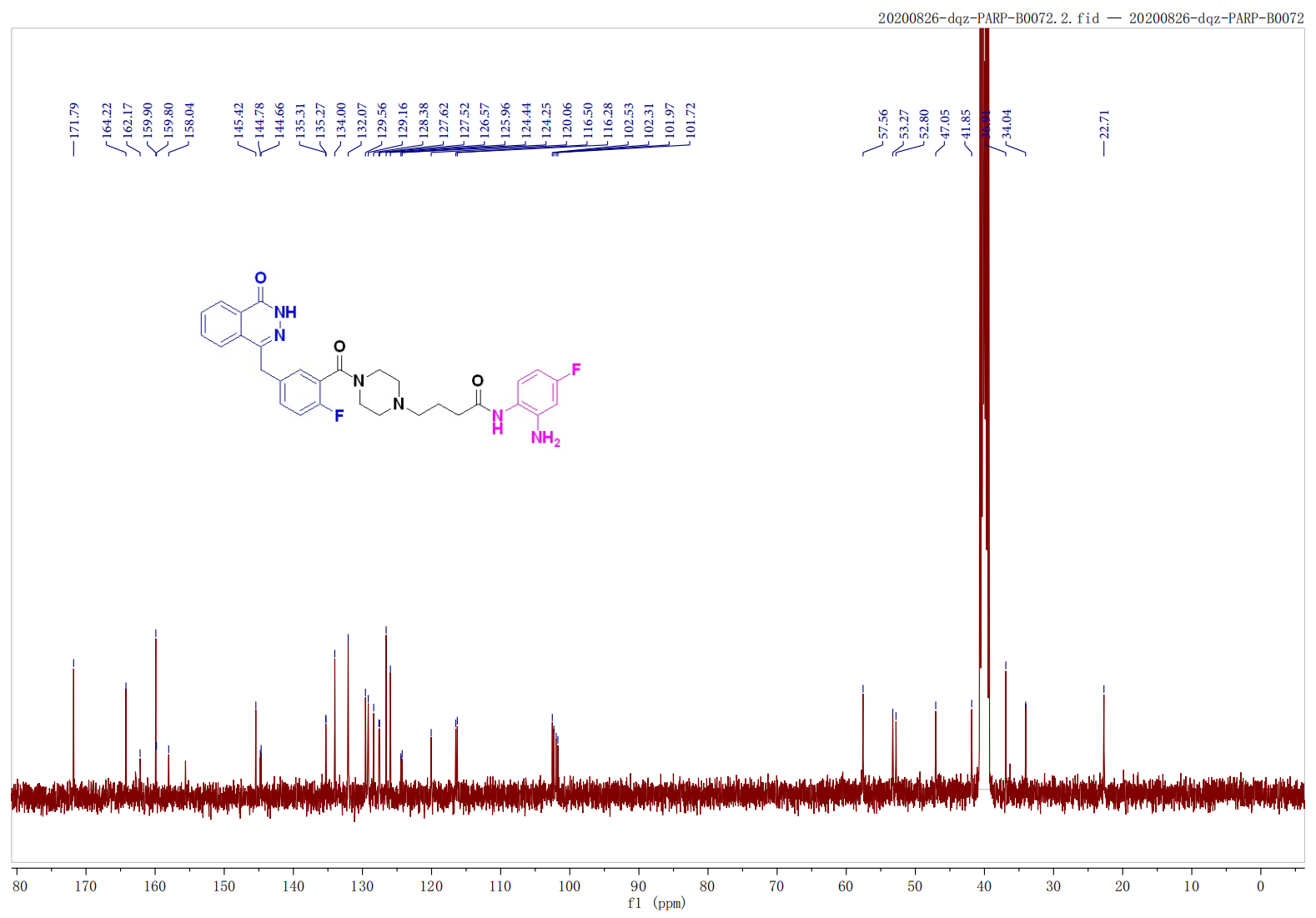


# References

1. Chu B, He A, Tian Y, He W, Chen P, Hu J*, et al.* Photoaffinity-engineered protein scaffold for systematically exploring native phosphotyrosine signaling complexes in tumor samples. Proc Natl Acad Sci U S A. 2018;115:E8863-e8872.

2. Vivian J, Rao AA, Nothaft FA, Ketchum C, Armstrong J, Novak A*, et al.* Toil enables reproducible, open source, big biomedical data analyses. Nat Biotechnol. 2017;35:314-316.

3. Liu J, Lichtenberg T, Hoadley KA, Poisson LM, Lazar AJ, Cherniack AD*, et al.* An Integrated TCGA Pan-Cancer Clinical Data Resource to Drive High-Quality Survival Outcome Analytics. Cell. 2018;173:400-416.e411.

4. Győrffy B. Survival analysis across the entire transcriptome identifies biomarkers with the highest prognostic power in breast cancer. Comput Struct Biotechnol J. 2021;19:4101-4109.

5. Ősz Á, Lánczky A, Győrffy B. Survival analysis in breast cancer using proteomic data from four independent datasets. Sci Rep. 2021;11:16787.

6. Yuan Z, Chen S, Sun Q, Wang N, Li D, Miao S*, et al.* Olaparib hydroxamic acid derivatives as dual PARP and HDAC inhibitors for cancer therapy. Bioorg Med Chem. 2017;25:4100-4109.
