# Supplementary material for: Novel dual inhibitors of PARP and HDAC induce intratumoral STING-mediated antitumor immunity in triple-negative breast cancer
Source: Cell Death Dis. 2024 Jan 5;15(1):10. doi: 10.1038/s41419-023-06303-z (PMC10770036; doi:10.1038/s41419-023-06303-z)

Figure 3A

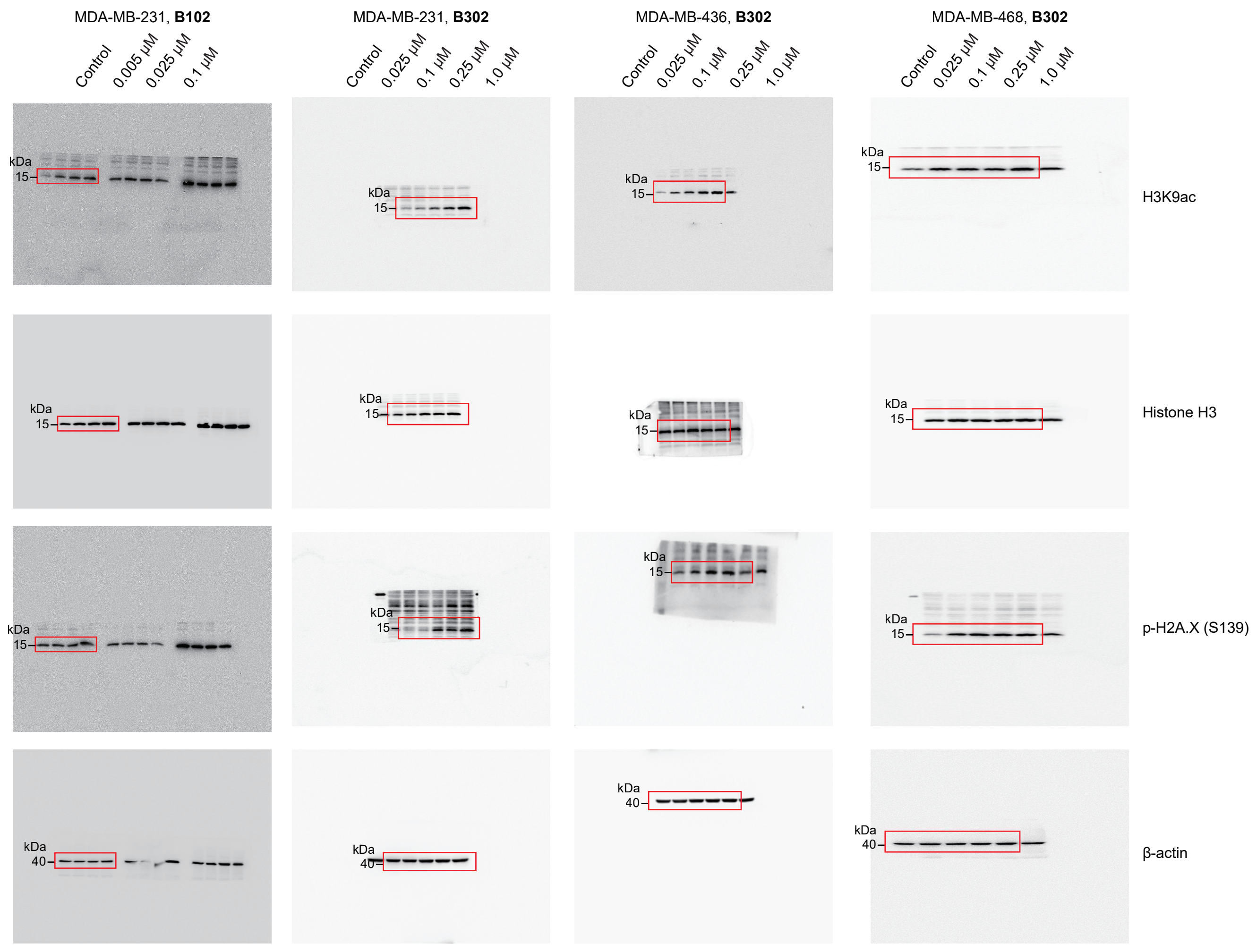

MDA-MB-231, **B102**
Control
0.005 µM
0.025 µM
0.1 µM
MDA-MB-231, **B302**
Control
0.025 µM
0.1 µM
0.25 µM
1.0 µM
MDA-MB-436, **B302**
Control
0.025 µM
0.1 µM
0.25 µM
1.0 µM
MDA-MB-468, **B302**
Control
0.025 µM
0.1 µM
0.25 µM
1.0 µM
kDa
15
40
H3K9ac
Histone H3
p-H2A.X (S139)
β-actin

Figure 3C

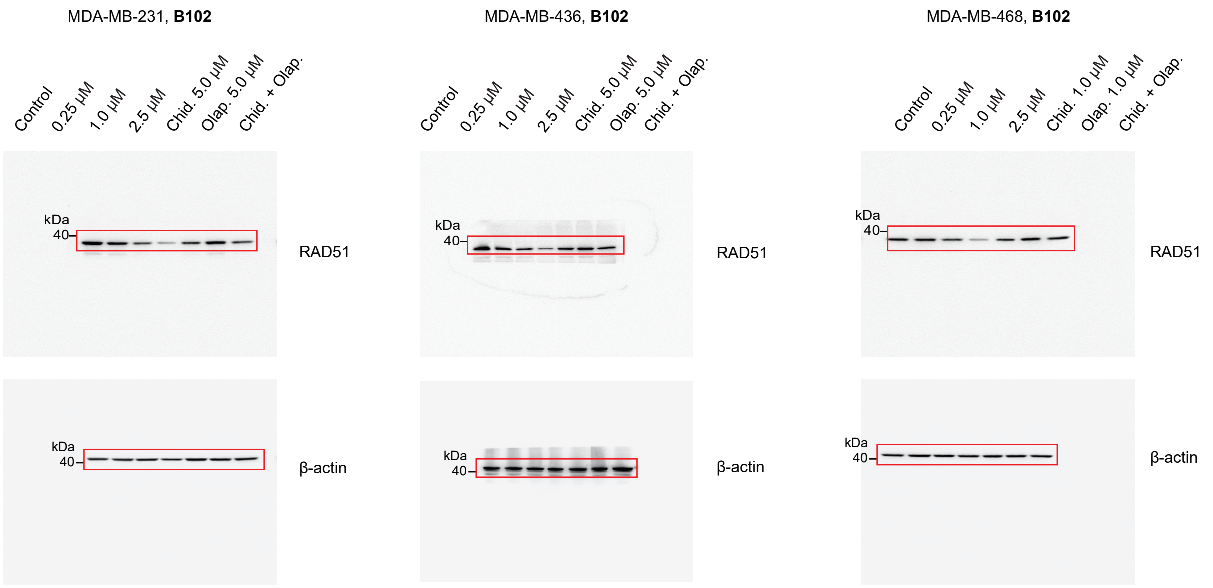

Figure 3D

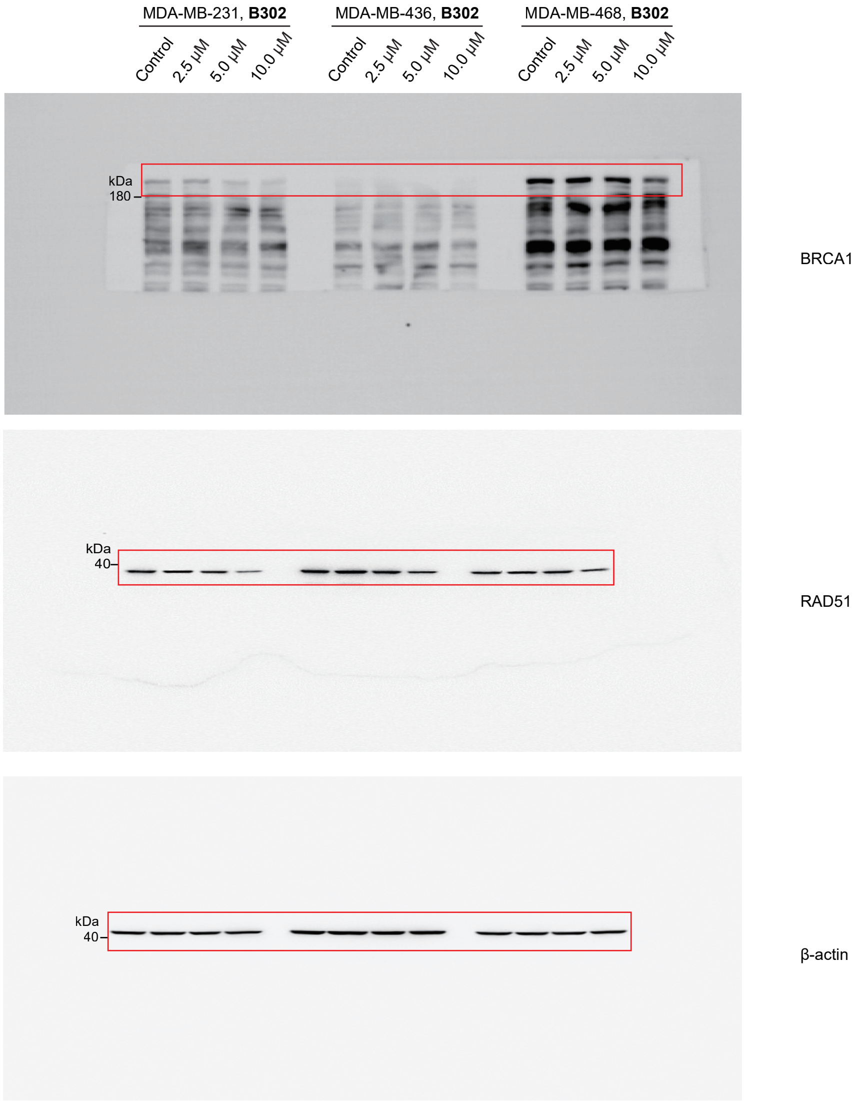

Figure 3J
B102

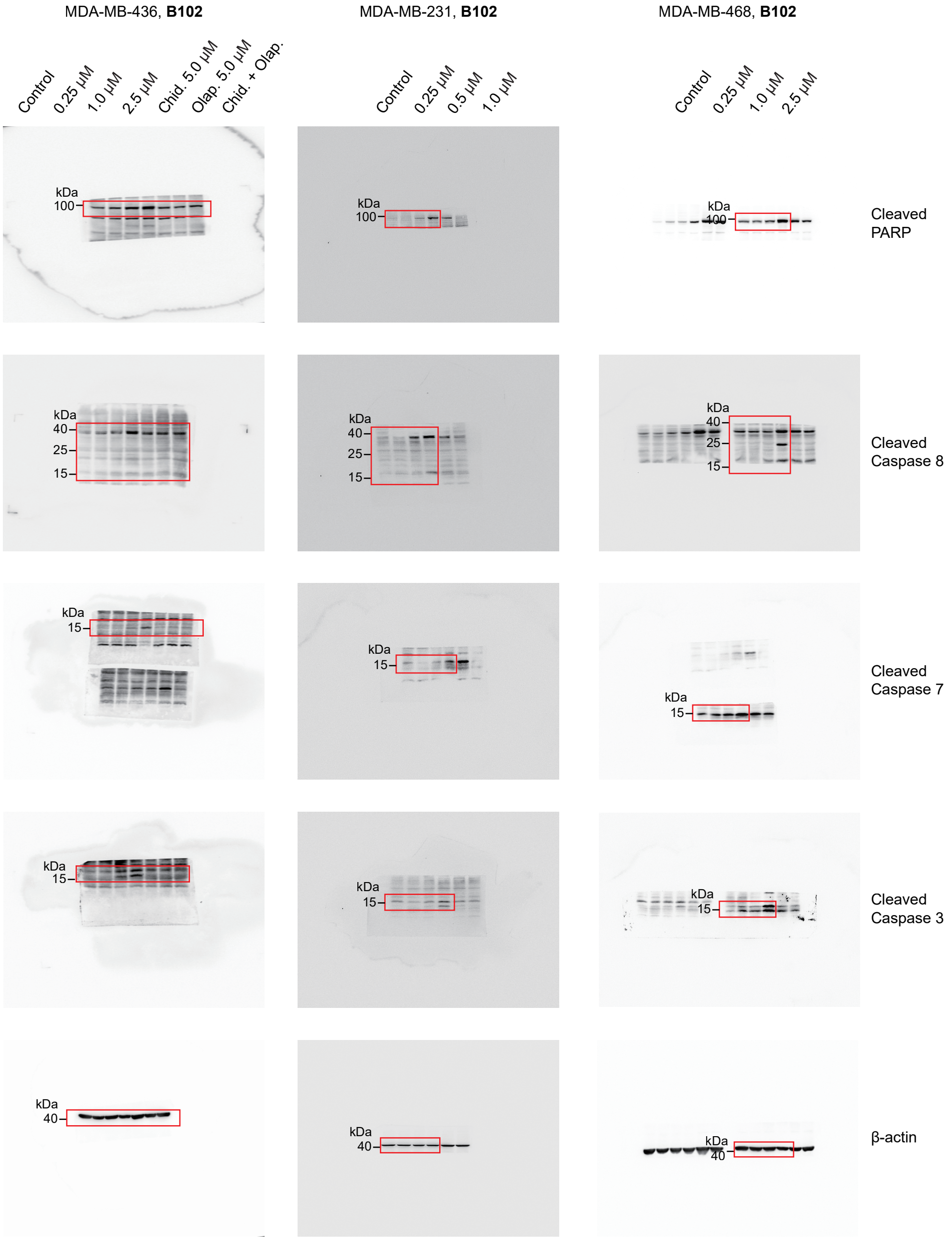

Figure 3J
B302

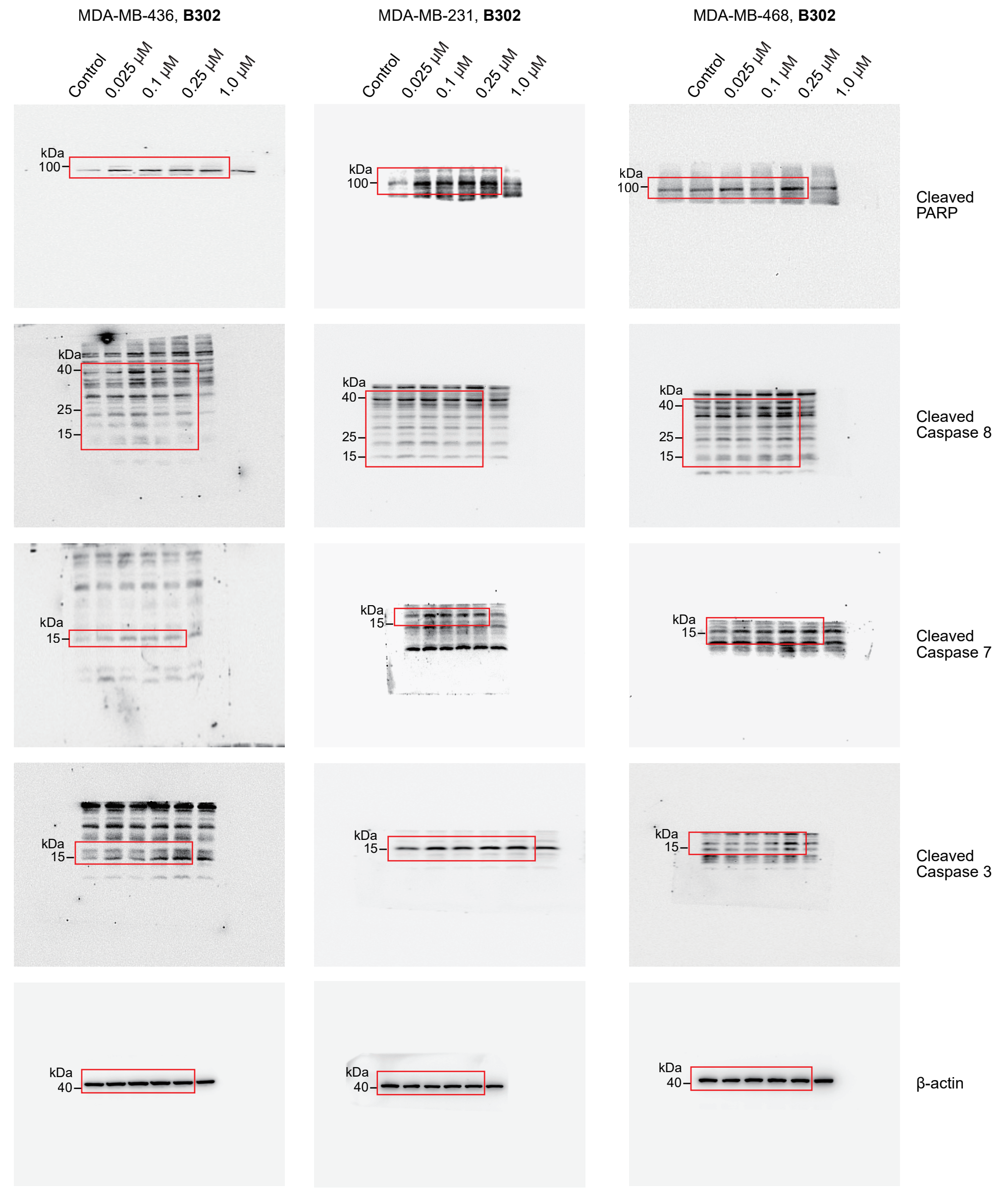

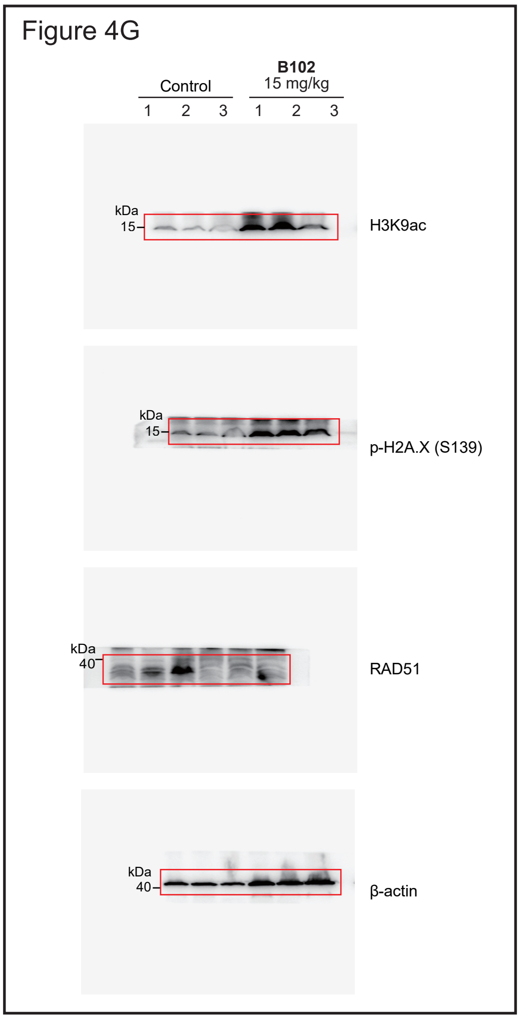

Figure 4G
B102
15 mg/kg
Control
1 2 3 1 2 3
kDa
15
H3K9ac
kDa
15
p-H2A.X (S139)
kDa
40
RAD51
kDa
40
β-actin

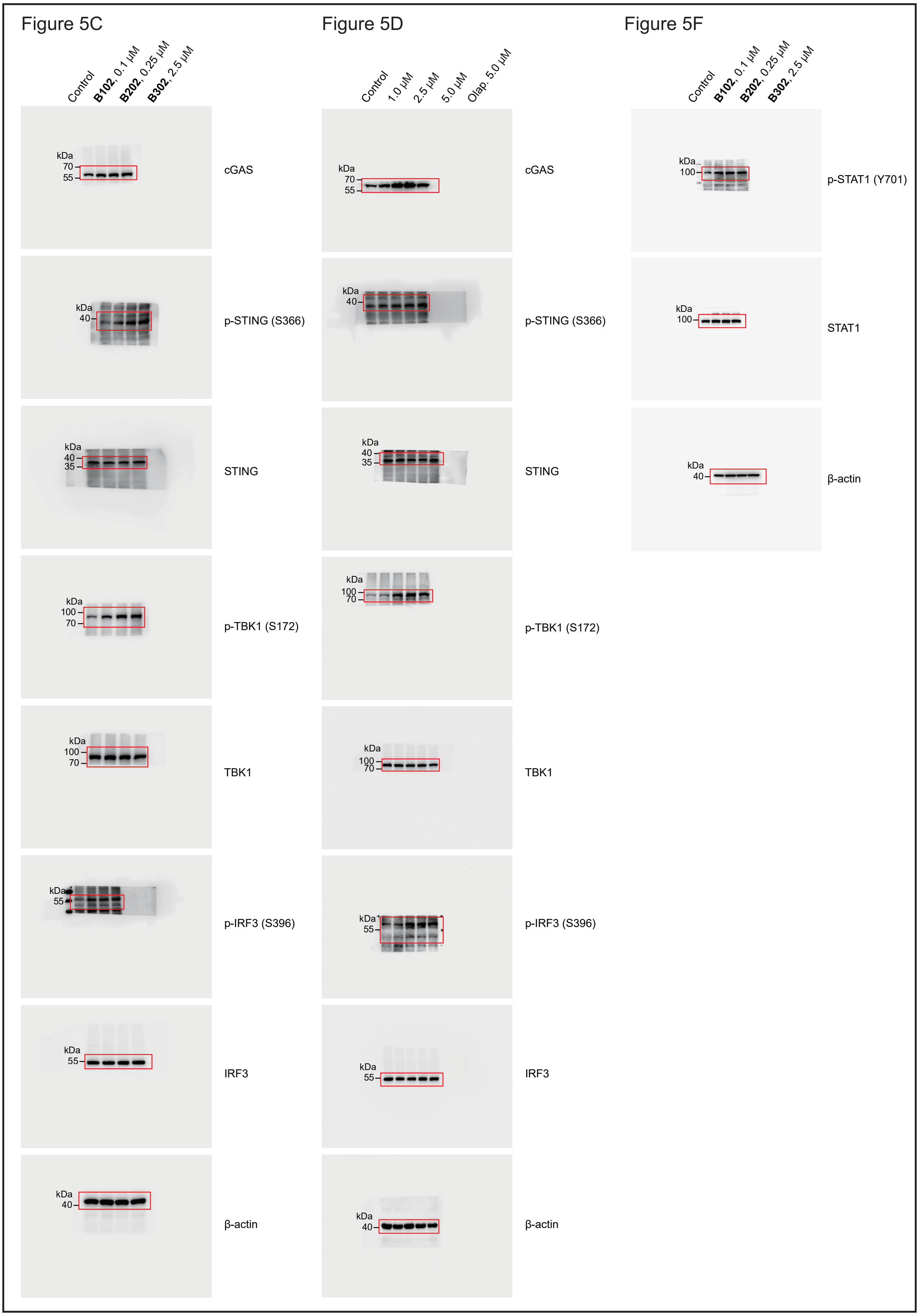

Figure 5C
Control
B102, 0.1 µM
B202, 0.25 µM
B302, 2.5 µM
kDa
70
55
cGAS
40
p-STING (S366)
35
STING
100
p-TBK1 (S172)
TBK1
p-IRF3 (S396)
IRF3
β-actin
Figure 5D
Control
1.0 µM
2.5 µM
5.0 µM
Olap. 5.0 µM
Figure 5F
p-STAT1 (Y701)
STAT1

Figure 6F

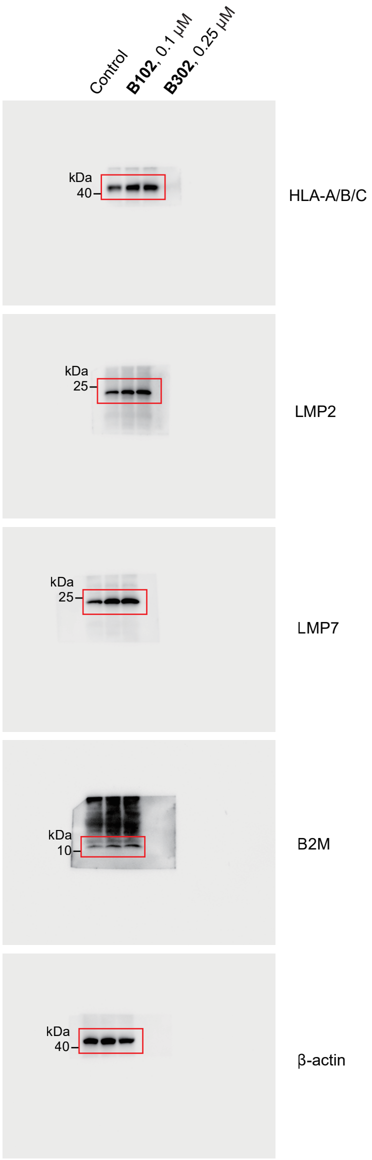

Figure 6H

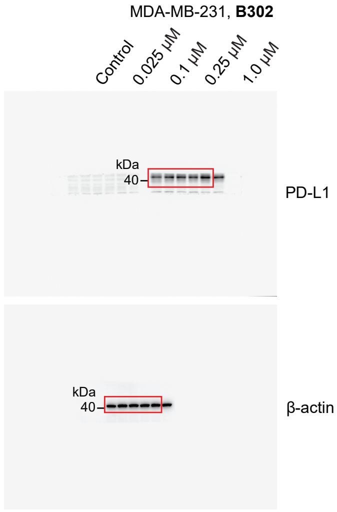

Figure S1F

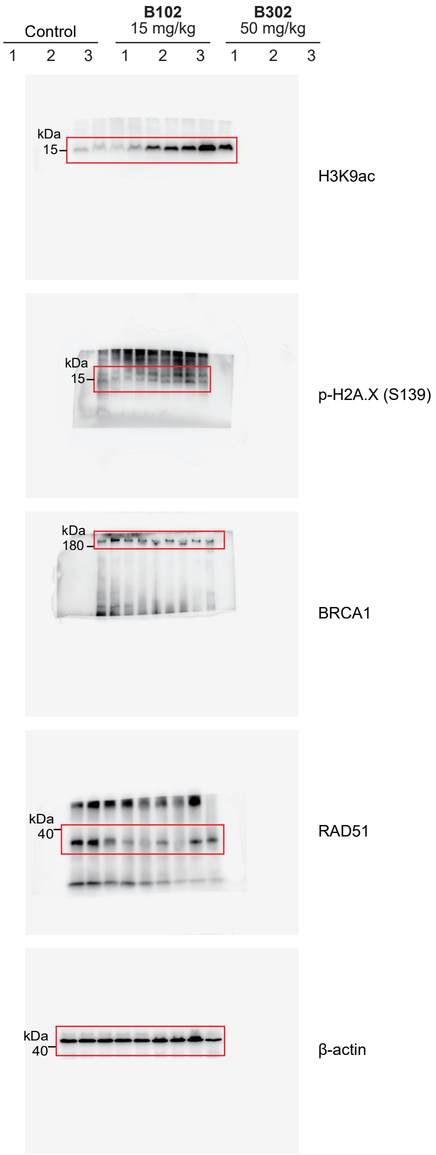

Figure S3G

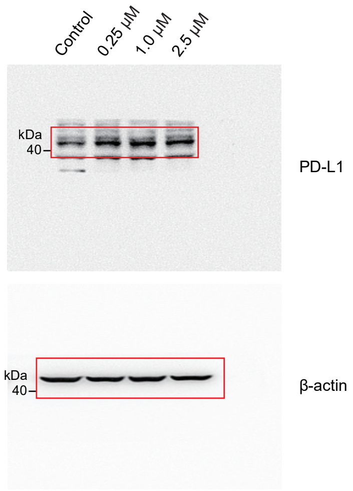

Figure S3H

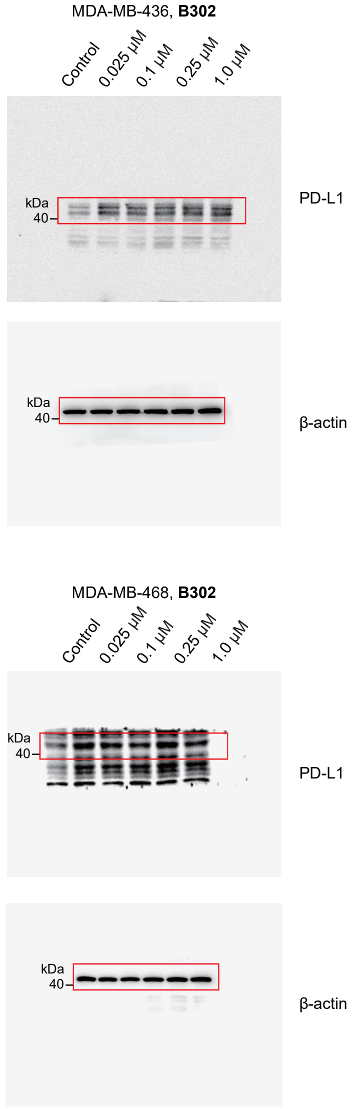

Supplement: Supplementary file 2 — Original Data File [file 41419_2023_6303_MOESM2_ESM.pdf]
